# Based on Network Pharmacology and RNA Sequencing Techniques to Explore the Molecular Mechanism of Huatan Jiangzhuo Decoction for Treating Hyperlipidemia

**DOI:** 10.1155/2021/9863714

**Published:** 2021-04-09

**Authors:** Xiaowen Zhou, Zhenqian Yan, Yaxin Wang, Qi Ren, Xiaoqi Liu, Ge Fang, Bin Wang, Xiantao Li

**Affiliations:** ^1^Laboratory of TCM Syndrome Essence and Objectification, School of Basic Medical Sciences, Guangzhou University of Chinese Medicine, Guangzhou 510006, China; ^2^Guangzhou Sagene Tech Co., Ltd., Guangzhou 510006, China; ^3^College of Traditional Chinese Medicine, Hunan University of Chinese Medicine, Changsha 410208, China; ^4^Shenzhen Traditional Chinese Medicine Hospital, Shenzhen 518000, China

## Abstract

**Background:**

Hyperlipidemia, due to the practice of unhealthy lifestyles of modern people, has been a disturbance to a large portion of population worldwide. Recently, several scholars have turned their attention to Chinese medicine (CM) to seek out a lipid-lowering approach with high efficiency and low toxicity. This study aimed to explore the mechanism of Huatan Jiangzhuo decoction (HTJZD, a prescription of CM) in the treatment of hyperlipidemia and to determine the major regulation pathways and potential key targets involved in the treatment process.

**Methods:**

Data on the compounds of HTJZD, compound-related targets (C-T), and known disease-related targets (D-T) were collected from databases. The intersection targets (I-T) between C-T and D-T were filtered again to acquire the selected targets (S-T) according to the specific index. Gene ontology (GO) and Kyoto Encyclopedia of Genes and Genomes (KEGG) pathway enrichment, as well as network construction, were applied to predict the putative mechanisms of HTJZD in treating hyperlipidemia. Thereafter, an animal experiment was conducted to validate the therapeutic effect of HTJZD. In addition, regulated differentially expressed genes (DEGs) were processed from the RNA sequencing analysis results. Common genes found between regulated DEGs and S-T were analyzed by KEGG pathway enrichment to select the key targets. Lastly, key targets were validated by real-time quantitative reverse transcription PCR (qRT-PCR) analysis.

**Results:**

A total of 210 S-T were filtered out for enrichment analysis and network construction. The enrichment results showed that HTJZD may exert an effect on hyperlipidemia through the regulation of lipid metabolism and insulin resistance. The networks predict that the therapeutic effect of HTJZD may be based on the composite pharmacological action of these active compounds. The animal experiment results verify that HTJZD can inhibit dyslipidemia in rats with hyperlipidemia, suppress lipid accumulation in the liver, and reverse the expression of 202 DEGs, which presented an opposite trend in the model and HTJZD groups. Six targets were selected from the common targets between 210 S-T and 202 regulated DEGs, and the qRT-PCR results showed that HTJZD could effectively reverse Srebp-1c, Cyp3a9, and Insr mRNA expression (*P* < 0.01).

**Conclusion:**

In brief, network pharmacology predicted that HTJZD exerts a therapeutic effect on hyperlipidemia. The animal experimental results confirmed that HTJZD suppressed the pathological process induced by hyperlipidemia by regulating the expression of targets involved in lipid metabolism and insulin resistance.

## 1. Introduction

Hyperlipidemia is a condition wherein lipid-glucose metabolic disruption occurs, and it is mainly characterized by the derangements of lipoproteins circulating in the blood, such as high levels of total cholesterol (TC), triglyceride (TG) and/or low-density lipoprotein cholesterol (LDL-c), or aberrant declined level of low-density lipoprotein cholesterol (HDL-c) [1, 2]. Growing evidence has proven that hyperlipidemia could hardly avoid developing cardiovascular disease, nephropathy, type 2 diabetes mellitus, and nonalcoholic fatty liver disease and would even elevate the cerebellar toxicity especially for the cholesterol overload [3–5]. Hyperlipidemia is an epidemic disease worldwide, with the number of expected cases reaching 78 million by 2022 in major countries, playing an important role in high morbidity and mortality of cardiovascular diseases [6]. Lifestyle improvements and drug interventions should be under discussion to limit the progress of hyperlipidemia.

Currently, statins, the first-line recommended therapy for lowering lipid, are the widely used drugs for hyperlipidemia [7]. Nevertheless, accompanying the favorable effects of reducing cholesterol biosynthesis, statins cause adverse effects, such as myalgia, hepatic and renal toxicity, and cognitive disorder [8]. It seems that the Pcsk9 inhibitor, a new agent, is beneficial as it lowers the LDL levels [9]. However, the high cost and the side effects of the Pcsk9 inhibitor cannot be ignored [10, 11]. Accordingly, several researchers have made efforts to seek help from CM, which is a healthcare-focused medicine system with abundant practice experience. In the CM theory, lipid deposition is a pathological phenomenon of phlegm and blood blocking of the veins, which results from the dysfunction of the liver, incapability of transportation and transformation of the spleen, and irregularity of fluid metabolism [12–14]. Contemporary experts have found that treating hyperlipidemia based on the guidance of the CM theory could be possible. Peng et al. [15] discovered that decoctions, characterized by tonifying spleen and resolving phlegm, exerted a curative effect on turbid phlegm syndrome of hyperlipidemia. Wang et al. [13] stated that decoctions that clear the liver and eliminate dampness could increase bile secretion and reduce cholesterol absorption.

Huatan Jiangzhuo decoction (HTJZD) was termed based on the therapeutic method of resolving phlegm and reducing turbidity of the CM theory. It includes the following nine herbs: Poria, Alismatis Rhizoma, Atractylodis Macrocephalae Rhizoma, Atractylodis Rhizoma, Pinelliae Rhizoma Praeparatum, Citri Reticulatae Pericarpium, Glycyrrhizae Radix et Rhizoma, Ginseng Radix et Rhizoma, and Citri Reticulatae Pericarpium Viride. It has been reported that hypolipidemic efficacy is found in a portion of ingredients in these herbs. For example, *Alisma orientale* could increase the HDL-c/LDL-c ratio and regulate abnormal cholesterol-related markers in high-fat diet (HFD)-fed rats [16]. *Poria cocos* attenuates the perturbations of bile acid biosynthesis [17]. Moreover, immature *Citrus reticulata* extract could ameliorate HFD-induced obesity by initiating browning of beige adipocytes [18]. In addition, our previous studies [19, 20] have suggested that HTJZD could suppress the pathological process of hyperlipidemia, but the underlying mechanism has not been fully explained.

This study resorted to network pharmacology combined with RNA sequencing techniques to promote our understanding concerning the molecular mechanism of HTJZD in treating hyperlipidemia. Network pharmacology is a novel bioinformatic technology that is applied to elucidate the occurrence and development of diseases based on system biology and biological network equilibrium. It is also generally used to guide the discovery of new drugs and clarify the mechanism of CM [21]. The RNA sequencing technology is a dynamic connection between the species' genome and its external physiological status, indicating the quality and quantity of gene expression in the specific organ at a specific physiological stage [22]. Combining these techniques to conduct bioinformatic research provides more possibility of exploring the therapeutic mechanism of HTJZD.

## 2. Materials and Methods

### 2.1. Prediction of Putative Targets of Compounds in HTJZD

The compounds of each herb in HTJZD were collected from TCMSP (https://tcmspw.com/tcmsp.php). TCMSP is a composite website that contains information about active compounds, potential targets, related diseases, and pharmacodynamics data of CM. ADME parameters were used to evaluate the compounds, of which are oral bioavailability (OB)≥30% and drug likeness (DL) of ≥0.18 were the inclusion criteria used to select the active compounds of HTJZD in this study. Subsequently, the related targets of selected compounds were obtained from TCMSP, ETCM (http://www.tcmip.cn/ETCM/index.php/Home/Index/), and Swiss Target Prediction (http://www.swisstargetprediction.ch/). Candidate target genes from ETCM should meet the prediction confidence index of ≥0.80. Targets of compounds predicted by Swiss Target Prediction should attain the canonical SMILEs or InChI from PubChem (https://pubchem.ncbi.nlm.nih.gov/) first and then be pasted in the website to acquire ones with the probability of ≥0.

### 2.2. Collecting Known Hyperlipidemia-Related Targets

Hyperlipidemia targets were collected from DisGeNET (https://www.disgenet.org/) and GeneCards (https://www.genecards.org/) with “hyperlipidemia” as the keyword. DisGeNET contains a large number of genes and variants related to human diseases and serves as a multifunctional platform to meet diverse research purposes. GeneCards is a composite database that provides known and predicted genes of *Homo sapiens* regarding multiple omics and functional information.

### 2.3. Network Construction and Analysis

Compound-related targets (C-T) and disease-related targets (D-T) were standardized in UniProtKB (https://www.uniprot.org/) based on *H. sapiens* genes to delete duplicate values. Then, the intersection targets (I-T) of C-T and D-T were searched and used to explore the correlation among I-T on STRING (https://www.string-db.org/). The minimum required interaction score was set as “highest confidence (0.900)” on STRING, and the simple tabular text output was downloaded from it. Lastly, the node (selected targets, S-T) interaction meeting the criteria of “a combined score of ≥0.95” was screened out to construct the herb-compound-S-T network and S-T-pathway network in Cytoscape 3.7.2 for visualizing the results.

### 2.4. GO Functional Annotation and KEGG Pathway Enrichment Analysis

S-T were input to g:Profiler (https://biit.cs.ut.ee/gprofiler/gost) for KEGG signaling pathway enrichment analysis and GO functional annotation including biological process (BP), cellular component (CC), and molecular function (MF). The website, g:Profiler, updated in a timely manner, serves as a collection of functional enrichment analysis (g:Gost), gene ID conversion (g:Convert), and SNP id to gene name (g:SNPense). The GO results were depicted by GraphPad Prism 8, and the KEGG pathway results were painted by ggplot2 packages in R Studio (version 4.0.2).

Animal experiments were conducted to evaluate the therapeutic effects of HTJZD for treating hyperlipidemia and validate the potential key targets for efficacy.

### 2.5. Chemicals and Reagents

The detection kits for TC, TG, LDL-c, and HDL-c were purchased from the Nanjing Jiancheng Bioengineering Institute (Nanjing, China). HE staining and Oil Red O staining associated reagents were purchased from Sinopharm Chemical Reagent Co., Ltd (Shanghai, China) and Wuhan Servicebio Technology Co., Ltd (Wuhan, China), respectively. Bestar qPCR RT kit (batch number: 2220) and SYBR Green qPCR Master Mix kit (batch number: 2043) were produced by DBI Bioscience Co., Ltd. (batch number: H97451, specification: 20 mg/tablet). Atorvastatin was diluted to 0.72 mg/mL in distilled water and preserved at 4°C.

### 2.6. Preparation of HTJZD Extraction

**Figure 1:**
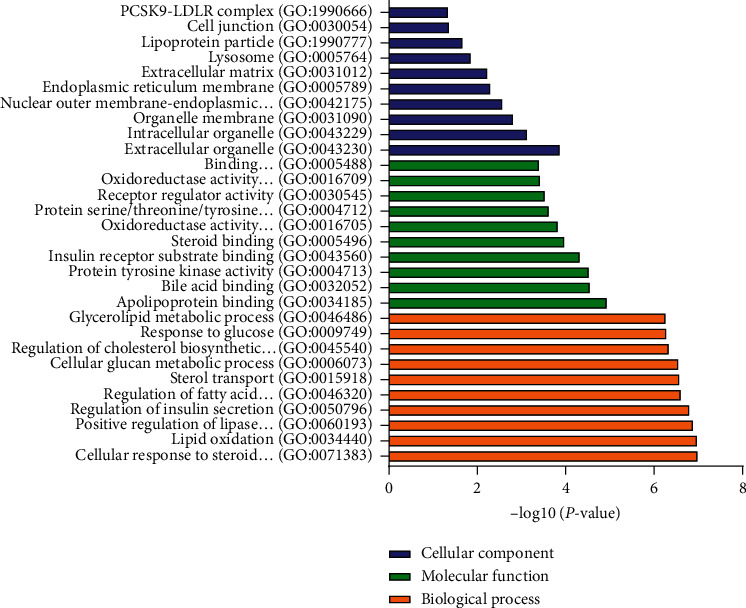
The GO functional analysis was processed by g:Profiler. 210 S-T were enriched in GO functional annotations (*P* < 0.05). Biological process (BP), molecular function (MF), and cellular components (CC) indicated the potential mechanism of treating hyperlipidemia with HTJZD.

HTJZD is composed of Alismatis Rhizoma (10 g), Poria (15 g), Atractylodis Macrocephalae Rhizoma (15 g), Atractylodis Rhizoma (10 g), Pinelliae Rhizoma Praeparatum (10 g), Citri Reticulatae Pericarpium (10 g), Glycyrrhizae Radix et Rhizoma (10 g), Ginseng Radix et Rhizoma (10 g), and Citri Reticulatae Pericarpium Viride (10 g). All medicines were the herbal decoction pieces (Figure 1(a)) purchased from Zisun Chinese Pharmaceutical Co., Ltd. (Guangzhou, China) and Yulin Chinaherborn Pharmaceutical Co., Ltd. (Guangxi, China). The mixture was soaked with pure water 10 times for 20 minutes, followed by boiling for 1 h and then adding pure water five times for 30-minute boiling. The medical herbal extraction was evaporated, concentrated to 2.52 g/mL, and then stored at 4°C prior to use.

### 2.7. Animals and Experimental Design

Forty male Sprague-Dawley (SD) rats (6-week-old, weight: 190 g ± 10 g) were purchased from Guangzhou Southern Medical University Laboratory Animal Science and Technology Development Co., Ltd., China (Certificate No. 44002100009585). All the animals were housed in the Laboratory Animal Center of Guangzhou University of Chinese Medicine (Guangzhou, China) under the standard and controlled conditions, 21–25°C temperature, 50–70% humidity, and 12-h light-dark cycle. HFD was obtained from Guangdong Medical Laboratory Animal Center, China. HFD recipe consists of 52.2% maintenance feed, 0.4% premix feed, 20% sucrose, 15% lard, 1.2% cholesterol, 0.2% sodium cholate, 10% casein, 0.6% calcium hydrophosphate, and 0.4% mountain flour.

This research design is based on a previous exploratory experimental model [23, 24]. After 1-week acclimatization, the 40 animals were randomly divided into the normal group (N, fed with chow diet, *n*= 10) and the HFD group (*n* = 30). The hyperlipidemia model establishment in way of feeding with HFD lasted for 30 days. Before collecting the blood samples, all the rats had to be fasted for 12 h. Serum lipids were measured using the kits to evaluate the establishment of hyperlipidemia model. In the intervention phase, the HFD group was continuously supplied with HFD but regrouped into three groups: model group (M, *n* = 10) administered saline, atorvastatin group (A, *n* = 10) administered 1.8 mg/kg per day of atorvastatin, and HTJZD group (H, *n* = 10) administered 12.6 g/kg per day of HTJZD. Each rat was given an intragastric dose of 1 mL/100g.

After 4 weeks of intervention, all animals were sacrificed for blood sample and liver sample collection. The blood samples were centrifuged at 3,500 rpm for 10 minutes to obtain the isolated serum and stored at −20°C immediately for subsequent biochemical testing. The liver tissues were stored at −80°C. The protocol was approved by the Animal Experimental Ethics Committee of Guangzhou University of Chinese Medicine.

### 2.8. Biochemical Analysis

After 30 days of feeding, blood samples were collected from the rats' posterior ocular venous plexus to judge the development of hyperlipidemia. Moreover, after 4 weeks of intervention, the animals were sacrificed for blood collection from abdominal aorta. Serum TC, TG, LDL-c, and HDL-c levels of each group were measured by detection kits (enzyme-labeling assay) according to the manufacture's operating instructions.

### 2.9. Evaluation Standard of HLP Model Establishment and Treatment

According to *The Evaluation Method of Auxiliary Hypolipidemic Function* [25] issued by Chinese State Food and Drug Administration, the mixed hyperlipidemia model establishment has to meet these requirements of lipid indexes: compared with those in the control group, serum TG, TC, or LDL-c levels have to be elevated significantly (*P* < 0.05) in the model group. As for the identification of treatment efficiency in the mixed hyperlipidemia model, conditions of the effective intervention wherein serum TC or LDL-c levels were significantly reduced (*P* < 0.05) were not accompanied by a significant increase in the HDL-c level or a decrease in the TG level in the intervention group when compared with the control group.

### 2.10. Histological Examinations

After dissection, liver specimens were fixed in 4% (w/v) paraformaldehyde for 24 h, followed by flushing with running water overnight. Moreover, tissues were dehydrated with graded ethanol and embedded in paraffin blocks. Thereafter, paraffin blocks were sectioned to 4 µm slices spread on the glass slides, which were then rehydrated and stained with HE. In addition, liver tissues were made into frozen samples. Fresh tissues were preserved in fixer for over 24 h, dehydrated with sucrose solution at 4°C, subsequently embedded on a molder with optimal cutting temperature freezing compound, and sectioned into 8 *μ*m slices for Oil Red O staining. All sections were observed by an upright optical microscope (Nikon Eclipse E100, Japan) and an image collection system (Nikon DS-US, Japan) at 200× magnification.

### 2.11. RNA Sequencing Analysis

Total RNA was extracted from liver tissue samples of three groups (N group, M group, and H group) by using TRIzol reagent. Moreover, agar gel electrophoresis and NanoDrop were applied to test the purity and qualify the concentration of RNA. Agilent 2100 Bioanalyzer was facilitated to examine RNA quality. The transcriptomic library of cDNA was constructed and verified by Qubit 3.0 and Agilent 2100. After the library was purified, the sequencer HiSeq 2500 (Illumina, USA) was used on all samples for paired-end sequencing. Clean reads were obtained according to the following filter criteria-eliminating low quality reads and reads that comprise adapters. Consequently, TopHat2 was applied in setting genome (Rnor_6.0) as sequencing alignment reference to clean reads used to attain the information from and beyond the referenced genome. Each gene expression was assessed by fragments per kilobase of transcript per million fragments mapped (FPKM). The computational method of FPKM is below:

(1) $$\begin{array}{c} \text{FPKM}=\frac{\text{cDNA Fragments}}{\text{Mapped Fragments}\left(\text{Millions}\right)\times \text{Transcript Length}\left(\text{kb}\right)}. \end{array}$$

DESeq was chosen for analyzing differential expression of genes in three groups. Screening criteria concerning a false discovery rate (FDR) of <0.05 and fold change (FC) of >1.5 or <0.7 were used to identify significant DEGs. Venn diagram was painted by Draw.io, and the heatmap was processed by pheatmap package of R Studio (version 4.0.2).

### 2.12. Screening Out the Common Genes between Regulated DEGs and S-T

Given that DEGs acquired from RNA-seq were based on *Rattus norvegicus* genomes, S-T from *H. Sapiens* have to be mapped to the orthology of rat in the HUGO Gene Nomenclature Committee (HGNC, https://www.genenames.org/) in order to search out the common genes between regulated DEGs and S-T.

### 2.13. Key Targets Validation by qRT-PCR Analysis

Total RNA was reversed to cDNA by Bestar qPCR RT kit (DBI Biosciences, Germany). Subsequently, each cDNA sample was used to quantify the expression of respective gene by SYBR Green qPCR Master Mix kit (DBI Biosciences, Germany). The 20 *μ*L reaction system containing cDNA, SYBR Green qPCR Master Mix, forward and reverse primers, and RNase-free ddH2O was placed into fluorescent qPCR analyzer Mx3000P (Agilent, USA), through a procedure of 40 cycles at 94°C for 2 min predegeneration, 94°C for 20 s degeneration, 58°C for 20 s renaturation, and 72°C for 20 s extension. Each sample was performed in triplicate with glyceraldehyde-3-phosphate dehydrogenase (GAPDH) as the reference. Gene primer sequences of Srebp-1c, Pcsk9, Insr, Gck, Cyp7a1, and Cyp3a9 used for reverse transcription polymerase chain reaction (PCR) are listed in Table 1. The data of qRT-PCR were analyzed by the 2^−ΔΔ*Ct*^ method to qualify the relative expression levels of candidate genes.

### 2.14. Statistical Analysis

All the data had passed the normal distribution test. Moreover, one-way analysis of variance was used to test if data met variance homogeneity. If so, the LSD test was used for multiple comparisons; if not, Welch's analysis of variance combined with the Games–Howell test was applied. All results are expressed as mean ± SD. *P*-values of<0.05 were considered statistically significant. All the steps were performed by using SPSS (IBM, USA, version 20.0).

## 3. Results

### 3.1. Identification of Compounds, C-T, D-T, I-T, and S-T

Based on the inclusive criteria of OB  ≥30% and DL ≥0.18, a total of 120 active compounds (Supplemental Table 1) were picked up from TCMSP. 1001 C-T (Supplemental Table 2) were collected from three databases (TCMSP, ETCM, Swiss Target Prediction) and standardized by UniProtKB. Additionally, 1297 D-T (Supplemental Table 2) were searched out from DisGeNET and GeneCards, with the name normalized. Furthermore, 282 I-T (Supplemental Table 2) were found in C-T and D-T after the intersection performed. Then, 282 I-T were put into STRING to determine the interaction relationship among the targets, and lastly 210 S-T (Supplemental Table 2) were identified according to the criteria of combined score of ≥0.95 in the node interaction index. Totally, 103 components (Supplemental Table 1) containing 210 S-T were left to construct the network.

### 3.2. GO Annotations and KEGG Pathway Enrichment Analysis of 210 S-T

**Figure 2:**
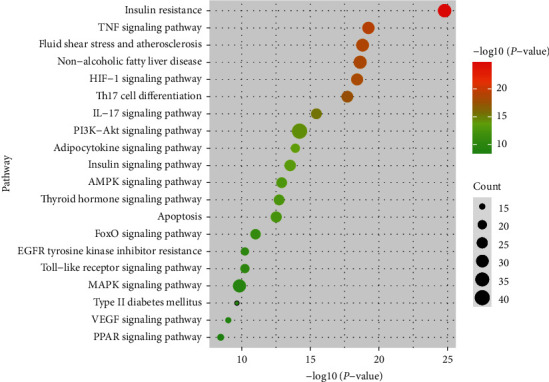
The KEGG pathway enrichment was processed by g:Profiler. 210 S-T were applied for KEGG pathway analysis (*P* < 0.01) to predict the critical pathways involved in the treatment of hyperlipidemia with HTJZD.

A total of 210 S-T, with strong connection among them, may play a key role for HTJZD in the treatment of hyperlipidemia. From BP annotations in the GO functional enrichment analysis (Figure 1) results (*P*< 0.05), the targets are mainly grouped into cellular response to steroid hormone stimulus, lipid oxidation, regulation of insulin secretion, regulation of fatty acid oxidation, and others. These targets were associated with apolipoprotein binding, bile acid binding, insulin receptor substrate binding, and others in MF annotation. In addition, it is predicted that HTJZD exerts a regulation effect on hyperlipidemia through the alteration of CC such as cellular organelle, organelle membrane, and lipoprotein particles. KEGG pathway enrichment analysis (*P*< 0.05) was performed as shown in Figure 2. The size and color of the bubble were determined by gene number and –log10(*P*-value), respectively. A total of 210 S-T were mainly enriched in the insulin resistance pathway, nonalcoholic fatty liver disease pathway, HIF-1 signaling pathway, PI3K-Akt signaling pathway, insulin signaling pathway, AMPK signaling pathway, etc.

We found that GO annotations and KEGG pathway enrichments showed a better view of the connections between targets and the underlying therapeutic mechanisms of HTJZD. From the enrichment results, lipid metabolism and insulin resistance processes presented a strong connection with the potential regulation effect of HTJZD in treating hyperlipidemia, which may shed light on the exploration of the efficacy of HTJZD.

### 3.3. Network Construction

**Figure 3:**
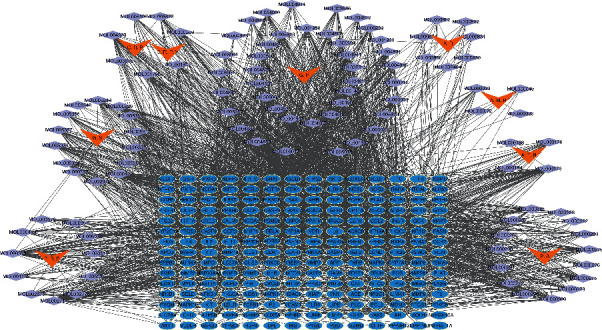
Herb-compound-S-T network was constructed to reveal the “multicompound, multitarget” mechanism of HTJZD. The network was composed of 9 herbs, 103 compounds, and 210 S-T. Orange nodes: herbs; purple nodes: compounds; blue nodes: S-T; P. A: Poria; A. R: Alismatis Rhizoma; A. M. R: Atractylodis Macrocephala Rhizoma; A. T. R: Atractylodis Rhizoma; P. R. P: Pinelliae Rhizoma Praeparatum; C. R. P: Citri Reticulatae Pericarpium; C. R. P. V: Citri Reticulatae Pericarpium Viride; G. S: Ginseng Radix et Rhizoma; G. R: Glycyrrhizae Radix et Rhizoma. For molecule names of compounds, refer to Supplemental Table 1 or look up the information on TCMSP.

The interaction network of herb-compound-S-T (Figure 3) was composed of 322 nodes and 2,207 edges. It is evident that herbs and compounds were strongly associated with the common targets between compounds and disease. In other words, HTJZD had an effect on hyperlipidemia through the regulation of these targets by major active ingredients. MOL000173 (wogonin), MOL004328 (naringenin), MOL000358 (beta-sitosterol), MOL0002714 (baicalein), and MOL000422 (kaempferol) are the top five nodes with high degree among the compounds in the network. Most of them have been identified as the effective ingredients to modulate the glucolipid metabolism.

**Figure 4:**
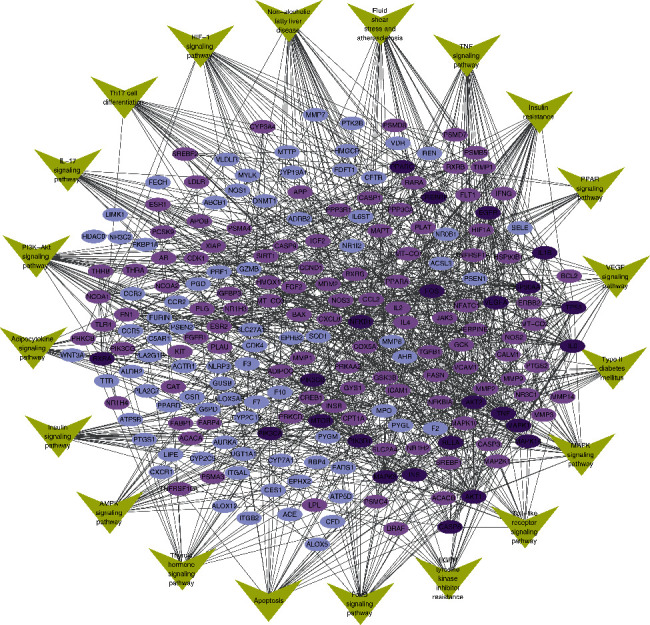
S-T–pathway network was constructed to present the “multipathway, multitarget” mechanism of HTJZD. It reveals the intersection of S-T and the relation between S-T and KEGG pathways. Green nodes represent the pathways, and nodes with gradient purple color are S-T. Depth of purple was determined by the degree value of nodes. The edge between the nodes was determined by the combined score analyzed by STRING.

The KEGG pathway enrichment results and interaction relation among 210 S-T from STRING provided original data to construct the S-T-pathway network (Figure 4). The network consisted of 230 nodes and 1,057 edges, in which color depth was determined by the degree of S-T nodes and edge was determined by the combined score. It is predicted that HTJZD acts on these potential targets on the pathways to be involved in the treatment of the disease. The insulin resistance pathway, PI3K-Akt signaling pathway, and nonalcoholic fatty liver disease are characterized by relatively high −log10(*P*-value) in Figure 2 and also by the relative high degree in the network, which may provide more possibility for the search of potential targets within the pathway.

### 3.4. Effect of HTJZD Treatment on Serum Lipid Levels, Body Weight, and Liver Index in HFD-Fed Rats

**Figure 5:**
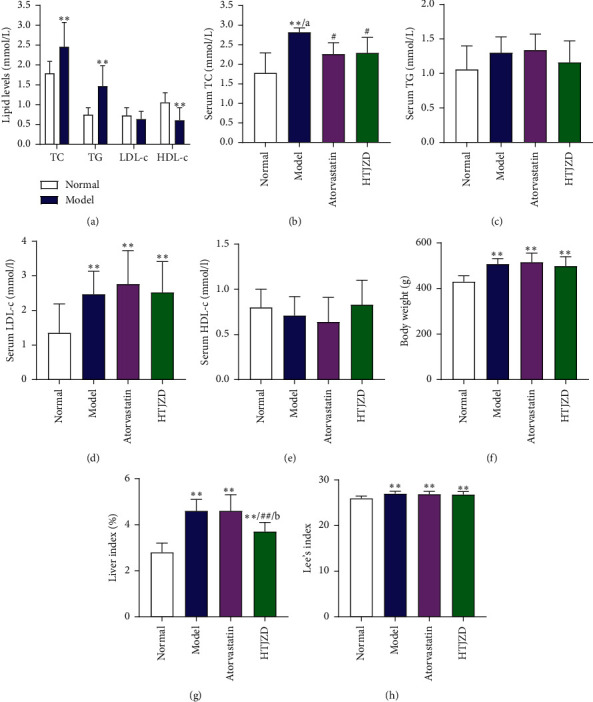
The rat model with hyperlipidemia was established by feeding HFD in male Sprague–Dawley rats after 30 days. (a) The lipid levels in rats after modeling time. After 4-week intervention, (b) serum TC, (c) serum TG, (d) serum LDL-c, (e) serum HDL-c level, (f) body weight, (g) liver index, and (h) Lee's index were analyzed to evaluate the hypolipidemic effect of HTJZD. Data were represented as the mean ± SD. ^*∗*^*P* < 0.05, ^*∗∗*^*P* < 0.01, compared with the normal group; ^#^*P* < 0.05, ^##^*P* < 0.01, compared with the model group; a: *P* < 0.05, b: *P* < 0.01, compared with the atorvastatin group.

**Figure 6:**
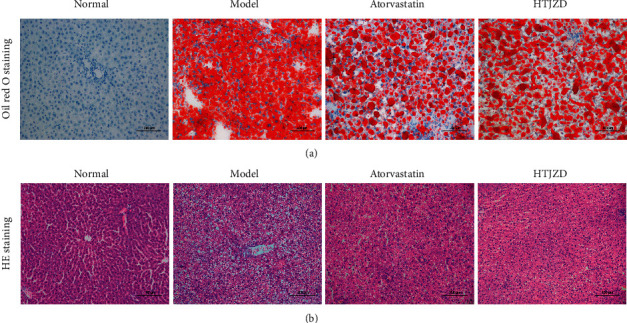
Liver samples were used for staining methods after 4-week intervention. (a) Oil Red O staining of liver tissue in each group (magnification, 200×). (b) HE staining of liver tissue in each group (magnification, 200×).

As shown in Figure 5, we observed the significant accumulation of serum TC and TG as well as the decrease in the HDL-c level in HFD-fed rats after 30 days, which indicated the development of hyperlipidemia in the HFD group (Figure 5(a)), according to *The Evaluation Method of Auxiliary Hypolipidemic Function* [25]. After 4 weeks of treatment, compared with the M group, the A and H groups remarkably reversed the growth of TC level (Figure 5(b), *P* < 0.05). Compared with atorvastatin, HTJZD was more advantageous in lowering TG and increasing HDL-c levels (Figures 5(c) and 5(e)). However, neither of two measures was statistically effective in LDL reduction, even if there exists a downward trend (Figure 5(d)). Therefore, HTJZD was determined as the medicine that positively reduces serum cholesterol. In addition, the liver index in the H group was significantly lower than that in the M and A groups (Figure 5(g)), which indicated the improvement of liver morphology and was later verified in pathological morphology examinations (Figures 6(a) and 6(b)). However, two treatments were not sensitive to reduce body weight and Lee's index (Figures 5(f) and 5(h)).

### 3.5. Effects of HTJZD Treatment on Hyperlipidemia-Related Pathological Process of Histology

The results of Oil Red O staining showed that lipid droplets were augmentative in the M group while a portion disappeared after atorvastatin and HTJZD intervention (Figure 6(a)). In addition, HE staining analysis showed that liver structure changed after HFD was supplemented in rats. Numerous lipid deposits in the normal liver cells induce the degeneration of liver and inflammatory cell infiltration in the portal area (Figure 6(b)). However, atorvastatin and HTJZD could obviously alleviate the damaged liver tissue and recover liver structure (Figure 6(b)). From the aforementioned results, HTJZD exerted lipid-lowering and liver protection effects similar to those of atorvastatin.

### 3.6. DEGs Regulated by HTJZD

**Figure 7:**
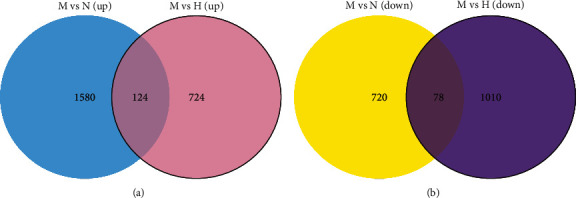
202 regulated DEGs. A Venn diagram showing the distribution of overlapped DEGs of model group when compared with normal group and HTJZD group, respectively. 124 were the common genes upregulated both in model vs normal (blue part) and model vs HTJZD (pink part) groups. 78 were the shared genes downregulated both in model vs normal (yellow part) and model vs HTJZD (purple part) groups.

**Figure 8:**
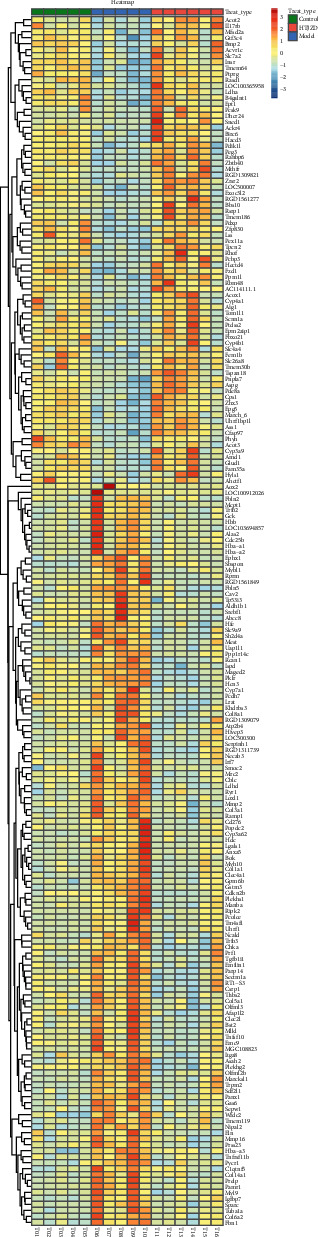
Hierarchical clustering heatmap of 202 regulated DEGs presenting different expression in liver samples of control group, model group, and HTZJD group, respectively. Samples are grouped by three modules on columns: T01-T05 within green module is control group; T06-T10 of blue module belongs to model group; T11-T16 clustered to pink module is HTJZD group. Expression of genes is represented on rows as a color lump in gradient color, transformed by FPKM and processed by pheatmap package in R Studio. Color scale is used to explain the variant expression of genes, with darker blue for lower values and brighter red for higher value.

This transcriptome study aimed to search out the potential targets, which were altered by hyperlipidemia and can be reversely regulated via the intervention of HTJZD, as the regulated DEGs. Based on the cutoff of FDR of <0.05 and FC >1.5 or <0.7, a total of 2,502 DEGs (1,704 upregulated; 798 downregulated) were screened out from M versus N, and 1,936 (848 upregulated; 1,088 downregulated) from M versus H. In addition, compared with the upregulation and downregulation parts, respectively, 202 (124 upregulated and 78 downregulated) were the regulated DEGs (Figure 7). Furthermore, the chosen 202 regulated DEGs are listed in Supplemental Table 3 and visualized as a heatmap in Figure 8. All the regulated DEGs were grouped by hierarchical clustering based on FPKM of each gene in each sample. We observed that the expression of these genes was significantly altered in the model group when compared to control group. Nevertheless, after the administration of HTJZD, the expression of these genes was recovered to normal level. Therefore, it can be seen that the therapeutic effect of HTJZD on HLP may be due to the regulation of these genes.

### 3.7. Common Genes between Regulated DEGs and S-T

**Figure 9:**
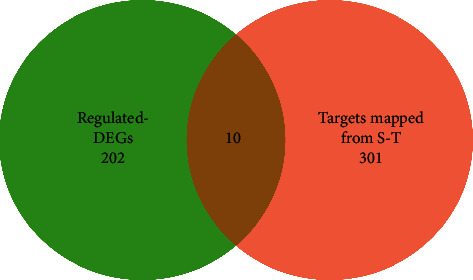
A Veen diagram showing 10 common targets between 202 regulated DEGs and 301 targets, mapped from the orthology of S-T in *Homo sapiens*.

A total of 210 S-T were mapped to 301 targets (Supplemental Table 4) of *R. norvegicus* genomes in the HGNC. Furthermore, 10 common targets (Figure 9), namely, Prf1, Cyp3a62, Srebf1, Pcsk9, Cyp7a1, Aldh1b1, Mmp2, Insr, Cyp3a9, and Gck, were found between 301 targets and 202 regulated DEGs. It is clear that 10 common targets (Table 2) presented an opposite trend in the model group and HTJZD group in RNA sequencing analysis, which may be attributed to validity and feasibility. Therefore, these common targets were put into the g:Profiler website for KEGG pathway enrichment. The result (Table 3) showed four key pathways (*P*< 0.05), namely, steroid hormone biosynthesis pathway, insulin signaling pathway, cholesterol metabolism pathway, and linoleic acid metabolism pathway, involved in HTJZD in treating rats with hyperlipidemia. Moreover, six key targets, namely, Srebf1, Cyp7a1, Cyp3a9, Pcsk9, Insr, and Gck, were selected from the results for further validation.

### 3.8. Validation of the Hypolipidemic Effects of HTJZD by qRT-PCR Analysis

**Figure 10:**
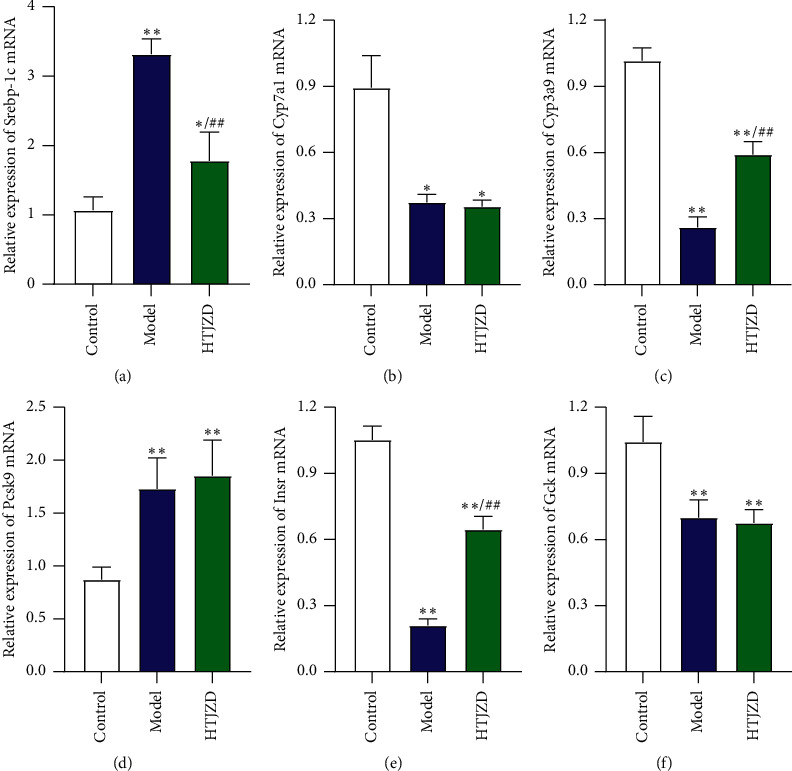
Validation of Srebp-1c (a), Cyp7a1 (b), Cyp3a9 (c), Pcsk9 (d), Insr (e), and Gck (f) mRNA expression levels in the liver tissues of rats by qRT-PCR analysis Data were expressed as mean ± SD. All the results were normalized to GAPDH mRNA expression. ^*∗*^*P* < 0.05, ^*∗∗*^*P* < 0.01, compared with the normal group; ^#^*P* < 0.05, ^##^*P* < 0.01, compared with the model group.

Six genes of Srebp-1c, Cyp7a1, Cyp3a9, Pcsk9, Insr, and Gck, closely associated with lipid metabolism and insulin signaling pathway, were selected for qRT-PCR analysis (Figure 10). Srebp-1c and Pcsk9 mRNA levels increased (*P* < 0.01) significantly in the M group compared with the N group, while the Cyp7a1, Cyp3a9, Insr, and Gck levels declined (*P*< 0.05), which indicated that HFD leads to the lipid–glucose metabolism disorders in SD rats. However, the mRNA expression in rats administered HTJZD showed a different picture. The expressions of Srebp-1c, Cyp3a9, and Insr mRNA were turning to a significant reversal (*P*< 0.01) in the H group when compared with the M group, which is consistent with the transcriptomic results, while Cyp7a1, Pcsk9, and Gck mRNA expression had no significant difference compared with rats with hyperlipidemia.

## 4. Discussion

Hyperlipidemia is a chronic disorder generally caused by a long-term unbalanced diet, which is manifested pathologically with aberrant lipid accumulation and energy metabolism disruption [26–28]. In addition, it is considered that lipid accumulation, especially ectopic fat deposition, leads to insulin resistance, impairing the lipolytic function of insulin, generating an amount of free fatty acids, and eventually accelerating the occurrence of metabolic diseases [29–31]. Therefore, attenuating hyperlipidemia via regulating lipid metabolism and insulin resistance could be effective solutions to reduce the biosynthesis of lipid and boost lipid consumption.

The results of active compounds collected from TCMSP indicated that HTJZD serves as a multicomponent prescription and especially contains the ingredients with lipid-lowering, glucose-reducing, and antioxidative effects. In the herb–compound–S-T network, wogonin, naringenin, beta-sitosterol, baicalein, and kaempferol are the potential compounds that may play a significant role in suppressing the progression of hyperlipidemia. Wogonin, screened out from the compounds of Atractylodis Rhizoma, has been reported to have an antioxidative effect and protect HFD-fed rats from developing nonalcoholic fatty liver disease [32]. Naringenin, found in three compounds (Citri Reticulatae Pericarpium, Glycyrrhizae Radix et Rhizoma, and Citri Reticulatae Pericarpium Viride) of HTJZD, inhibits the expression of lipogenic genes and improves hepatic steatosis in middle-aged Apoe ^−/−^ mice [33]. Kaempferol also could effectively reduce the lipid accumulation in mice and alleviate the insulin resistance in diabetic rats [34, 35].

The animal experimental results showed that HTJZD could reverse (*P* <  0.05) TC level (Figure 5(b)) and Liver index (Figure 5(g)) in HFD-fed rat model. In addition, HTJZD showed advantages over atorvastatin in TG reduction (Figure 5(c)) and HDL-c elevation (Figure 5(e)), but these were not statistically significant. We speculate that if the administration time is extended, the advantages of this decoction for restoring the normal serum lipids will be more prominent. To further disclose the roles of HTJZD in hyperlipidemia treatment, RNA sequencing technique was performed to investigate the changes of RNA sequence in rat liver. This study found that the expressions of 202 genes were conversely altered after the intervention of HTJZD, which showed that the intervention strategy had an impact on the disease.

Furthermore, the GO and KEGG analysis of S-T, S-T–pathway network, and KEGG pathway enrichment of 10 common genes between regulated DEGs and S-T all indicated that HTJZD exerted therapeutic action on hyperlipidemia by modulating lipid metabolism and insulin resistance. Lipid metabolism regulation should be considered as the pivotal function of HTJZD. With the transcript variants of Srebp-1a and Srebp-1c, Srebp1 (also known as Srebf1) is significant transcription factor involved in regulating liposomal homeostasis.

It transcriptionally regulates the expression of its downstream lipogenic genes involving in the synthesis of fatty acid and triglyceride [36, 37]. It is noted that the activation of AMPK could inhibit the activation of Srebp1 and acetyl-CoA carboxylase 1 (Acc1), leading to the reduction of lipid biosynthesis [38]. Cytochrome P450, family three, subfamily A (Cyp3a), is involved in the biological transformation of endogenous substrates and mainly mediates drug metabolism [39]. Moreover, when liver damage by HFD occurs, the expression and bioactivity of Cyp3a are impaired [40]. HTJZD could recover the expression of cytochrome P450, family three, subfamily A, polypeptide 9 (Cyp3a9), which is decreased by HFD and functions in two significant signaling pathways, that is, steroid hormone biosynthesis and linoleic acid metabolism, when HTJZD treats hyperlipidemia. In addition, proprotein convertase subtilisin/kexin type 9 (Pcsk9) and cholesterol 7*α*-hydroxylase (Cyp7a1) were the key genes both enriched in the cholesterol metabolism pathways in hyperlipidemia rats treated with HTJZD. Evidence from genetic studies supports the role of Pcsk9, a serine protease, in the regulation of hepatic apolipoprotein B uptake and degradation of low-density lipoprotein receptor (LDLR) [41–43]. Moreover, Pcsk9 is another downstream gene of Srebp-1c and is regulated by it, serving as a significant gene to promote cholesterol synthesis. Cyp7a1 is the rate-limiting enzyme, which is pivotal in the process of de novo bile acid synthesis [44, 45]. In addition, the activation of Cyp7a1 could inhibit the function of Srebp-1c so as to inhibit the lipid accumulation [46, 47]. However, our transcriptomic results of this study about the expressions of Cyp7a1 and Pcsk9 were not in accordance with the reported ones. Nevertheless, some pertinent literature reported that the activation of Farnesoid X receptor (FXR, bile acid receptor) could induce the SHP so as to inhibit the expression of Cyp7a1 in the transcriptomic level [48, 49]. We speculated that HFD-fed rats had developed the symptoms of fatty liver and disorder of bile acid and consequently HTJZD could regulate bile acid metabolism by activating FXR.

Insulin resistance, regarded as another representative pathological feature of metabolic disorders, aggravating on account of the inhibition of the insulin receptor substrates (Irs) pathway, could also be regulated by AMPK signaling pathway [50]. Additionally, the initiation of insulin resistance could decrease AMPK phosphorylation, further increase the expression of Srebp-1c, and eventually suppress the Irs-1-related insulin signaling pathway [51]. The transcriptomic results showed the upregulation of Insr and downregulation of Srebp-1c in HTJZD-fed rats, indicating that HTJZD can activate the insulin signaling pathway while suppressing the insulin resistance pathway. Glucokinase (Gck) is a key enzyme in the regulation of glucose metabolism and insulin secretion. It is also the first rate-limiting enzyme in the glycolysis pathway. It catalyzes glucose phosphorylation and is a prerequisite for liver glycogen synthesis. Gck translocation mainly mediated whole-body glucose homeostasis, even the insulin-stimulated hepatic glycogen synthesis, which means that the activation of Gck could accelerate the synthesis process of hepatic glycogen and alleviate dysglycemia and dyslipidemia in liver [52]. However, our RNA sequencing results showed that Gck had not been improved by HTJZD, which indicated that HTJZD affects glucose metabolism in rats not through Gck associated pathways but mainly through Srebp-1c/Insr pathway.

Taken together, HTJZD acted as a hypolipidemic agent potentially via modulation of lipid metabolism and insulin resistance. An increasing number of studies report that the lipid metabolism-related signaling pathway and insulin signaling pathway could be regulated by natural compounds, which indicates that traditional herbal medicines are potential alternatives or supplements to treat metabolic diseases [53–55]. In addition, KEGG pathway enrichment results of 210 S-T also predict that HTJZD not only can treat hyperlipidemia, but also has the potential to cure other relevant diseases, such as type 2 II diabetes, nonalcoholic fatty liver disease, and obesity. Therefore, this study demonstrated the rationality of the principle of CM—treating different diseases with the same method—at the molecular mechanism level. It also reveals that invigorating the spleen and regulating qi, as well as reducing phlegm and removing turbidity, may be the scientific method of treating metabolic diseases in modern times.

## 5. Conclusion

Network pharmacology predicted that HTJZD may exert therapeutic effects on HLP. Moreover, the animal experiments verified that HTJZD effectively suppressed the pathological process of rat model fed with HFD. It is capable of regulating serum lipids of rats, reducing lipid deposition in rat's liver, and reversing the expression of genes which related to lipid metabolism and insulin resistence. Among six candidate targets (Srebp-1c, Cyp7a1, Cyp3a9, Pcsk9, Insr, and Gck), Srebp-1c and Insr may play central role in the treatment of hyperlipidemia.

### 5.1. Limitations

There are several limitations to the present study. Repeated experiments and prolonged intervention duration are needed in future studies. What has to be focused is whether the compensation mechanism, leading to the opposite expression of genes contrary to what was expected, exists in the liver sites with more severe lipid lesion. Furthermore, a blood glucose level test should be performed to further support the intervention effect on insulin resistance of HTJZD. We will pursue a more systematic comprehensive analysis of HTJZD intervening hyperlipidemia and relevant metabolic disorders in future study.

## Figures and Tables

**Table 1:** Primer sequences used for qRT-PCR.

Gene name	Forward primer (5′-3′)	Reverse primer (5′- 3′)
GAPDH	CCTCGTCTCATAGACAAGATGGT	GGGTAGAGTCATACTGGAACATG
Srebp-1c	GCAAGTACACAGGAGGCCAT	AGATCTCTGCCAGTGTTGCC
Cyp7a1	GGAAGACTCTTTGCCGTCCA	CAAAATTCCCAAGCCTGCCC
Cyp3a9	AGAATTCGAGCCCTGCTGTC	CCGATCCCTGCCTCATGTTT
Pcsk9	CAAGGACTGGGGTAGTGCTG	CTCCGATGATGTCCTTCCCG
Insr	GCTACCTGGCCACTATCGAC	AACTGCCCATTGATGACGGT
Gck	CTGCTTAAGCTGGTGGACGA	GAAGCCCCAGAGTGCTTAGG

**Table 2:** 10 common genes between regulated DEGs (HTJZD versus model) and S-T in RNA sequencing analysis.

Gene symbol	Fold change	FDR
	HTJZD vs model	HTJZD vs model
Upregulated		
Pcsk9	3.606	0
Cyp3a9	2.935	0.007
Insr	1.682	0.012
Downregulated		
Srebp-1c	0.248	0
Gck	0.257	0
Cyp7a1	0.312	0.003
Aldh1b1	0.319	0.002
Prf1	0.488	0.04
Cyp3a62	0.497	0.014
Mmp2	0.519	0.008

**Table 3:** KEGG signaling pathways enrichment (*P* < 0.05) of 10 common genes.

Pathway ID	Pathway	Gene	*P*-value
KEGG:00140	Steroid hormone biosynthesis	Cyp3a62, Cyp7a1, Cyp3a9	0.001511
KEGG:04910	Insulin signaling pathway	Srebf1, Insr, Gck	0.017021
KEGG:04979	Cholesterol metabolism	Pcsk9, Cyp7a1	0.019844
KEGG:00591	Linoleic acid metabolism	Cyp3a62, Cyp3a9	0.030053

## Data Availability

The data used to support the results of this study are included within the article and the supplementary materials.

## Supplementary Materials

Supplementary Materials Supplemental Table 1: 120 compounds of herbs in HTJZD meeting the criteria of OB ≥ 30% and DL ≥ 0.18 were picked up from TCMSP. Supplemental Table 2: 1001 compound-related targets (C-T) screened out from TCMSP, ETCM, and Swiss Target Prediction and 1297 disease-related targets (D-T) collected from DisGeNET and GeneCards. Supplemental Table 3: 202 regulated differentially expressed genes from RNA-seq analysis results. Supplemental Table 4: 301 targets of rat genomes mapped from the orthology of selected-targets (S-T) in human sapiens were obtained from HGNC. The supplemental materials are accessible to the interested readers on the website of Evidence-Based Complementary and Alternative Medicine.
