# Supplementary material for: Based on Network Pharmacology and RNA Sequencing Techniques to Explore the Molecular Mechanism of Huatan Jiangzhuo Decoction for Treating Hyperlipidemia
Source: Evid Based Complement Alternat Med. 2021 Apr 9;2021:9863714. doi: 10.1155/2021/9863714 (PMC8055390; doi:10.1155/2021/9863714)
Supplement: Supplementary Materials — Supplemental Table 1: 120 compounds of herbs in HTJZD meeting the criteria of OB ≥ 30% and DL ≥ 0.18 were picked up from TCMSP. Supplemental Table 2: 1001 compound-related targets (C-T) screened out from TCMSP, ETCM, and Swiss Target Prediction and 1297 disease-related targets (D-T) collected from DisGeNET and GeneCards. Supplemental Table 3: 202 regulated differentially expressed genes from RNA-seq analysis results. Supplemental Table 4: 301 targets of rat genomes mapped from the orthology of selected-targets (S-T) in human sapiens were obtained from HGNC. The supplemental materials are accessible to the interested readers on the website of Evidence-Based Complementary and Alternative Medicine. [file 9863714.f1.zip › 9863714.f1/9863714_Supplemental table 1 Xiaowen Zhou .docx]

**Supplemental table 1.** 120 compounds of herbs in HTJZD that meeting the criteria of OB of $\geq$ 30% and DL of $\geq$ 0.18 were picked up from TCMSP. Number 1 to 103 are the compounds that containing the intersection targets between compound-related targets and disease-related targets, moreover, meeting the criteria of combined score of $\geq$ 0.95 between the targets.

| Number | Mol ID | Molecule Name | OB (%) | | DL |
| --- | --- | --- | --- | --- | --- |
| 1 | MOL000273 | (2R)-2-[(3S,5R,10S,13R,14R,16R,17R)-3,16-dihydroxy-4,4,10,13,14-pentamethyl-2,3,5,6,12,15,16,17-octahydro-1H-cyclopenta[a]phenanthren-17-yl]-6-methylhept-5-enoic acid | | 30.93 | 0.81 |
| 2 | MOL000275 | trametenolic acid | | 38.71 | 0.8 |
| 3 | MOL000276 | 7,9(11)-dehydropachymic acid | | 35.11 | 0.81 |
| 4 | MOL000279 | Cerevisterol | | 37.96 | 0.77 |
| 5 | MOL000280 | (2R)-2-[(3S,5R,10S,13R,14R,16R,17R)-3,16-dihydroxy-4,4,10,13,14-pentamethyl-2,3,5,6,12,15,16,17-octahydro-1H-cyclopenta[a]phenanthren-17-yl]-5-isopropyl-hex-5-enoic acid | | 31.07 | 0.82 |
| 6 | MOL000282 | ergosta-7,22E-dien-3beta-ol | | 43.51 | 0.72 |
| 7 | MOL000283 | Ergosterol peroxide | | 40.36 | 0.81 |
| 8 | MOL000285 | (2R)-2-[(5R,10S,13R,14R,16R,17R)-16-hydroxy-3-keto-4,4,10,13,14-pentamethyl-1,2,5,6,12,15,16,17-octahydrocyclopenta[a]phenanthren-17-yl]-5-isopropyl-hex-5-enoic acid | | 38.26 | 0.82 |
| 9 | MOL000287 | 3beta-Hydroxy-24-methylene-8-lanostene-21-oic acid | | 38.7 | 0.81 |
| 10 | MOL000289 | pachymic acid | | 33.63 | 0.81 |
| 11 | MOL000290 | Poricoic acid A | | 30.61 | 0.76 |
| 12 | MOL000291 | Poricoic acid B | | 30.52 | 0.75 |
| 13 | MOL000292 | poricoic acid C | | 38.15 | 0.75 |
| 14 | MOL000296 | hederagenin | | 36.91 | 0.75 |
| 15 | MOL000300 | dehydroeburicoic acid | | 44.17 | 0.83 |
| 16 | MOL000359 | sitosterol | | 36.91 | 0.75 |
| 17 | MOL002464 | 1-Monolinolein | | 37.18 | 0.3 |
| 18 | MOL000831 | Alisol B monoacetate | | 35.58 | 0.81 |
| 19 | MOL000830 | Alisol B | | 34.47 | 0.82 |
| 20 | MOL000832 | alisol, b, 23-acetate | | 32.52 | 0.82 |
| 21 | MOL000862 | [(1S,3R)-1-[(2R)-3,3-dimethyloxiran-2-yl]-3-[(5R,8S,9S,10S,11S,14R)-11-hydroxy-4,4,8,10,14-pentamethyl-3-oxo-1,2,5,6,7,9,11,12,15,16-decahydrocyclopenta[a]phenanthren-17-yl] butyl] acetate | | 35.58 | 0.81 |
| 22 | MOL000854 | alisol C | | 32.7 | 0.82 |
| 23 | MOL000856 | alisol C monoacetate | | 33.06 | 0.83 |
| 24 | MOL000033 | (3S,8S,9S,10R,13R,14S,17R)-10,13-dimethyl-17-[(2R,5S)-5-propan-2-yloctan-2-yl]-2,3,4,7,8,9,11,12,14,15,16,17-dodecahydro-1H-cyclopenta[a]phenanthren-3-ol | | 36.23 | 0.78 |
| 25 | MOL000022 | 14-acetyl-12-senecioyl-2E,8Z,10E-atractylentriol | | 63.37 | 0.3 |
| 26 | MOL000049 | 3β-acetoxyatractylone | | 54.07 | 0.22 |
| 27 | MOL000072 | 8β-ethoxy atractylenolide Ⅲ | | 35.95 | 0.21 |
| 28 | MOL000173 | wogonin | | 30.68 | 0.23 |
| 29 | MOL000179 | 2-Hydroxyisoxypropyl-3-hydroxy-7-isopentene-2,3-dihydrobenzofuran-5-carboxylic | | 45.2 | 0.2 |
| 30 | MOL000184 | NSC63551 | | 39.25 | 0.76 |
| 31 | MOL000188 | 3β-acetoxyatractylone | | 40.57 | 0.22 |
| 32 | MOL004328 | naringenin | | 59.29 | 0.21 |
| 33 | MOL005100 | 5,7-dihydroxy-2-(3-hydroxy-4-methoxyphenyl) chroman-4-one | | 47.74 | 0.27 |
| 34 | MOL005815 | Citromitin | | 86.9 | 0.51 |
| 35 | MOL005828 | nobiletin | | 61.67 | 0.52 |
| 36 | MOL001755 | 24-Ethylcholest-4-en-3-one | | 36.08 | 0.76 |
| 37 | MOL002670 | Cavidine | | 35.64 | 0.81 |
| 38 | MOL002714 | baicalein | | 33.52 | 0.21 |
| 39 | MOL002776 | Baicalin | | 40.12 | 0.75 |
| 40 | MOL000358 | beta-sitosterol | | 36.91 | 0.75 |
| 41 | MOL000449 | Stigmasterol | | 43.83 | 0.76 |
| 42 | MOL005030 | gondoic acid | | 30.7 | 0.2 |
| 43 | MOL000519 | coniferin | | 31.11 | 0.32 |
| 44 | MOL006936 | 10,13-eicosadienoic | | 39.99 | 0.2 |
| 45 | MOL006957 | (3S,6S)-3-(benzyl)-6-(4-hydroxybenzyl) piperazine-2,5-quinone | | 46.89 | 0.27 |
| 46 | MOL003578 | Cycloartenol | | 38.69 | 0.78 |
| 47 | MOL001484 | Inermine | | 75.18 | 0.54 |
| 48 | MOL000211 | Mairin | | 55.38 | 0.78 |
| 49 | MOL002311 | Glycyrol | | 90.78 | 0.67 |
| 50 | MOL000239 | Jaranol | | 50.83 | 0.29 |
| 51 | MOL003656 | Lupiwighteone | | 51.64 | 0.37 |
| 52 | MOL000392 | formononetin | | 69.67 | 0.21 |
| 53 | MOL004808 | glyasperin B | | 65.22 | 0.44 |
| 54 | MOL004810 | glyasperin F | | 75.84 | 0.54 |
| 55 | MOL004820 | kanzonols W | | 50.48 | 0.52 |
| 56 | MOL004824 | (2S)-6-(2,4-dihydroxyphenyl)-2-(2-hydroxypropan-2-yl)-4-methoxy-2,3-dihydrofuro[3,2-g] chromen-7-one | | 60.25 | 0.63 |
| 57 | MOL004829 | Glepidotin B | | 64.46 | 0.34 |
| 58 | MOL004835 | Glypallichalcone | | 61.6 | 0.19 |
| 59 | MOL004838 | 8-(6-hydroxy-2-benzofuranyl)-2,2-dimethyl-5-chromenol | | 58.44 | 0.38 |
| 60 | MOL004841 | Licochalcone B | | 76.76 | 0.19 |
| 61 | MOL004849 | 3-(2,4-dihydroxyphenyl)-8-(1,1-dimethylprop-2-enyl)-7-hydroxy-5-methoxy-coumarin | | 59.62 | 0.43 |
| 62 | MOL004855 | Licoricone | | 63.58 | 0.47 |
| 63 | MOL004856 | Gancaonin A | | 51.08 | 0.4 |
| 64 | MOL004863 | 3-(3,4-dihydroxyphenyl)-5,7-dihydroxy-8-(3-methylbut-2-enyl) chromone | | 66.37 | 0.41 |
| 65 | MOL004879 | Glycyrin | | 52.61 | 0.47 |
| 66 | MOL004885 | licoisoflavanone | | 52.47 | 0.54 |
| 67 | MOL004891 | shinpterocarpin | | 80.3 | 0.73 |
| 68 | MOL004903 | liquiritin | | 65.69 | 0.74 |
| 69 | MOL004904 | licopyranocoumarin | | 80.36 | 0.65 |
| 70 | MOL004907 | Glyzaglabrin | | 61.07 | 0.35 |
| 71 | MOL004908 | Glabridin | | 53.25 | 0.47 |
| 72 | MOL004910 | Glabranin | | 52.9 | 0.31 |
| 73 | MOL004912 | Glabrone | | 52.51 | 0.5 |
| 74 | MOL004914 | 1,3-dihydroxy-8,9-dimethoxy-6-benzofurano[3,2-c]chromenone | | 62.9 | 0.53 |
| 75 | MOL004941 | (2R)-7-hydroxy-2-(4-hydroxyphenyl)chroman-4-one | | 71.12 | 0.18 |
| 76 | MOL004959 | 1-Methoxyphaseollidin | | 69.98 | 0.64 |
| 77 | MOL004990 | 7,2',4'-trihydroxy－5-methoxy-3－arylcoumarin | | 83.71 | 0.27 |
| 78 | MOL004993 | 8-prenylated eriodictyol | | 53.79 | 0.4 |
| 79 | MOL000500 | Vestitol | | 74.66 | 0.21 |
| 80 | MOL005000 | Gancaonin G | | 60.44 | 0.39 |
| 81 | MOL005001 | Gancaonin H | | 50.1 | 0.78 |
| 82 | MOL005003 | Licoagrocarpin | | 58.81 | 0.58 |
| 83 | MOL005007 | Glyasperins M | | 72.67 | 0.59 |
| 84 | MOL005012 | Licoagroisoflavone | | 57.28 | 0.49 |
| 85 | MOL005017 | Phaseol | | 78.77 | 0.58 |
| 86 | MOL005018 | Xambioona | | 54.85 | 0.87 |
| 87 | MOL005020 | dehydroglyasperins C | | 53.82 | 0.37 |
| 88 | MOL001798 | neohesperidin_qt | | 71.17 | 0.27 |
| 89 | MOL001803 | Sinensetin | | 50.56 | 0.45 |
| 90 | MOL002879 | Diop | | 43.59 | 0.39 |
| 91 | MOL000422 | kaempferol | | 41.88 | 0.24 |
| 92 | MOL005308 | Aposiopolamine | | 66.65 | 0.22 |
| 93 | MOL005314 | Celabenzine | | 101.88 | 0.49 |
| 94 | MOL005317 | Deoxyharringtonine | | 39.27 | 0.81 |
| 95 | MOL005318 | Dianthramine | | 40.45 | 0.2 |
| 96 | MOL005320 | arachidonate | | 45.57 | 0.2 |
| 97 | MOL005344 | ginsenoside rh2 | | 36.32 | 0.56 |
| 98 | MOL005348 | Ginsenoside-Rh4_qt | | 31.11 | 0.78 |
| 99 | MOL005356 | Girinimbin | | 61.22 | 0.31 |
| 100 | MOL005357 | Gomisin B | | 31.99 | 0.83 |
| 101 | MOL005376 | Panaxadiol | | 33.09 | 0.79 |
| 102 | MOL005384 | suchilactone | | 57.52 | 0.56 |
| 103 | MOL000787 | Fumarine | | 59.26 | 0.83 |
| 104 | MOL000853 | alisol B | | 36.76 | 0.82 |
| 105 | MOL000849 | 16β-methoxyalisol B monoacetate | | 32.43 | 0.77 |
| 106 | MOL000020 | 12-senecioyl-2E,8E,10E-atractylentriol | | 62.4 | 0.22 |
| 107 | MOL000021 | 14-acetyl-12-senecioyl-2E,8E,10E-atractylentriol | | 60.31 | 0.31 |
| 108 | MOL000028 | α-Amyrin | | 39.51 | 0.76 |
| 109 | MOL000186 | Stigmasterol 3-O-beta-D-glucopyranoside_qt | | 43.83 | 0.76 |
| 110 | MOL000085 | beta-daucosterol_qt | | 36.91 | 0.75 |
| 111 | MOL000088 | beta-sitosterol 3-O-glucoside_qt | | 36.91 | 0.75 |
| 112 | MOL000092 | daucosterin_qt | | 36.91 | 0.76 |
| 113 | MOL000094 | daucosterol_qt | | 36.91 | 0.76 |
| 114 | MOL006937 | 12,13-epoxy-9-hydroxynonadeca-7,10-dienoic acid | | 42.15 | 0.24 |
| 115 | MOL003648 | Inermin | | 65.83 | 0.54 |
| 116 | MOL004492 | Chrysanthemaxanthin | | 38.72 | 0.58 |
| 117 | MOL005321 | Frutinone A | | 65.9 | 0.34 |
| 118 | MOL005360 | malkangunin | | 57.71 | 0.63 |
| 119 | MOL005399 | alexandrin_qt | | 36.91 | 0.75 |
| 120 | MOL005401 | ginsenoside Rg5_qt | | 39.56 | 0.79 |
