# Supplementary material for: Based on Network Pharmacology and RNA Sequencing Techniques to Explore the Molecular Mechanism of Huatan Jiangzhuo Decoction for Treating Hyperlipidemia
Source: Evid Based Complement Alternat Med. 2021 Apr 9;2021:9863714. doi: 10.1155/2021/9863714 (PMC8055390; doi:10.1155/2021/9863714)
Supplement: Supplementary Materials — Supplemental Table 1: 120 compounds of herbs in HTJZD meeting the criteria of OB ≥ 30% and DL ≥ 0.18 were picked up from TCMSP. Supplemental Table 2: 1001 compound-related targets (C-T) screened out from TCMSP, ETCM, and Swiss Target Prediction and 1297 disease-related targets (D-T) collected from DisGeNET and GeneCards. Supplemental Table 3: 202 regulated differentially expressed genes from RNA-seq analysis results. Supplemental Table 4: 301 targets of rat genomes mapped from the orthology of selected-targets (S-T) in human sapiens were obtained from HGNC. The supplemental materials are accessible to the interested readers on the website of Evidence-Based Complementary and Alternative Medicine. [file 9863714.f1.zip › 9863714.f1/9863714_Supplemental table 2 Xiaowen Zhou.docx]

**Supplemental table 2.** A total of 1001 Compound-related targets (C-T) screened out from TCMSP, ETCM, and Swiss Target Prediction and 1297 Disease-related targets (D-T) collected from DisGeNET and GeneCards. Number 1-282 are the intersection targets (I-T) between C-T and D-T, number 1- 210 are the selected targets (S-T) which meeting the criteria of combined score of $\geq$ 0.95.

| Number | C-T | Uniprot ID | D-T | Uniprot ID |
| --- | --- | --- | --- | --- |
| 1 | LPL | P06858 | LPL | P06858 |
| 2 | APOB | P04114 | APOB | P04114 |
| 3 | LDLR | P01130 | LDLR | P01130 |
| 4 | LIPE | Q05469 | LIPE | Q05469 |
| 5 | INS | P01308 | INS | P01308 |
| 6 | HMGCR | P04035 | HMGCR | P04035 |
| 7 | ADIPOQ | Q15848 | ADIPOQ | Q15848 |
| 8 | PPARA | Q07869 | PPARA | Q07869 |
| 9 | PCSK9 | Q8NBP7 | PCSK9 | Q8NBP7 |
| 10 | ATP5B | P06576 | ATP5B | P06576 |
| 11 | PPARG | P37231 | PPARG | P37231 |
| 12 | ACE | P12821 | ACE | P12821 |
| 13 | SERPINE1 | P05121 | SERPINE1 | P05121 |
| 14 | RXRG | P48443 | RXRG | P48443 |
| 15 | MTTP | P55157 | MTTP | P55157 |
| 16 | IL6 | P05231 | IL6 | P05231 |
| 17 | SREBF2 | Q12772 | SREBF2 | Q12772 |
| 18 | TNF | P01375 | TNF | P01375 |
| 19 | SREBF1 | P36956 | SREBF1 | P36956 |
| 20 | NR1H4 | Q96RI1 | NR1H4 | Q96RI1 |
| 21 | F2 | P00734 | F2 | P00734 |
| 22 | PYGL | P06737 | PYGL | P06737 |
| 23 | MMP9 | P14780 | MMP9 | P14780 |
| 24 | AKT2 | P31751 | AKT2 | P31751 |
| 25 | NOS3 | P29474 | NOS3 | P29474 |
| 26 | PLA2G7 | Q13093 | PLA2G7 | Q13093 |
| 27 | CPT1A | P50416 | CPT1A | P50416 |
| 28 | EPHX2 | P34913 | EPHX2 | P34913 |
| 29 | FASN | P49327 | FASN | P49327 |
| 30 | F3 | P13726 | F3 | P13726 |
| 31 | CCL2 | P13500 | CCL2 | P13500 |
| 32 | ESR1 | P03372 | ESR1 | P03372 |
| 33 | CYP7A1 | P22680 | CYP7A1 | P22680 |
| 34 | VCAM1 | P19320 | VCAM1 | P19320 |
| 35 | CYP19A1 | P11511 | CYP19A1 | P11511 |
| 36 | ICAM1 | P05362 | ICAM1 | P05362 |
| 37 | NCOA1 | Q15788 | NCOA1 | Q15788 |
| 38 | AGTR1 | P30556 | AGTR1 | P30556 |
| 39 | VEGFA | P15692 | VEGFA | P15692 |
| 40 | NCOA2 | Q15596 | NCOA2 | Q15596 |
| 41 | IGFBP3 | P17936 | IGFBP3 | P17936 |
| 42 | SELE | P16581 | SELE | P16581 |
| 43 | CES1 | P23141 | CES1 | P23141 |
| 44 | MPO | P05164 | MPO | P05164 |
| 45 | TIMP1 | P01033 | TIMP1 | P01033 |
| 46 | EGFR | P00533 | EGFR | P00533 |
| 47 | CXCL8 | P10145 | CXCL8 | P10145 |
| 48 | PLAT | P00750 | PLAT | P00750 |
| 49 | GZMB | P10144 | GZMB | P10144 |
| 50 | TTR | P02766 | TTR | P02766 |
| 51 | ABCB1 | P08183 | ABCB1 | P08183 |
| 52 | TLR4 | O00206 | TLR4 | O00206 |
| 53 | NR0B1 | P51843 | NR0B1 | P51843 |
| 54 | ADRB2 | P07550 | ADRB2 | P07550 |
| 55 | FABP4 | P15090 | FABP4 | P15090 |
| 56 | IL1B | P01584 | IL1B | P01584 |
| 57 | NR3C1 | P04150 | NR3C1 | P04150 |
| 58 | AR | P10275 | AR | P10275 |
| 59 | ALOX5AP | P20292 | ALOX5AP | P20292 |
| 60 | NR1H2 | P55055 | NR1H2 | P55055 |
| 61 | FECH | P22830 | FECH | P22830 |
| 62 | PRF1 | P14222 | PRF1 | P14222 |
| 63 | REN | P00797 | REN | P00797 |
| 64 | PSMD7 | P51665 | PSMD7 | P51665 |
| 65 | CCR3 | P51677 | CCR3 | P51677 |
| 66 | IGF2 | P01344 | IGF2 | P01344 |
| 67 | PYGM | P11217 | PYGM | P11217 |
| 68 | CXCR1 | P25024 | CXCR1 | P25024 |
| 69 | CYP3A4 | P08684 | CYP3A4 | P08684 |
| 70 | HMOX1 | P09601 | HMOX1 | P09601 |
| 71 | PRKAA2 | P54646 | PRKAA2 | P54646 |
| 72 | NR1H3 | Q13133 | NR1H3 | Q13133 |
| 73 | ALOX5 | P09917 | ALOX5 | P09917 |
| 74 | MTOR | P42345 | MTOR | P42345 |
| 75 | FDFT1 | P37268 | FDFT1 | P37268 |
| 76 | FN1 | P02751 | FN1 | P02751 |
| 77 | CAT | P04040 | CAT | P04040 |
| 78 | FGFR1 | P11362 | FGFR1 | P11362 |
| 79 | CDK4 | P11802 | CDK4 | P11802 |
| 80 | BRAF | P15056 | BRAF | P15056 |
| 81 | KIT | P10721 | KIT | P10721 |
| 82 | MDM2 | Q00987 | MDM2 | Q00987 |
| 83 | PIK3R1 | P27986 | PIK3R1 | P27986 |
| 84 | GUSB | P08236 | GUSB | P08236 |
| 85 | C5AR1 | P21730 | C5AR1 | P21730 |
| 86 | VLDLR | P98155 | VLDLR | P98155 |
| 87 | PPARD | Q03181 | PPARD | Q03181 |
| 88 | INSR | P06213 | INSR | P06213 |
| 89 | TNFRSF10A | O00220 | TNFRSF10A | O00220 |
| 90 | F7 | P08709 | F7 | P08709 |
| 91 | SOD1 | P00441 | SOD1 | P00441 |
| 92 | CCR5 | P51681 | CCR5 | P51681 |
| 93 | RXRA | P19793 | RXRA | P19793 |
| 94 | ESR2 | Q92731 | ESR2 | Q92731 |
| 95 | G6PD | P11413 | G6PD | P11413 |
| 96 | FLT1 | P17948 | FLT1 | P17948 |
| 97 | RBP4 | P02753 | RBP4 | P02753 |
| 98 | FABP1 | P07148 | FABP1 | P07148 |
| 99 | MMP3 | P08254 | MMP3 | P08254 |
| 100 | ATP5D | P30049 | ATP5D | P30049 |
| 101 | ERBB2 | P04626 | ERBB2 | P04626 |
| 102 | HSP90AA1 | P07900 | HSP90AA1 | P07900 |
| 103 | PRKCB | P05771 | PRKCB | P05771 |
| 104 | FADS1 | O60427 | FADS1 | O60427 |
| 105 | CFTR | P13569 | CFTR | P13569 |
| 106 | NFKB1 | P19838 | NFKB1 | P19838 |
| 107 | PIK3CB | P42338 | PIK3CB | P42338 |
| 108 | AURKA | Q9NWT8 | AURKA | Q9NWT8 |
| 109 | NLRP3 | Q96P20 | NLRP3 | Q96P20 |
| 110 | GCK | P35557 | GCK | P35557 |
| 111 | CCND1 | P24385 | CCND1 | P24385 |
| 112 | CYP2C9 | P11712 | CYP2C9 | P11712 |
| 113 | ACACA | Q13085 | ACACA | Q13085 |
| 114 | FOS | P01100 | FOS | P01100 |
| 115 | XIAP | P98170 | XIAP | P98170 |
| 116 | PSMD8 | P48556 | PSMD8 | P48556 |
| 117 | PRKCD | Q05655 | PRKCD | Q05655 |
| 118 | MT-CO2 | P00403 | MT-CO2 | P00403 |
| 119 | MT-CO1 | P00395 | MT-CO1 | P00395 |
| 120 | MT-CO3 | P00414 | MT-CO3 | P00414 |
| 121 | IFNG | P01579 | IFNG | P01579 |
| 122 | SLC2A4 | P14672 | SLC2A4 | P14672 |
| 123 | NOS2 | P35228 | NOS2 | P35228 |
| 124 | ITGAL | P20701 | ITGAL | P20701 |
| 125 | APP | P05067 | APP | P05067 |
| 126 | F10 | P00742 | F10 | P00742 |
| 127 | CFD | P00746 | CFD | P00746 |
| 128 | HSP90B1 | P14625 | HSP90B1 | P14625 |
| 129 | NR1I2 | O75469 | NR1I2 | O75469 |
| 130 | TP53 | P04637 | TP53 | P04637 |
| 131 | RARA | P10276 | RARA | P10276 |
| 132 | NR3C2 | P08235 | NR3C2 | P08235 |
| 133 | ACACB | O00763 | ACACB | O00763 |
| 134 | PSEN1 | P49768 | PSEN1 | P49768 |
| 135 | VDR | P11473 | VDR | P11473 |
| 136 | AHR | P35869 | AHR | P35869 |
| 137 | PSMA4 | P25789 | PSMA4 | P25789 |
| 138 | IL2 | P60568 | IL2 | P60568 |
| 139 | CCR2 | P41597 | CCR2 | P41597 |
| 140 | AKT1 | P31749 | AKT1 | P31749 |
| 141 | MMP2 | P08253 | MMP2 | P08253 |
| 142 | TGFB1 | P01137 | TGFB1 | P01137 |
| 143 | MMP14 | P50281 | MMP14 | P50281 |
| 144 | GSK3B | P49841 | GSK3B | P49841 |
| 145 | GYS1 | P13807 | GYS1 | P13807 |
| 146 | MMP7 | P09237 | MMP7 | P09237 |
| 147 | MMP1 | P03956 | MMP1 | P03956 |
| 148 | SLC27A1 | Q6PCB7 | SLC27A1 | Q6PCB7 |
| 149 | FKBP1A | P62942 | FKBP1A | P62942 |
| 150 | CREBBP | Q92793 | CREBBP | Q92793 |
| 151 | PLAU | P00749 | PLAU | P00749 |
| 152 | CASP3 | P42574 | CASP3 | P42574 |
| 153 | CREB1 | P16220 | CREB1 | P16220 |
| 154 | PSEN2 | P49810 | PSEN2 | P49810 |
| 155 | UGT1A1 | P22309 | UGT1A1 | P22309 |
| 156 | PTGS2 | P35354 | PTGS2 | P35354 |
| 157 | GSR | P00390 | GSR | P00390 |
| 158 | WNT3A | P56704 | WNT3A | P56704 |
| 159 | FGF2 | P09038 | FGF2 | P09038 |
| 160 | PTGS1 | P23219 | PTGS1 | P23219 |
| 161 | RXRB | P28702 | RXRB | P28702 |
| 162 | ALOX12 | P18054 | ALOX12 | P18054 |
| 163 | COX5A | P20674 | COX5A | P20674 |
| 164 | PSMA3 | P25788 | PSMA3 | P25788 |
| 165 | ACSL3 | O95573 | ACSL3 | O95573 |
| 166 | CYP2C19 | P33261 | CYP2C19 | P33261 |
| 167 | MAPK10 | P53779 | MAPK10 | P53779 |
| 168 | RELA | Q04206 | RELA | Q04206 |
| 169 | ALDH2 | P05091 | ALDH2 | P05091 |
| 170 | HIF1A | Q16665 | HIF1A | Q16665 |
| 171 | MAPK14 | Q16539 | MAPK14 | Q16539 |
| 172 | CASP8 | Q14790 | CASP8 | Q14790 |
| 173 | BCL2 | P10415 | BCL2 | P10415 |
| 174 | THRB | P10828 | THRB | P10828 |
| 175 | FURIN | P09958 | FURIN | P09958 |
| 176 | MAP2K1 | Q02750 | MAP2K1 | Q02750 |
| 177 | PPP3CA | Q08209 | PPP3CA | Q08209 |
| 178 | JAK3 | P52333 | JAK3 | P52333 |
| 179 | PIK3CA | P42336 | PIK3CA | P42336 |
| 180 | DNMT1 | P26358 | DNMT1 | P26358 |
| 181 | ITGB2 | P05107 | ITGB2 | P05107 |
| 182 | MAPK8 | P45983 | MAPK8 | P45983 |
| 183 | MAPT | P10636 | MAPT | P10636 |
| 184 | NFKBIA | P25963 | NFKBIA | P25963 |
| 185 | LIMK1 | P53667 | LIMK1 | P53667 |
| 186 | PTK2B | Q14289 | PTK2B | Q14289 |
| 187 | SIRT1 | Q96EB6 | SIRT1 | Q96EB6 |
| 188 | NOS1 | P29475 | NOS1 | P29475 |
| 189 | PLG | P00747 | PLG | P00747 |
| 190 | BAX | Q07812 | BAX | Q07812 |
| 191 | MMP8 | P22894 | MMP8 | P22894 |
| 192 | TNFRSF1A | P19438 | TNFRSF1A | P19438 |
| 193 | THRA | P10827 | THRA | P10827 |
| 194 | NFATC1 | O95644 | NFATC1 | O95644 |
| 195 | PIK3CG | P48736 | PIK3CG | P48736 |
| 196 | PGD | P52209 | PGD | P52209 |
| 197 | CASP9 | P55211 | CASP9 | P55211 |
| 198 | CDK1 | P06493 | CDK1 | P06493 |
| 199 | HDAC9 | Q9UKV0 | HDAC9 | Q9UKV0 |
| 200 | PPP3R1 | P63098 | PPP3R1 | P63098 |
| 201 | IL4 | P05112 | IL4 | P05112 |
| 202 | IL6ST | P40189 | IL6ST | P40189 |
| 203 | CALM1 | P0DP23 | CALM1 | P0DP23 |
| 204 | PSMB5 | P28074 | PSMB5 | P28074 |
| 205 | PSMC4 | P43686 | PSMC4 | P43686 |
| 206 | CASP1 | P29466 | CASP1 | P29466 |
| 207 | PLA2G1B | P04054 | PLA2G1B | P04054 |
| 208 | MYLK | Q15746 | MYLK | Q15746 |
| 209 | EPHB2 | P29323 | EPHB2 | P29323 |
| 210 | MAPK1 | P28482 | MAPK1 | P28482 |
| 211 | PON1 | P27169 | PON1 | P27169 |
| 212 | ADRB3 | P13945 | ADRB3 | P13945 |
| 213 | FABP2 | P12104 | FABP2 | P12104 |
| 214 | GBA | P04062 | GBA | P04062 |
| 215 | DGAT1 | O75907 | DGAT1 | O75907 |
| 216 | SOAT1 | P35610 | SOAT1 | P35610 |
| 217 | TRPC6 | Q9Y210 | TRPC6 | Q9Y210 |
| 218 | SHBG | P04278 | SHBG | P04278 |
| 219 | FBP1 | P09467 | FBP1 | P09467 |
| 220 | ACHE | P22303 | ACHE | P22303 |
| 221 | SLC10A2 | Q12908 | SLC10A2 | Q12908 |
| 222 | NAMPT | P43490 | NAMPT | P43490 |
| 223 | HAS2 | Q92819 | HAS2 | Q92819 |
| 224 | NPPB | P16860 | NPPB | P16860 |
| 225 | BRD4 | O60885 | BRD4 | O60885 |
| 226 | SCD | O00767 | SCD | O00767 |
| 227 | NPC1L1 | Q9UHC9 | NPC1L1 | Q9UHC9 |
| 228 | SOAT2 | O75908 | SOAT2 | O75908 |
| 229 | SLC8A1 | P32418 | SLC8A1 | P32418 |
| 230 | UGCG | Q16739 | UGCG | Q16739 |
| 231 | SLPI | P03973 | SLPI | P03973 |
| 232 | ADRB1 | P08588 | ADRB1 | P08588 |
| 233 | AKR1B1 | P15121 | AKR1B1 | P15121 |
| 234 | ADA | P00813 | ADA | P00813 |
| 235 | MC4R | P32245 | MC4R | P32245 |
| 236 | MT-ND6 | P03923 | MT-ND6 | P03923 |
| 237 | ENPP1 | P22413 | ENPP1 | P22413 |
| 238 | BCHE | P06276 | BCHE | P06276 |
| 239 | ABCC2 | Q92887 | ABCC2 | Q92887 |
| 240 | HSD11B1 | P28845 | HSD11B1 | P28845 |
| 241 | OPRK1 | P41145 | OPRK1 | P41145 |
| 242 | NR1I3 | Q14994 | NR1I3 | Q14994 |
| 243 | DPP4 | P27487 | DPP4 | P27487 |
| 244 | HNF4A | P41235 | HNF4A | P41235 |
| 245 | SLC6A4 | P31645 | SLC6A4 | P31645 |
| 246 | CYP2C8 | P10632 | CYP2C8 | P10632 |
| 247 | CYP11B2 | P19099 | CYP11B2 | P19099 |
| 248 | SERPINA6 | P08185 | SERPINA6 | P08185 |
| 249 | SLC10A1 | Q14973 | SLC10A1 | Q14973 |
| 250 | PLA2G10 | O15496 | PLA2G10 | O15496 |
| 251 | ACP1 | P24666 | ACP1 | P24666 |
| 252 | SLC6A3 | Q01959 | SLC6A3 | Q01959 |
| 253 | SLC6A2 | P23975 | SLC6A2 | P23975 |
| 254 | GCGR | P47871 | GCGR | P47871 |
| 255 | HPSE | Q9Y251 | HPSE | Q9Y251 |
| 256 | ACSL4 | O60488 | ACSL4 | O60488 |
| 257 | PDE5A | O76074 | PDE5A | O76074 |
| 258 | CALCA | P06881 | CALCA | P06881 |
| 259 | MME | P08473 | MME | P08473 |
| 260 | CASR | P41180 | CASR | P41180 |
| 261 | HTR2A | P28223 | HTR2A | P28223 |
| 262 | HTR2C | P28335 | HTR2C | P28335 |
| 263 | TRHR | P34981 | TRHR | P34981 |
| 264 | ABCG2 | Q9UNQ0 | ABCG2 | Q9UNQ0 |
| 265 | SLC5A1 | P13866 | SLC5A1 | P13866 |
| 266 | EDNRA | P25101 | EDNRA | P25101 |
| 267 | CHRM3 | P20309 | CHRM3 | P20309 |
| 268 | PDE4D | Q08499 | PDE4D | Q08499 |
| 269 | ABCC1 | P33527 | ABCC1 | P33527 |
| 270 | TACR1 | P25103 | TACR1 | P25103 |
| 271 | PLA2G2A | P14555 | PLA2G2A | P14555 |
| 272 | ERN1 | O75460 | ERN1 | O75460 |
| 273 | PRCP | P42785 | PRCP | P42785 |
| 274 | LGALS3 | P17931 | LGALS3 | P17931 |
| 275 | P2RY1 | P47900 | P2RY1 | P47900 |
| 276 | FADS2 | O95864 | FADS2 | O95864 |
| 277 | AKR1B10 | O60218 | AKR1B10 | O60218 |
| 278 | SLC5A4 | Q9NY91 | SLC5A4 | Q9NY91 |
| 279 | ATP5F1B | P06576 | ATP5F1B | P06576 |
| 280 | PLB1 | Q6P1J6 | PLB1 | Q6P1J6 |
| 281 | PAEP | P09466 | PAEP | P09466 |
| 282 | ESRRA | P11474 | ESRRA | P11474 |
| 283 | TOP2A | P11388 | APOE | P02649 |
| 284 | PTPN1 | P18031 | LIPC | P11150 |
| 285 | PTGES | O14684 | USF1 | P22415 |
| 286 | CES2 | O00748 | APOC3 | P02656 |
| 287 | CD81 | P60033 | CETP | P11597 |
| 288 | FNTA | P49354 | APOA5 | Q6Q788 |
| 289 | PTPN6 | P29350 | APOA1 | P02647 |
| 290 | PTGDR2 | Q9Y5Y4 | APOA2 | P02652 |
| 291 | SRD5A2 | P31213 | SLC37A4 | O43826 |
| 292 | CYSLTR1 | Q9Y271 | G6PC | P35575 |
| 293 | POLB | P06746 | NPHS1 | O60500 |
| 294 | PTGER2 | P43116 | ALB | P02768 |
| 295 | CDC25B | P30305 | NPHS2 | Q9NP85 |
| 296 | CYP51A1 | Q16850 | AGL | P35573 |
| 297 | RORC | P51449 | COG2 | Q14746 |
| 298 | PTPRF | P10586 | ACTN4 | O43707 |
| 299 | PTPN2 | P17706 | LCAT | P04180 |
| 300 | HSD11B2 | P80365 | C3 | P01024 |
| 301 | PREP | P48147 | PLCE1 | Q9P212 |
| 302 | SIGMAR1 | Q99720 | WT1 | P19544 |
| 303 | CYP17A1 | P05093 | APOC2 | P02655 |
| 304 | RASGRP3 | Q8IV61 | CRP | P02741 |
| 305 | HAO1 | Q9UJM8 | LPA | P08519 |
| 306 | FABP5 | Q01469 | APOA4 | P06727 |
| 307 | PTGER4 | P35408 | PRMT7 | Q9NVM4 |
| 308 | BACE1 | P56817 | MYO1E | Q12965 |
| 309 | OPRD1 | P41143 | DGKE | P52429 |
| 310 | PTGFR | P43088 | PHKA2 | P46019 |
| 311 | SAE1 | Q9UBE0 | TXNIP | Q9H3M7 |
| 312 | FABP3 | P05413 | SGPL1 | O95470 |
| 313 | PTPN11 | Q06124 | GPT | P24298 |
| 314 | TERT | O14746 | ADD1 | P35611 |
| 315 | PRKCH | P24723 | PIK3C2A | O00443 |
| 316 | ADORA3 | P0DMS8 | LEP | P41159 |
| 317 | MAPK3 | P27361 | ARHGDIA | P52565 |
| 318 | TOP1 | P11387 | PTPRO | Q16827 |
| 319 | RORA | P35398 | LAMB2 | P55268 |
| 320 | PGR | P06401 | MAGI2 | Q86UL8 |
| 321 | CDC25A | P30304 | NUP107 | P57740 |
| 322 | FFAR1 | O14842 | EMP2 | P54851 |
| 323 | FAAH | O00519 | NUP133 | Q8WUM0 |
| 324 | PTGIR | P43119 | NUP93 | Q8N1F7 |
| 325 | CHRM2 | P08172 | NUP85 | Q9BW27 |
| 326 | PTGER1 | P34995 | NUP160 | Q12769 |
| 327 | CTSD | P07339 | KANK2 | Q63ZY3 |
| 328 | IDO1 | P14902 | NUP205 | Q92621 |
| 329 | HSD17B3 | P37058 | TBC1D8B | Q0IIM8 |
| 330 | PTGER3 | P43115 | ABCG5 | Q9H222 |
| 331 | SLC22A6 | Q4U2R8 | ABCA1 | O95477 |
| 332 | CDC25C | P30307 | OLR1 | P78380 |
| 333 | LTB4R | Q15722 | ABCG8 | Q9H221 |
| 334 | TRPV1 | Q8NER1 | LMNB2 | Q03252 |
| 335 | GPBAR1 | Q8TDU6 | GHR | P10912 |
| 336 | ADH1C | P00326 | CRABP2 | P29373 |
| 337 | AKR1C1 | Q04828 | LDLRAP1 | Q5SW96 |
| 338 | AKR1C2 | P52895 | SLC17A5 | Q9NRA2 |
| 339 | ANXA1 | P04083 | GYS2 | P54840 |
| 340 | CLEC4E | Q9ULY5 | ZMPSTE24 | O75844 |
| 341 | COX4I1 | P13073 | LPXN | O60711 |
| 342 | COX5B | P10606 | LMNA | P02545 |
| 343 | COX6A2 | Q02221 | MTHFR | P42898 |
| 344 | COX6B1 | P14854 | PNLIP | P16233 |
| 345 | COX6C | P09669 | LIPA | P38571 |
| 346 | COX7A1 | P24310 | ALG1 | Q9BT22 |
| 347 | COX7B | P24311 | CELA2A | P08217 |
| 348 | COX7C | P15954 | TTC21B | Q7Z4L5 |
| 349 | COX8A | P10176 | APOC1 | P02654 |
| 350 | CYP27B1 | O15528 | CFH | P08603 |
| 351 | ESRRG | P62508 | SCARB1 | Q8WTV0 |
| 352 | FABP6 | P51161 | RAB27A | P51159 |
| 353 | GABRA1 | P14867 | MYO5A | Q9Y4I1 |
| 354 | GABRA2 | P47869 | CIDEC | Q96AQ7 |
| 355 | GABRA3 | P34903 | INF2 | Q27J81 |
| 356 | GABRA4 | P48169 | LRP6 | O75581 |
| 357 | GABRA5 | P31644 | LIPI | Q6XZB0 |
| 358 | GABRA6 | Q16445 | PLTP | P55058 |
| 359 | GABRB1 | P18505 | SELP | P16109 |
| 360 | GABRB2 | P47870 | GLA | P06280 |
| 361 | GABRB3 | P28472 | MGP | P08493 |
| 362 | GABRD | O14764 | CFHR5 | Q9BXR6 |
| 363 | GABRE | P78334 | PPP1R17 | O96001 |
| 364 | GABRG1 | Q8N1C3 | ANGPTL3 | Q9Y5C1 |
| 365 | GABRG2 | P18507 | TF | P02787 |
| 366 | GABRG3 | Q99928 | GCG | P01275 |
| 367 | GABRP | O00591 | CD2AP | Q9Y5K6 |
| 368 | GABRQ | Q9UN88 | THBD | P07204 |
| 369 | GRIN1 | Q05586 | SLC25A13 | Q9UJS0 |
| 370 | GRIN2A | Q12879 | SMPD1 | P17405 |
| 371 | GRIN2B | Q13224 | PC | P11498 |
| 372 | GRIN2C | Q14957 | TFG | Q92734 |
| 373 | GRIN2D | O15399 | VWF | P04275 |
| 374 | GRIN3A | Q8TCU5 | H2AC18 | Q6FI13 |
| 375 | GRIN3B | O60391 | ADCY3 | O60266 |
| 376 | GSTP1 | P09211 | HMBS | P08397 |
| 377 | HOXA10 | P31260 | PHKG2 | P15735 |
| 378 | HSD17B1 | P14061 | CFHR1 | Q03591 |
| 379 | HSD3B1 | P14060 | CFHR3 | Q02985 |
| 380 | IGHG2 | P01859 | DCAF17 | Q5H9S7 |
| 381 | LSS | P48449 | FGA | P02671 |
| 382 | NFKB2 | Q00653 | AGT | P01019 |
| 383 | PRLR | P16471 | SERPINC1 | P01008 |
| 384 | SULT2A1 | Q06520 | IGF1 | P05019 |
| 385 | SULT2B1 | O00204 | ANXA5 | P08758 |
| 386 | EPAS1 | Q99814 | HK1 | P19367 |
| 387 | TACR2 | P21452 | SLC34A2 | O95436 |
| 388 | S1PR2 | O95136 | KL | Q9UEF7 |
| 389 | EDNRB | P24530 | SLC2A9 | Q9NRM0 |
| 390 | EPHA2 | P29317 | FGF23 | Q9GZV9 |
| 391 | CALCRL | Q16602 | TRIM21 | P19474 |
| 392 | ITGAV | P06756 | G6PC3 | Q9BUM1 |
| 393 | HSD17B2 | P37059 | NEK3 | P51956 |
| 394 | IDH1 | O75874 | SLC37A1 | P57057 |
| 395 | CNR2 | P34972 | DCAF8 | Q5TAQ9 |
| 396 | GRM1 | Q13255 | SLC37A3 | Q8NCC5 |
| 397 | KCNH2 | Q12809 | SPNS1 | Q9H2V7 |
| 398 | S1PR3 | Q99500 | WDTC1 | Q8N5D0 |
| 399 | S1PR1 | P21453 | PAGR1 | Q9BTK6 |
| 400 | HCRTR2 | O43614 | LIME1 | Q9H400 |
| 401 | HCRTR1 | O43613 | KIRREL1 | Q96J84 |
| 402 | ADORA1 | P30542 | CRB2 | Q5IJ48 |
| 403 | ADORA2A | P29274 | PNPLA2 | Q96AD5 |
| 404 | PLK1 | P53350 | LRP1 | Q07954 |
| 405 | PDE10A | Q9Y233 | CHIT1 | Q13231 |
| 406 | KIF11 | P52732 | C5AR2 | Q9P296 |
| 407 | MAP3K20 | Q9NYL2 | OXA1L | Q15070 |
| 408 | TGFBR2 | P37173 | BGLAP | P02818 |
| 409 | TGFBR1 | P36897 | SLCO1B1 | Q9Y6L6 |
| 410 | MET | P08581 | LMAN1 | P49257 |
| 411 | AVPR1A | P37288 | AVP | P01185 |
| 412 | CRHR1 | P34998 | MCFD2 | Q8NI22 |
| 413 | AKR1C3 | P42330 | BAAT | Q14032 |
| 414 | GRIA2 | P42262 | SH2B1 | Q9NRF2 |
| 415 | ACKR3 | P25106 | ASXL2 | Q76L83 |
| 416 | MAPK9 | P45984 | CFHR4 | Q92496 |
| 417 | PDE2A | O00408 | AVIL | O75366 |
| 418 | CTSL | P07711 | AXDND1 | Q5T1B0 |
| 419 | PGGT1B | P53609 | HP | P00738 |
| 420 | ITK | Q08881 | AGER | Q15109 |
| 421 | P2RX3 | P56373 | POMC | P01189 |
| 422 | ALK | Q9UM73 | B2M | P61769 |
| 423 | CDC7 | O00311 | EDN1 | P05305 |
| 424 | KDR | P35968 | ANGPTL4 | Q9BY76 |
| 425 | CSF1R | P07333 | NPY | P01303 |
| 426 | CHRM1 | P11229 | GH1 | P01241 |
| 427 | MTNR1A | P48039 | PON2 | Q15165 |
| 428 | MTNR1B | P49286 | EPO | P01588 |
| 429 | SLC16A1 | P53985 | ITGA3 | P26006 |
| 430 | NTRK1 | P04629 | SMARCAL1 | Q9NZC9 |
| 431 | JAK2 | O60674 | COQ6 | Q9Y2Z9 |
| 432 | HPGDS | O60760 | PDSS2 | Q86YH6 |
| 433 | PRKCQ | Q04759 | WDR73 | Q6P4I2 |
| 434 | SMO | Q99835 | SRY | Q05066 |
| 435 | EZH2 | Q15910 | CD55 | P08174 |
| 436 | MERTK | Q12866 | GAA | P10253 |
| 437 | PRKCA | P17252 | UCP1 | P25874 |
| 438 | HSD3B2 | P26439 | ABCG1 | P45844 |
| 439 | MDM4 | O15151 | CCL5 | P13501 |
| 440 | CACNA2D1 | P54289 | PRL | P01236 |
| 441 | MMP13 | P45452 | PAPPA | Q13219 |
| 442 | MMP10 | P09238 | CD59 | P13987 |
| 443 | MMP12 | P39900 | CX3CR1 | P49238 |
| 444 | GLRA1 | P23415 | PTH | P01270 |
| 445 | DHCR7 | Q9UBM7 | NF1 | P21359 |
| 446 | PRKCG | P05129 | AFP | P02771 |
| 447 | PRKCE | Q02156 | SI | P14410 |
| 448 | SHH | Q15465 | APTX | Q7Z2E3 |
| 449 | CDK6 | Q00534 | PYGB | P11216 |
| 450 | TBXA2R | P21731 | TJP1 | Q07157 |
| 451 | DRD2 | P14416 | SHMT2 | P34897 |
| 452 | CCR1 | P32246 | GC | P02774 |
| 453 | ITGA2B | P08514 | GBE1 | Q04446 |
| 454 | ITGB5 | P18084 | MGAT4C | Q9UBM8 |
| 455 | ITGB1 | P05556 | SYNPO | Q8N3V7 |
| 456 | GPR17 | Q13304 | ARHGAP24 | Q8N264 |
| 457 | FYN | P06241 | STBD1 | O95210 |
| 458 | ADRA2A | P08913 | ALG1L | Q6GMV1 |
| 459 | ADRA2C | P18825 | FNDC11 | Q9BVV2 |
| 460 | DRD3 | P35462 | CPT1B | Q92523 |
| 461 | CCKBR | P32239 | FGB | P02675 |
| 462 | ECE1 | P42892 | TRIB1 | Q96RU8 |
| 463 | PTGDR | Q13258 | PLIN1 | O60240 |
| 464 | PGC | P20142 | BSCL2 | Q96G97 |
| 465 | NTSR1 | P30989 | HMGA1 | P17096 |
| 466 | PIM1 | P11309 | CDKN2A | Q8N726 |
| 467 | OGFRL1 | Q5TC84 | TPO | P07202 |
| 468 | IKBKB | O14920 | SERPINA7 | P05543 |
| 469 | TBXAS1 | P24557 | MYLIP | Q8WY64 |
| 470 | FKBP5 | Q13451 | ACADM | P11310 |
| 471 | PLA2G4A | P47712 | EGF | P01133 |
| 472 | FKBP4 | Q02790 | CBS | P35520 |
| 473 | IMPDH2 | P12268 | F5 | P12259 |
| 474 | HDAC2 | Q92769 | MSR1 | P21757 |
| 475 | IMPDH1 | P20839 | IGFBP1 | P08833 |
| 476 | CYP26A1 | O43174 | GPD1 | P21695 |
| 477 | TYMS | P04818 | RETN | Q9HD89 |
| 478 | F11 | P03951 | TXN | P10599 |
| 479 | ENPP2 | Q13822 | CYP21A2 | P08686 |
| 480 | ALOX15 | P16050 | SORL1 | Q92673 |
| 481 | FFAR2 | O15552 | SERPINA3 | P01011 |
| 482 | TRPM8 | Q7Z2W7 | GHRH | P01286 |
| 483 | AVPR2 | P30518 | LIPG | Q9Y5X9 |
| 484 | MC1R | Q01726 | PSMB8 | P28062 |
| 485 | MC5R | P33032 | CAV1 | Q03135 |
| 486 | METAP1 | P53582 | STXBP2 | Q15833 |
| 487 | TRPA1 | O75762 | EMD | P50402 |
| 488 | PDE4A | P27815 | AGPAT2 | O15120 |
| 489 | PDE4B | Q07343 | UNC13D | Q70J99 |
| 490 | ADRA1B | P35368 | STX11 | O75558 |
| 491 | SCN5A | Q14524 | CAVIN1 | Q6NZI2 |
| 492 | PDE3A | Q14432 | C1R | P00736 |
| 493 | AMPD2 | Q01433 | LEPR | P48357 |
| 494 | GRIK1 | P39086 | USP9X | Q93008 |
| 495 | GRIK2 | Q13002 | SCNN1A | P37088 |
| 496 | IGKC | P01834 | CFB | P00751 |
| 497 | SQLE | Q14534 | CFI | P05156 |
| 498 | UGT2B7 | P16662 | KDM6A | O15550 |
| 499 | ADRA1A | P35348 | FMR1 | Q06787 |
| 500 | OPRM1 | P35372 | FDXR | P22570 |
| 501 | CHRNA7 | P36544 | MLPH | Q9BV36 |
| 502 | HSP90AB1 | P08238 | TG | P01266 |
| 503 | CDK2 | P24941 | PHKG1 | Q16816 |
| 504 | CHEK1 | O14757 | AMH | P03971 |
| 505 | PRKACA | P17612 | AGRP | O00253 |
| 506 | CAMKK2 | Q96RR4 | PGM2 | Q96G03 |
| 507 | CDKN1A | P38936 | DAB2 | P98082 |
| 508 | EIF6 | P56537 | CRYGC | P07315 |
| 509 | JUN | P05412 | COCH | O43405 |
| 510 | AHSA1 | O95433 | GAS1 | P54826 |
| 511 | BBC3 | Q9BXH1 | BMP15 | O95972 |
| 512 | TEP1 | Q99973 | C8A | P07357 |
| 513 | MCL1 | Q07820 | MPV17 | P39210 |
| 514 | FLT3 | P36888 | CD93 | Q9NPY3 |
| 515 | NTRK2 | Q16620 | MICU1 | Q9BPX6 |
| 516 | KDM4E | B2RXH2 | SYTL2 | Q9HCH5 |
| 517 | GRK6 | P43250 | TNFRSF10C | O14798 |
| 518 | SLC22A12 | Q96S37 | LMF1 | Q96S06 |
| 519 | CYP1A1 | P04798 | GYG2 | O15488 |
| 520 | CYP1B1 | Q16678 | PGM5 | Q15124 |
| 521 | CA4 | P22748 | LYST | Q99698 |
| 522 | CA2 | P00918 | PSRC1 | Q6PGN9 |
| 523 | CA1 | P00915 | STAP1 | Q9ULZ2 |
| 524 | CA7 | P43166 | RPS4X | P62701 |
| 525 | CA12 | O43570 | SHOX | O15266 |
| 526 | CDK5R1 | Q15078 | GAS2 | O43903 |
| 527 | CCNB3 | Q8WWL7 | SHOX2 | O60902 |
| 528 | CA9 | Q16790 | ADPRH | P54922 |
| 529 | CBR1 | P16152 | CFHR2 | P36980 |
| 530 | LCK | P06239 | APEX2 | Q9UBZ4 |
| 531 | CA6 | P23280 | CMIP | Q8IY22 |
| 532 | PTPRS | Q13332 | COQ10A | Q96MF6 |
| 533 | DAPK1 | P53355 | SSNA1 | O43805 |
| 534 | MPG | P29372 | ZBTB8OS | Q8IWT0 |
| 535 | GPR35 | Q9HC97 | PPP1R3D | O95685 |
| 536 | PFKFB3 | Q16875 | BLOC1S1 | P78537 |
| 537 | CD38 | P28907 | TSPY1 | Q01534 |
| 538 | ARG1 | P05089 | OARD1 | Q9Y530 |
| 539 | CA3 | P07451 | SPAG17 | Q6Q759 |
| 540 | CA14 | Q9ULX7 | MACROD1 | Q9BQ69 |
| 541 | CA13 | Q8N1Q1 | EFHC2 | Q5JST6 |
| 542 | CAMK2B | Q13554 | RPS4Y1 | P22090 |
| 543 | CA5A | P35218 | PPP1R3E | Q9H7J1 |
| 544 | APEX1 | P27695 | CHURC1 | Q8WUH1 |
| 545 | AKR1C4 | P17516 | PDCL2 | Q8N4E4 |
| 546 | AKR1A1 | P14550 | PARPBP | Q9NWS1 |
| 547 | CYP1A2 | P05177 | CGB3 | P0N86 |
| 548 | ODC1 | P11926 | ADPRS | Q9NX46 |
| 549 | SRC | P12931 | TMSB15A | P0CG34 |
| 550 | TNKS | O95271 | CD14 | P08571 |
| 551 | TNKS2 | Q9H2K2 | GPIHBP1 | Q8IV16 |
| 552 | NAE1 | Q13564 | IL10 | P22301 |
| 553 | NOX4 | Q9NPH5 | F8 | P00451 |
| 554 | ACTB | P60709 | CST3 | P01034 |
| 555 | CEBPB | P17676 | SPP1 | P10451 |
| 556 | COMT | P21964 | LRP2 | P98164 |
| 557 | CSNK2A1 | P68400 | NPC2 | P61916 |
| 558 | CSNK2B | P67870 | HPX | P02790 |
| 559 | EIF3F | O00303 | MBL2 | P11226 |
| 560 | ESRRB | O95718 | GNRH1 | P01148 |
| 561 | GPER1 | Q99527 | CYBA | P13498 |
| 562 | HCK | P08631 | ASS1 | P00966 |
| 563 | HIBCH | Q6NVY1 | IL18 | Q14116 |
| 564 | HSPA2 | P54652 | MB | P02144 |
| 565 | IGHG1 | P01857 | CHKA | P35790 |
| 566 | JAK1 | P23458 | PTEN | P60484 |
| 567 | NQO2 | P16083 | ADAMTS13 | Q76LX8 |
| 568 | RUVBL2 | Q9Y230 | DDIT3 | P0DPQ6 |
| 569 | SF3B3 | Q15393 | ELN | P15502 |
| 570 | STK17B | O94768 | TUG1 | A0A6I8PU40 |
| 571 | UBA1 | P22314 | CYP27A1 | Q02318 |
| 572 | UGT3A1 | Q6NUS8 | GNB3 | P16520 |
| 573 | AMPD3 | Q01432 | ST3GAL4 | Q11206 |
| 574 | LANCL2 | Q9NS86 | TLR2 | O60603 |
| 575 | KDM5B | Q9UGL1 | GGT1 | P19440 |
| 576 | KDM4D | Q6B0I6 | HGF | P14210 |
| 577 | KDM4C | Q9H3R0 | PRKAG2 | Q9UGJ0 |
| 578 | IGF1R | P08069 | CPT2 | P23786 |
| 579 | ACLY | P53396 | TNNI3 | P19429 |
| 580 | TEK | Q02763 | TPM1 | P09493 |
| 581 | KDM4A | O75164 | KCNN4 | O15554 |
| 582 | CTSA | P10619 | HADHB | P55084 |
| 583 | MKNK2 | Q9HBH9 | NPC1 | O15118 |
| 584 | ANPEP | P15144 | LMNB1 | P20700 |
| 585 | CTNNB1 | P35222 | PCYT1A | P49585 |
| 586 | BMP1 | P13497 | HSPD1 | P10809 |
| 587 | CPA1 | P15085 | DES | P17661 |
| 588 | CXCR2 | P25025 | ARSA | P15289 |
| 589 | WEE1 | P30291 | HADHA | P40939 |
| 590 | AURKA | O14965 | TGFBR3 | Q03167 |
| 591 | MMEL1 | Q495T6 | PSAP | P07602 |
| 592 | ABL1 | P00519 | TRPV6 | Q9H1D0 |
| 593 | MAP3K9 | P80192 | UNG | P13051 |
| 594 | BTK | Q06187 | SH2D1A | O60880 |
| 595 | EGLN1 | Q9GZT9 | SERPING1 | P05155 |
| 596 | FOLH1 | Q04609 | ACADS | P16219 |
| 597 | SRD5A1 | P18405 | ACADVL | P49748 |
| 598 | HSD17B7 | P56937 | CD46 | P15529 |
| 599 | DRD1 | P21728 | C1S | P09871 |
| 600 | F2R | P25116 | MEN1 | O00255 |
| 601 | BRD2 | P25440 | FUCA1 | P04066 |
| 602 | KANSL3 | Q9P2N6 | C5 | P01031 |
| 603 | MAOB | P27338 | MASP1 | P48740 |
| 604 | TAS2R31 | P59538 | ECHS1 | P30084 |
| 605 | GRM5 | P41594 | AKR1D1 | P51857 |
| 606 | SLC5A2 | P31639 | ASNS | P08243 |
| 607 | PLA2G5 | P39877 | ALAD | P13716 |
| 608 | KLK1 | P06870 | HMGA2 | P52926 |
| 609 | KLK2 | P20151 | CR1 | P17927 |
| 610 | CA5B | Q9Y2D0 | PRODH | O43272 |
| 611 | HSD17B14 | Q9BPX1 | LAMP2 | P13473 |
| 612 | CLK1 | P49759 | TRPV5 | Q9NQA5 |
| 613 | DYRK1B | Q9Y463 | UROD | P06132 |
| 614 | DYRK1A | Q13627 | ZYX | Q15942 |
| 615 | SIRT2 | Q8IXJ6 | FHL2 | Q14192 |
| 616 | BCL2L1 | Q07817 | AP3B1 | O00203 |
| 617 | ADCY5 | O95622 | ALAS2 | P22557 |
| 618 | PGF | P49763 | CRAT | P43155 |
| 619 | YWHAG | P61981 | TNFSF13 | O75888 |
| 620 | VCP | P55072 | PIGA | P37287 |
| 621 | SYK | P43405 | CD34 | P28906 |
| 622 | GOT1 | P17174 | NAGA | P17050 |
| 623 | ABAT | P80404 | ATP2B1 | P20020 |
| 624 | AURKB | Q96GD4 | ALAS1 | P13196 |
| 625 | TOP2B | Q02880 | CPOX | P36551 |
| 626 | KCNMA1 | Q12791 | RGS9 | O75916 |
| 627 | CD163 | Q86VB7 | COX10 | Q12887 |
| 628 | MLXIPL | Q9NP71 | CFP | P27918 |
| 629 | ACOX1 | Q15067 | FUS | P35637 |
| 630 | CHRM4 | P08173 | SPRED1 | Q7Z699 |
| 631 | HTR7 | P34969 | IQGAP1 | P46940 |
| 632 | HTR1A | P08908 | LAMP1 | P11279 |
| 633 | ADRA1D | P25100 | UROS | P10746 |
| 634 | DRD4 | P21917 | PON3 | Q15166 |
| 635 | DRD5 | P21918 | PPOX | P50336 |
| 636 | MAOA | P21397 | LPP | Q93052 |
| 637 | CHRNB4 | P30926 | C4A | P0C0L4 |
| 638 | CHRNA3 | P32297 | C3AR1 | Q16581 |
| 639 | RBBP9 | O75884 | MASP2 | O00187 |
| 640 | KCNN1 | Q92952 | M6PR | P20645 |
| 641 | KCNN3 | Q9UGI6 | MYO5B | Q9ULV0 |
| 642 | KCNN2 | Q9H2S1 | MTMR3 | Q13615 |
| 643 | RPS6KA3 | P51812 | NFIB | O00712 |
| 644 | RPS6KB1 | P23443 | EWSR1 | Q01844 |
| 645 | CCNA1 | P78396 | AP2M1 | Q96CW1 |
| 646 | SLC1A3 | P43003 | A4GALT | Q9NPC4 |
| 647 | CXCR3 | P49682 | MMACHC | Q9Y4U1 |
| 648 | HTR2B | P41595 | SRI | P30626 |
| 649 | XBP1 | P17861 | TSFM | P43897 |
| 650 | SLC18A2 | Q05940 | TMLHE | Q9NVH6 |
| 651 | GRIA1 | P42261 | UMOD | P07911 |
| 652 | CCNE2 | O96020 | RAPGEF1 | Q13905 |
| 653 | ATR | Q13535 | C4B | P0C0L5 |
| 654 | PARP2 | Q9UGN5 | CPT1C | Q8TCG5 |
| 655 | TTK | P33981 | FCN3 | O75636 |
| 656 | CHRNA4 | P43681 | RAB11FIP2 | Q7L804 |
| 657 | CCNC | P24863 | XRCC2 | O43543 |
| 658 | CDK8 | P49336 | PLAG1 | Q6DJT9 |
| 659 | MAPKAPK2 | P49137 | HSD3B7 | Q9H2F3 |
| 660 | CYP11B1 | P15538 | PNPLA3 | Q9NST1 |
| 661 | CDK9 | P50750 | SOX18 | P35713 |
| 662 | HPGD | P15428 | AMBP | P02760 |
| 663 | TUBB3 | Q13509 | ANGPTL1 | O95841 |
| 664 | PGK1 | P00558 | FCN2 | Q15485 |
| 665 | ALDH3A1 | P30838 | UNC13B | O14795 |
| 666 | QPCT | Q16769 | PITX3 | O75364 |
| 667 | CHRM5 | P08912 | SLC15A2 | Q16348 |
| 668 | HTR3A | P46098 | GGA3 | Q9NZ52 |
| 669 | AMY1A | P0DUB6 | CCBE1 | Q6UXH8 |
| 670 | PARP1 | P09874 | FLVCR1 | Q9Y5Y0 |
| 671 | GLO1 | Q04760 | MLANA | Q16655 |
| 672 | TYR | P14679 | TRIP6 | Q15654 |
| 673 | AXL | P30530 | C4BPB | P20851 |
| 674 | ST6GAL1 | P15907 | HPS4 | Q9NQG7 |
| 675 | PRKDC | P78527 | HPS5 | Q9UPZ3 |
| 676 | FOSL1 | P15407 | HPS6 | Q86YV9 |
| 677 | FOSL2 | P15408 | AGFG1 | P52594 |
| 678 | CCNB1 | P14635 | LIN9 | Q5TKA1 |
| 679 | CYCS | P99999 | OLFM2 | O95897 |
| 680 | TDRD7 | Q8NHU6 | CARD8 | Q9Y2G2 |
| 681 | NOX5 | Q96PH1 | MYBPH | Q13203 |
| 682 | APOD | P05090 | MYO5C | Q9NQX4 |
| 683 | NMUR2 | Q9GZQ4 | MYRIP | Q8NFW9 |
| 684 | FXR1 | P51114 | NOC2L | Q9Y3T9 |
| 685 | CHRNA2 | Q15822 | AGBL5 | Q8NDL9 |
| 686 | MAP2 | P11137 | TSPAN31 | Q12999 |
| 687 | LTA4H | P09960 | PNPLA4 | P41247 |
| 688 | CTRB1 | P17538 | RBMS2 | Q15434 |
| 689 | ADH5 | P11766 | IGSF9 | Q9P2J2 |
| 690 | ARF1 | P84077 | SYTL3 | Q4VX76 |
| 691 | ARF6 | P62330 | VSIG4 | Q9Y279 |
| 692 | C8G | P07360 | PNPLA1 | Q8N8W4 |
| 693 | ECI2 | O75521 | FLG2 | Q5D862 |
| 694 | ELOVL4 | Q9GZR5 | BLOC1S3 | Q6QNY0 |
| 695 | GLTP | Q9NZD2 | AGBL2 | Q5U5Z8 |
| 696 | GM2A | P17900 | HORMAD2 | Q8N7B1 |
| 697 | GUCA1A | P43080 | CENPO | Q9BU64 |
| 698 | HNF4G | Q14541 | SMG5 | Q9UPR3 |
| 699 | LALBA | P00709 | MFSD10 | Q14728 |
| 700 | LTB4R2 | Q9NPC1 | MED18 | Q9BUE0 |
| 701 | LY96 | Q9Y6Y9 | AGBL3 | Q8NEM8 |
| 702 | PKIA | P61925 | AGBL1 | Q96MI9 |
| 703 | PLA2G2D | Q9UNK4 | PNPLA5 | Q7Z6Z6 |
| 704 | PLA2G2E | Q9NZK7 | RILPL2 | Q969X0 |
| 705 | PMP2 | P02689 | NOC3L | Q8WTT2 |
| 706 | PPT1 | P50897 | ALX3 | O95076 |
| 707 | PVR | P15151 | AGBL4 | Q5VU57 |
| 708 | RCVRN | P35243 | LMF2 | Q9BU23 |
| 709 | S100B | P04271 | RFESD | Q8TAC1 |
| 710 | SEC14L2 | O76054 | ZNF697 | Q5TEC3 |
| 711 | TRAPPC3 | O43617 | UTP4 | Q969X6 |
| 712 | ADK | P55263 | SELENOS | Q9BQE4 |
| 713 | HRAS | P01112 | NPB | Q8NG41 |
| 714 | TYMP | P19971 | C11orf95 | C9JLR9 |
| 715 | PNP | P00491 | AFG1L | Q8WV93 |
| 716 | LGALS9 | O00182 | LHFPL6 | Q9Y693 |
| 717 | OGA | O60502 | FAM104B | Q5XKR9 |
| 718 | SLC29A1 | Q99808 | RPEL1 | Q2QD12 |
| 719 | ADORA2B | P29275 | FAM223A | Q8IWN6 |
| 720 | CCNA2 | P20248 | IRS1 | P35568 |
| 721 | CSNK1G1 | Q9HCP0 | CD36 | P16671 |
| 722 | RPS6KA1 | Q15418 | CP | P00450 |
| 723 | ROCK1 | Q13464 | TIMP2 | P16035 |
| 724 | RET | P07949 | CHKB | Q9Y259 |
| 725 | EZR | P15311 | MTR | Q99707 |
| 726 | GRM4 | Q14833 | CYP2D6 | P10635 |
| 727 | DAO | P14920 | CXCL12 | P48061 |
| 728 | ALPG | P10696 | MTRR | Q9UBK8 |
| 729 | PLAA | Q9Y263 | CD79A | P11912 |
| 730 | HDAC4 | P56524 | GK | P32189 |
| 731 | CHEK2 | O96017 | ACAT2 | Q9BWD1 |
| 732 | GRK2 | P25098 | DEFA1 | P59665 |
| 733 | CCND3 | P30281 | CLU | P10909 |
| 734 | TUBB1 | Q9H4B7 | RGS2 | P41220 |
| 735 | PIK3CD | O00329 | AGTR2 | P50052 |
| 736 | PI4KB | Q9UBF8 | SAA1 | P0DJI8 |
| 737 | EPHB4 | P54760 | FABP12 | A6NFH5 |
| 738 | TBK1 | Q9UHD2 | ALPP | P05187 |
| 739 | RAF1 | P04049 | SULT1A3 | P0DMM9 |
| 740 | PIM2 | Q9P1W9 | NNMT | P40261 |
| 741 | PIM3 | Q86V86 | CDKN2B | P42772 |
| 742 | LNPEP | Q9UIQ6 | FASLG | P48023 |
| 743 | MKNK1 | Q9BUB5 | IL1A | P01583 |
| 744 | MAP3K8 | P41279 | ELOVL5 | Q9NYP7 |
| 745 | KDM1A | O60341 | ADM | P35318 |
| 746 | ALPL | P05186 | LPGAT1 | Q92604 |
| 747 | HDAC5 | Q9UQL6 | PIK3C2G | O75747 |
| 748 | HDAC7 | Q8WUI4 | ACP5 | P13686 |
| 749 | ADAM17 | P78536 | F11R | Q9Y624 |
| 750 | NEK6 | Q9HC98 | HAMP | P81172 |
| 751 | NUAK1 | O60285 | SPINK1 | P00995 |
| 752 | PTK2 | Q05397 | FGFR4 | P22455 |
| 753 | CCR4 | P51679 | HSPB1 | P04792 |
| 754 | GPR84 | Q9NQS5 | RHO | P08100 |
| 755 | MIF | P14174 | PPARGC1A | Q9UBK2 |
| 756 | TLR9 | Q9NR96 | CSF2 | P04141 |
| 757 | DHODH | Q02127 | ABCB7 | O75027 |
| 758 | STS | P08842 | SGCB | Q16585 |
| 759 | ERCC5 | P28715 | APOF | Q13790 |
| 760 | FEN1 | P39748 | FADS3 | Q9Y5Q0 |
| 761 | BAD | Q92934 | LMX1B | O60663 |
| 762 | PDGFRB | P09619 | ALMS1 | Q8TCU4 |
| 763 | GSK3A | P49840 | PRKG1 | Q13976 |
| 764 | CASP6 | P55212 | VEGFB | P49765 |
| 765 | CASP7 | P55210 | CLOCK | O15516 |
| 766 | ACVR1 | Q04771 | ARNTL | O00327 |
| 767 | ADAMTS5 | Q9UNA0 | CXCL2 | P19875 |
| 768 | CCNE1 | P24864 | MNDA | P41218 |
| 769 | PDE4C | Q08493 | PCDH15 | Q96QU1 |
| 770 | ADAMTS4 | O75173 | LSR | Q86X29 |
| 771 | PDK1 | Q15118 | CCL3 | P10147 |
| 772 | HDAC6 | Q9UBN7 | FGL1 | Q08830 |
| 773 | HDAC8 | Q9BY41 | CDH5 | P33151 |
| 774 | HDAC1 | Q13547 | LRAT | O95237 |
| 775 | HDAC3 | O15379 | SLC23A2 | Q9UGH3 |
| 776 | MARK1 | Q9P0L2 | SCAP | Q12770 |
| 777 | PDGFRA | P16234 | APOH | P02749 |
| 778 | NPY5R | Q15761 | SERPINA1 | P01009 |
| 779 | STAT6 | P42226 | WWOX | Q9NZC7 |
| 780 | YES1 | P07947 | TNFRSF11B | O00300 |
| 781 | BLK | P51451 | CD40LG | P29965 |
| 782 | CSK | P41240 | APOBEC1 | P41238 |
| 783 | ROCK2 | O75116 | JAG1 | P78504 |
| 784 | BMX | P51813 | PSMB9 | P28065 |
| 785 | CTSK | P43235 | POLD1 | P28340 |
| 786 | PDPK1 | O15530 | SLC7A7 | Q9UM01 |
| 787 | DPP7 | Q9UHL4 | FHL1 | Q13642 |
| 788 | DPP8 | Q6V1X1 | ABHD5 | Q8WTS1 |
| 789 | SGK1 | O00141 | PSMB4 | P28070 |
| 790 | DUT | P33316 | HAVCR2 | Q8TDQ0 |
| 791 | EPHA5 | P54756 | XRCC4 | Q13426 |
| 792 | EPHA4 | P54764 | RSPO1 | Q2MKA7 |
| 793 | EPHA8 | P29322 | POLR3A | O14802 |
| 794 | EPHA7 | Q15375 | SLC29A3 | Q9BZD2 |
| 795 | EPHB3 | P54753 | FLII | Q13045 |
| 796 | EPHA3 | P29320 | SYNE1 | Q8NF91 |
| 797 | EPHB1 | P54762 | TTPA | P49638 |
| 798 | EPHA1 | P21709 | DEAF1 | O75398 |
| 799 | CMA1 | P23946 | RAI1 | Q7Z5J4 |
| 800 | SPHK2 | Q9NRA0 | PIGH | Q14442 |
| 801 | SPHK1 | Q9NYA1 | SYNE2 | Q8WXH0 |
| 802 | CAPN1 | P07384 | TMEM43 | Q9BTV4 |
| 803 | OPRL1 | P41146 | NSMCE2 | Q96MF7 |
| 804 | DNM1 | Q05193 | IQSEC2 | Q5JU85 |
| 805 | MMP16 | P51512 | PLVAP | Q9BX97 |
| 806 | MMP15 | P51511 | CEP19 | Q96LK0 |
| 807 | SCN9A | Q15858 | SOD2 | P04179 |
| 808 | BACE2 | Q9Y5Z0 | KNG1 | P01042 |
| 809 | MGLL | Q99685 | CCK | P06307 |
| 810 | ABHD6 | Q9BV23 | ZAP70 | P43403 |
| 811 | KCNA3 | P22001 | IRAK1 | P51617 |
| 812 | NR1D1 | P20393 | NR5A1 | Q13285 |
| 813 | MELK | Q14680 | GSN | P06396 |
| 814 | MMP25 | Q9NPA2 | GATA3 | P23771 |
| 815 | ADAM10 | O14672 | MAP3K1 | Q13233 |
| 816 | PNMT | P11086 | LIG4 | P49917 |
| 817 | GSTM2 | P28161 | IL2RG | P31785 |
| 818 | FCER2 | P06734 | GATA4 | P43694 |
| 819 | SNCA | P37840 | CASP10 | Q92851 |
| 820 | DUSP3 | P51452 | IL7R | P16871 |
| 821 | ADAM9 | Q13443 | IFIH1 | Q9BYX4 |
| 822 | CXCR4 | P61073 | LYZ | P61626 |
| 823 | MPEG1 | Q2M385 | CHD7 | Q9P2D1 |
| 824 | CTSS | P25774 | STAT4 | Q14765 |
| 825 | GLI2 | P10070 | PAX2 | Q02962 |
| 826 | GLI1 | P08151 | SOX9 | P48436 |
| 827 | SLC9A1 | P19634 | PMM2 | O15305 |
| 828 | MAP4K4 | O95819 | C1QBP | Q07021 |
| 829 | CNOT7 | Q9UIV1 | FOXP3 | Q9BZS1 |
| 830 | KISS1R | Q969F8 | COL4A3 | Q01955 |
| 831 | XPO1 | O14980 | RAG1 | P15918 |
| 832 | MPI | P34949 | C1QA | P02745 |
| 833 | EP300 | Q09472 | COL4A4 | P53420 |
| 834 | PLEC | Q15149 | SCARB2 | Q14108 |
| 835 | PDE7A | Q13946 | MEFV | O15553 |
| 836 | TAOK1 | Q7L7X3 | DCLRE1C | Q96SD1 |
| 837 | TAOK3 | Q9H2K8 | COQ2 | Q96H96 |
| 838 | IKBKE | Q14164 | COL4A5 | P29400 |
| 839 | PTAFR | P25105 | RAG2 | P55895 |
| 840 | CTSB | P07858 | TRIM32 | Q13049 |
| 841 | NCOR2 | Q9Y618 | VAMP7 | P51809 |
| 842 | NCOR1 | O75376 | NPHP1 | O15259 |
| 843 | HDAC11 | Q96DB2 | SNAP29 | O95721 |
| 844 | HDAC10 | Q969S8 | CEP290 | O15078 |
| 845 | LIMK2 | P53671 | TP53RK | Q96S44 |
| 846 | CSNK1A1 | P48729 | TBX18 | O95935 |
| 847 | CDK13 | Q14004 | ZFPM2 | Q8WW38 |
| 848 | PTK6 | Q13882 | SDCCAG8 | Q86SQ7 |
| 849 | FGFR2 | P21802 | OSGEP | Q9NPF4 |
| 850 | DBF4 | Q9UBU7 | CHST14 | Q8NCH0 |
| 851 | RPS6KA2 | Q15349 | ARL6 | Q9H0F7 |
| 852 | ELANE | P08246 | BBS2 | Q9BXC9 |
| 853 | MBD2 | Q9UBB5 | BBS4 | Q96RK4 |
| 854 | CLK3 | P49761 | ANLN | Q9NQW6 |
| 855 | CSNK1D | P48730 | MKS1 | Q9NXB0 |
| 856 | TNNC1 | P63316 | SLC35A2 | P78381 |
| 857 | NR4A1 | P22736 | MKKS | Q9NPJ1 |
| 858 | HTT | P42858 | IFT172 | Q9UG01 |
| 859 | RIPK2 | O43353 | TTC8 | Q8TAM2 |
| 860 | EIF4A1 | P60842 | LZTFL1 | Q9NQ48 |
| 861 | SLC28A3 | Q9HAS3 | BBS9 | Q3SYG4 |
| 862 | CDC42BPA | Q5VT25 | BBS10 | Q8TAM1 |
| 863 | WDR5 | P61964 | TPRKB | Q9Y3C4 |
| 864 | DHFR | P00374 | WDR4 | P57081 |
| 865 | ACVRL1 | P37023 | BBS7 | Q8IWZ6 |
| 866 | BMP4 | P12644 | BBS1 | Q8NFJ9 |
| 867 | GSTA1 | P08263 | BBS5 | Q8N3I7 |
| 868 | KDM5A | P29375 | IFT27 | Q9BW83 |
| 869 | SCN2A | Q99250 | ZNF592 | Q92610 |
| 870 | TACR3 | P29371 | LAGE3 | Q14657 |
| 871 | TNK2 | Q07912 | WDPCP | O95876 |
| 872 | DMPK | Q09013 | C8orf37 | Q96NL8 |
| 873 | TSPO | P30536 | MARS1 | P56192 |
| 874 | PITRM1 | Q5JRX3 | BBS12 | Q6ZW61 |
| 875 | ROS1 | P08922 | MT-ND4 | P03905 |
| 876 | KCNA5 | P22460 | MT-ND5 | P03915 |
| 877 | FLT4 | P35916 | DMRT3 | Q9NQL9 |
| 878 | PLK4 | O00444 | MT-ND1 | P03886 |
| 879 | FGR | P09769 | BBIP1 | A8MTZ0 |
| 880 | LYN | P07948 | CD40 | P25942 |
| 881 | DPEP1 | P16444 | GHRL | Q9UBU3 |
| 882 | RPS6KA5 | O75582 | FOXC2 | Q99958 |
| 883 | GRM2 | Q14416 | CD63 | P08962 |
| 884 | PAK4 | O96013 | IFNA1 | L0N195 |
| 885 | CTSV | O60911 | P4HB | P07237 |
| 886 | P2RX7 | Q99572 | ACAT1 | P24752 |
| 887 | DYRK2 | Q92630 | IL2RB | P14784 |
| 888 | CDK5 | Q00535 | FGF21 | Q9NSA1 |
| 889 | CLK4 | Q9HAZ1 | HCAR2 | Q8TDS4 |
| 890 | CLK2 | P49760 | FAS | P25445 |
| 891 | DYRK3 | O43781 | NPPA | P01160 |
| 892 | PRKCZ | Q05513 | GNMT | Q14749 |
| 893 | ALOX15B | O15296 | DUSP2 | Q05923 |
| 894 | ADRA2B | P18089 | NEDD4 | P46934 |
| 895 | TAAR1 | Q96RJ0 | SERPINF2 | P08697 |
| 896 | NEK1 | Q96PY6 | GAL | P22466 |
| 897 | ME1 | P48163 | CLEC4A | Q9UMR7 |
| 898 | TXK | P42681 | ITGA4 | P13612 |
| 899 | EPHA6 | Q9UF33 | ITGAM | P11215 |
| 900 | TYRO3 | Q06418 | KLK3 | P07288 |
| 901 | NOX1 | Q9Y5S8 | DPAGT1 | Q9H3H5 |
| 902 | IRAK4 | Q9NWZ3 | ATF3 | P18847 |
| 903 | MAP2K2 | P36507 | PF4 | P02776 |
| 904 | LDHA | P00338 | OSBPL9 | Q96SU4 |
| 905 | PDE6C | P51160 | HCAR3 | P49019 |
| 906 | ADAM33 | Q9BZ11 | CYP3A5 | P20815 |
| 907 | OXTR | P30559 | CEL | P19835 |
| 908 | PDF | Q9HBH1 | GPX3 | P22352 |
| 909 | SMARCA2 | P51531 | UCP3 | P55916 |
| 910 | STAT3 | P40763 | APOL1 | O14791 |
| 911 | TGM2 | P21980 | HCRT | O43612 |
| 912 | ST3GAL3 | Q11203 | IFNB1 | P01574 |
| 913 | FUT7 | Q11130 | TNFSF14 | O43557 |
| 914 | FUT4 | P22083 | AS3MT | Q9HBK9 |
| 915 | STAT1 | P42224 | GJA1 | P17302 |
| 916 | NEK2 | P51955 | BRCA1 | P38398 |
| 917 | KCNK2 | O95069 | MYD88 | Q99836 |
| 918 | PSMD3 | O43242 | BRCA2 | P51587 |
| 919 | DIO1 | P49895 | LRP5 | O75197 |
| 920 | GSTM1 | P09488 | HSPA5 | P11021 |
| 921 | UTS2R | Q9UKP6 | PRDX2 | P32119 |
| 922 | MLYCD | O95822 | IGF2R | P11717 |
| 923 | L3MBTL3 | Q96JM7 | F9 | P00740 |
| 924 | PDE9A | O76083 | CPE | P16870 |
| 925 | L3MBTL1 | Q9Y468 | FTO | Q9C0B1 |
| 926 | BAZ2B | Q9UIF8 | NISCH | Q9Y2I1 |
| 927 | CECR2 | Q9BXF3 | ITLN1 | Q8WWA0 |
| 928 | BAZ2A | Q9UIF9 | RHOD | O00212 |
| 929 | PARP3 | Q9Y6F1 | MMRN1 | Q13201 |
| 930 | PARP4 | Q9UKK3 | SLC52A3 | Q9NQ40 |
| 931 | CACNA1G | O43497 | OSBPL10 | Q9BXB5 |
| 932 | SLC6A9 | P48067 | MVK | Q03426 |
| 933 | GPR55 | Q9Y2T6 | SLC12A3 | P55017 |
| 934 | CHRNB1 | P11230 | ATF6 | P18850 |
| 935 | TRPV3 | Q8NET8 | BDNF | P23560 |
| 936 | CELA1 | Q9UNI1 | WNK1 | Q9H4A3 |
| 937 | BIRC2 | Q13490 | FMO3 | P31513 |
| 938 | BDKRB1 | P46663 | MMAB | Q96EY8 |
| 939 | CTSE | P14091 | TFPI | P10646 |
| 940 | GHSR | Q92847 | IRS2 | Q9Y4H2 |
| 941 | HRH3 | Q9Y5N1 | F13A1 | P00488 |
| 942 | LAP3 | P28838 | WNK4 | Q96J92 |
| 943 | MCHR1 | Q99705 | GALNT2 | Q10471 |
| 944 | PDE11A | Q9HCR9 | HBA1 | Q9BX83 |
| 945 | PDE1C | Q14123 | SELL | P14151 |
| 946 | PDE8B | O95263 | CRABP1 | P29762 |
| 947 | HTR4 | Q13639 | KLF2 | Q9Y5W3 |
| 948 | TYK2 | P29597 | OSBP | P22059 |
| 949 | CDA | P32320 | OSBPL2 | Q9H1P3 |
| 950 | CYP26B1 | Q9NR63 | SULF2 | Q8IWU5 |
| 951 | RARG | P13631 | RBM4 | Q9BWF3 |
| 952 | RARB | P10826 | USF2 | Q15853 |
| 953 | NR0B2 | Q15466 | GABPA | Q06546 |
| 954 | PTGES2 | Q9H7Z7 | ADAMTS7 | Q9UKP4 |
| 955 | FFAR4 | Q5NUL3 | CERS4 | Q9HA82 |
| 956 | DAGLA | Q9Y4D2 | GABPB1 | Q06547 |
| 957 | OXER1 | Q8TDS5 | CCDC92 | Q53HC0 |
| 958 | KDM2A | Q9Y2K7 | ITGB3 | P05106 |
| 959 | KDM5C | P41229 | IL1RN | P18510 |
| 960 | CTSG | P08311 | TCF7L2 | Q9NQB0 |
| 961 | KEAP1 | Q14145 | CD44 | P16070 |
| 962 | PRKAG1 | P54619 | F2RL1 | P55085 |
| 963 | NQO1 | P15559 | CKM | P06732 |
| 964 | ATP1A1 | P05023 | CSF1 | P09603 |
| 965 | ATP1A2 | P50993 | PPBP | P02775 |
| 966 | ATP1A3 | P13637 | PPIG | Q13427 |
| 967 | GLRA3 | O75311 | LTA | P01374 |
| 968 | PPP1CC | P36873 | ASGR2 | P07307 |
| 969 | SLCO1B3 | Q9NPD5 | GLUD1 | P00367 |
| 970 | YWHAE | P62258 | MAPK7 | Q13164 |
| 971 | FGF1 | P05230 | EIF2AK3 | Q9NZJ5 |
| 972 | LGALS4 | P56470 | GNAI2 | P04899 |
| 973 | LGALS8 | O00214 | TAP1 | Q03518 |
| 974 | ADCYAP1 | P18509 | GPX1 | P07203 |
| 975 | PSMG1 | O95456 | PLD2 | O14939 |
| 976 | MAP2K4 | P45985 | MSX2 | P35548 |
| 977 | HTR6 | P50406 | MTAP | Q13126 |
| 978 | HRH2 | P25021 | RHOA | P61586 |
| 979 | PPP1CA | P62136 | CEBPA | P49715 |
| 980 | EPHX1 | P07099 | KLF4 | O43474 |
| 981 | RORB | Q92753 | ZBTB16 | Q05516 |
| 982 | TUBB | P07437 | PCK2 | Q16822 |
| 983 | TUBA4A | P68366 | SMAD2 | Q15796 |
| 984 | BRPF1 | P55201 | PDX1 | P52945 |
| 985 | KLK5 | Q9Y337 | ATF4 | P18848 |
| 986 | LRRK2 | Q5S007 | LPAR1 | Q92633 |
| 987 | ABCC9 | O60706 | NEDD4L | Q96PU5 |
| 988 | CDK7 | P50613 | MMP11 | P24347 |
| 989 | CCKAR | P32238 | SHC1 | P29353 |
| 990 | GPR139 | Q6DWJ6 | CCR6 | P51684 |
| 991 | MAPK11 | Q15759 | LOX | P28300 |
| 992 | CYP24A1 | Q07973 | ABCB11 | O95342 |
| 993 | CAPN2 | P17655 | LCN2 | P80188 |
| 994 | PPIA | P62937 | KLF6 | Q99612 |
| 995 | TNNI3K | Q59H18 | CCL20 | P78556 |
| 996 | GRK3 | P35626 | CD2 | P06729 |
| 997 | GRK5 | P34947 | SORT1 | Q99523 |
| 998 | PLK2 | Q9NYY3 | PLAGL1 | Q9UM63 |
| 999 | PTPRC | P08575 | POU1F1 | P28069 |
| 1000 | CACNA1S | Q13698 | LRP8 | Q14114 |
| 1001 | SIRT3 | Q9NTG7 | MSMO1 | Q15800 |
| 1002 |  |  | PRSS3 | P35030 |
| 1003 |  |  | PROX1 | Q92786 |
| 1004 |  |  | THPO | P40225 |
| 1005 |  |  | SELPLG | Q14242 |
| 1006 |  |  | CX3CL1 | P78423 |
| 1007 |  |  | LIPH | Q8WWY8 |
| 1008 |  |  | BTG1 | P62324 |
| 1009 |  |  | DOCK7 | Q96N67 |
| 1010 |  |  | CXCL16 | Q9H2A7 |
| 1011 |  |  | TPST1 | O60507 |
| 1012 |  |  | TPST2 | O60704 |
| 1013 |  |  | APOM | O95445 |
| 1014 |  |  | TTF2 | Q9UNY4 |
| 1015 |  |  | TXNDC5 | Q8NBS9 |
| 1016 |  |  | PROP1 | O75360 |
| 1017 |  |  | RARRES2 | Q99969 |
| 1018 |  |  | ACSM3 | Q53FZ2 |
| 1019 |  |  | ZNF202 | O95125 |
| 1020 |  |  | IL37 | Q9NZH6 |
| 1021 |  |  | LILRB5 | O75023 |
| 1022 |  |  | ASIP | P42127 |
| 1023 |  |  | KCTD10 | Q9H3F6 |
| 1024 |  |  | TIMD4 | Q96H15 |
| 1025 |  |  | UBL5 | Q9BZL1 |
| 1026 |  |  | PLA2G12B | Q9BX93 |
| 1027 |  |  | DEFA3 | P59666 |
| 1028 |  |  | ZPR1 | O75312 |
| 1029 |  |  | ERP29 | P30040 |
| 1030 |  |  | BUD13 | Q9BRD0 |
| 1031 |  |  | LEPROT | O15243 |
| 1032 |  |  | CSN1S1 | P47710 |
| 1033 |  |  | GYPE | P15421 |
| 1034 |  |  | ZGLP1 | P0C6A0 |
| 1035 |  |  | KRBOX4 | Q5JUW0 |
| 1036 |  |  | IGHE | P01854 |
| 1037 |  |  | LPAL2 | Q16609 |
| 1038 |  |  | SELENOO | Q9BVL4 |
| 1039 |  |  | SERPINF1 | P36955 |
| 1040 |  |  | IL17A | Q16552 |
| 1041 |  |  | ICAM3 | P32942 |
| 1042 |  |  | IL2RA | P01589 |
| 1043 |  |  | STAR | P49675 |
| 1044 |  |  | BMP2 | P12643 |
| 1045 |  |  | GP1BA | P07359 |
| 1046 |  |  | IL13 | P35225 |
| 1047 |  |  | IL5 | P05113 |
| 1048 |  |  | CIITA | P33076 |
| 1049 |  |  | CAST | P20810 |
| 1050 |  |  | FGF19 | O95750 |
| 1051 |  |  | ARNT | P27540 |
| 1052 |  |  | IL7 | P13232 |
| 1053 |  |  | CSF3 | P09919 |
| 1054 |  |  | CCL4 | P13236 |
| 1055 |  |  | RGN | Q15493 |
| 1056 |  |  | APLN | Q9ULZ1 |
| 1057 |  |  | SLC2A1 | P11166 |
| 1058 |  |  | CHUK | O15111 |
| 1059 |  |  | CYP11A1 | P05108 |
| 1060 |  |  | STK11 | Q15831 |
| 1061 |  |  | FOXO1 | Q12778 |
| 1062 |  |  | TFAP2A | P05549 |
| 1063 |  |  | TNFSF11 | O14788 |
| 1064 |  |  | CUBN | O60494 |
| 1065 |  |  | TXNRD2 | Q9NNW7 |
| 1066 |  |  | UCP2 | P55851 |
| 1067 |  |  | GFER | P55789 |
| 1068 |  |  | GANAB | Q14697 |
| 1069 |  |  | CORIN | Q9Y5Q5 |
| 1070 |  |  | PDGFA | P04085 |
| 1071 |  |  | GJA4 | P35212 |
| 1072 |  |  | LIF | P15018 |
| 1073 |  |  | PLIN2 | Q99541 |
| 1074 |  |  | GPAM | Q9HCL2 |
| 1075 |  |  | COL14A1 | Q05707 |
| 1076 |  |  | SST | P61278 |
| 1077 |  |  | S100A8 | P05109 |
| 1078 |  |  | CABIN1 | Q9Y6J0 |
| 1079 |  |  | DDAH2 | O95865 |
| 1080 |  |  | FAP | Q12884 |
| 1081 |  |  | ATL1 | Q8WXF7 |
| 1082 |  |  | CA10 | Q9NS85 |
| 1083 |  |  | MRPL13 | Q9BYD1 |
| 1084 |  |  | AHSP | Q9NZD4 |
| 1085 |  |  | COL20A1 | Q9P218 |
| 1086 |  |  | MTBP | Q96DY7 |
| 1087 |  |  | MYC | P01106 |
| 1088 |  |  | PLCG2 | P16885 |
| 1089 |  |  | CDH2 | P19022 |
| 1090 |  |  | SPARC | P09486 |
| 1091 |  |  | PDGFB | P01127 |
| 1092 |  |  | IL6R | P08887 |
| 1093 |  |  | SLC2A2 | P11168 |
| 1094 |  |  | SLC4A1 | P02730 |
| 1095 |  |  | APC | P25054 |
| 1096 |  |  | MYH9 | P35579 |
| 1097 |  |  | COL4A1 | P02462 |
| 1098 |  |  | MEF2A | Q02078 |
| 1099 |  |  | KCNJ5 | P48544 |
| 1100 |  |  | KCNJ11 | Q14654 |
| 1101 |  |  | TPH2 | Q8IWU9 |
| 1102 |  |  | LIFR | P42702 |
| 1103 |  |  | TNNT2 | P45379 |
| 1104 |  |  | USP7 | Q93009 |
| 1105 |  |  | PTGIS | Q16647 |
| 1106 |  |  | RUNX2 | Q13950 |
| 1107 |  |  | PLCD1 | P51178 |
| 1108 |  |  | HSPG2 | P98160 |
| 1109 |  |  | GATA2 | P23769 |
| 1110 |  |  | LPIN1 | Q14693 |
| 1111 |  |  | DNM1L | O00429 |
| 1112 |  |  | NFE2L2 | Q16236 |
| 1113 |  |  | FADD | Q13158 |
| 1114 |  |  | HNF1A | P20823 |
| 1115 |  |  | HLA-DRB1 | P01911 |
| 1116 |  |  | GNAI1 | P63096 |
| 1117 |  |  | CFLAR | O15519 |
| 1118 |  |  | ITGA2 | P17301 |
| 1119 |  |  | WRN | Q14191 |
| 1120 |  |  | SERPIND1 | P05546 |
| 1121 |  |  | NFATC2 | Q13469 |
| 1122 |  |  | SOS2 | Q07890 |
| 1123 |  |  | PKD2 | Q13563 |
| 1124 |  |  | ERCC1 | P07992 |
| 1125 |  |  | GFPT1 | Q06210 |
| 1126 |  |  | BIRC5 | O15392 |
| 1127 |  |  | NCF2 | P19878 |
| 1128 |  |  | ABCC8 | Q09428 |
| 1129 |  |  | HLA-G | P17693 |
| 1130 |  |  | CPB2 | Q96IY4 |
| 1131 |  |  | GLP1R | P43220 |
| 1132 |  |  | CALM2 | P0DP24 |
| 1133 |  |  | PPP3CB | P16298 |
| 1134 |  |  | TRAF2 | Q12933 |
| 1135 |  |  | IL1R2 | P27930 |
| 1136 |  |  | SIRT6 | Q8N6T7 |
| 1137 |  |  | NR5A2 | O00482 |
| 1138 |  |  | P2RY2 | P41231 |
| 1139 |  |  | PLAUR | Q03405 |
| 1140 |  |  | PKD1 | P98161 |
| 1141 |  |  | CCR7 | P32248 |
| 1142 |  |  | EIF2S1 | P05198 |
| 1143 |  |  | ADIPOR1 | Q96A54 |
| 1144 |  |  | HELLS | Q9NRZ9 |
| 1145 |  |  | GLRX | P35754 |
| 1146 |  |  | STMN1 | P16949 |
| 1147 |  |  | PTPN3 | P26045 |
| 1148 |  |  | IL12A | P29459 |
| 1149 |  |  | SERPINB2 | P05120 |
| 1150 |  |  | SLC2A5 | P22732 |
| 1151 |  |  | SOCS3 | O14543 |
| 1152 |  |  | AOC3 | Q16853 |
| 1153 |  |  | GAS6 | Q14393 |
| 1154 |  |  | MAP4K5 | Q9Y4K4 |
| 1155 |  |  | NFATC4 | Q14934 |
| 1156 |  |  | CTNS | O60931 |
| 1157 |  |  | CRTC2 | Q53ET0 |
| 1158 |  |  | HBEGF | Q99075 |
| 1159 |  |  | CGA | P01215 |
| 1160 |  |  | PPP3CC | P48454 |
| 1161 |  |  | IGFBP5 | P24593 |
| 1162 |  |  | TPR | P12270 |
| 1163 |  |  | LAMA2 | P24043 |
| 1164 |  |  | TRADD | Q15628 |
| 1165 |  |  | STT3B | Q8TCJ2 |
| 1166 |  |  | WWP2 | O00308 |
| 1167 |  |  | SFRP4 | Q6FHJ7 |
| 1168 |  |  | OSM | P13725 |
| 1169 |  |  | CLCNKB | P51801 |
| 1170 |  |  | HYOU1 | Q9Y4L1 |
| 1171 |  |  | MAP3K14 | Q99558 |
| 1172 |  |  | FOXO3 | O43524 |
| 1173 |  |  | BMP6 | P22004 |
| 1174 |  |  | LRP4 | O75096 |
| 1175 |  |  | DGAT2 | Q96PD7 |
| 1176 |  |  | NFATC3 | Q12968 |
| 1177 |  |  | NEU1 | Q99519 |
| 1178 |  |  | CNTF | P26441 |
| 1179 |  |  | GPD1L | Q8N335 |
| 1180 |  |  | TFPI2 | P48307 |
| 1181 |  |  | TFEB | P19484 |
| 1182 |  |  | PROCR | Q9UNN8 |
| 1183 |  |  | TRH | P20396 |
| 1184 |  |  | SEL1L | Q9UBV2 |
| 1185 |  |  | HSPA4 | P34932 |
| 1186 |  |  | CALM3 | P0DP25 |
| 1187 |  |  | LYVE1 | Q9Y5Y7 |
| 1188 |  |  | NDUFS4 | O43181 |
| 1189 |  |  | EIF4G2 | P78344 |
| 1190 |  |  | NIN | Q8N4C6 |
| 1191 |  |  | ADIPOR2 | Q86V24 |
| 1192 |  |  | CXCL5 | P42830 |
| 1193 |  |  | PTX3 | P26022 |
| 1194 |  |  | RECK | O95980 |
| 1195 |  |  | TNFSF12 | O43508 |
| 1196 |  |  | REG1A | P05451 |
| 1197 |  |  | CLDN16 | Q9Y5I7 |
| 1198 |  |  | GCKR | Q14397 |
| 1199 |  |  | CDKL1 | Q00532 |
| 1200 |  |  | CD68 | P34810 |
| 1201 |  |  | CDKN3 | Q16667 |
| 1202 |  |  | CEACAM3 | P40198 |
| 1203 |  |  | RAB28 | P51157 |
| 1204 |  |  | KLRK1 | P26718 |
| 1205 |  |  | PECAM1 | P16284 |
| 1206 |  |  | DNAJB11 | Q9UBS4 |
| 1207 |  |  | HPCAL1 | P37235 |
| 1208 |  |  | RNASE3 | P12724 |
| 1209 |  |  | ART3 | Q13508 |
| 1210 |  |  | IL23A | Q9NPF7 |
| 1211 |  |  | L2HGDH | Q9H9P8 |
| 1212 |  |  | REG3A | Q06141 |
| 1213 |  |  | MANF | P55145 |
| 1214 |  |  | CCN2 | P29279 |
| 1215 |  |  | EBI3 | Q14213 |
| 1216 |  |  | AKAP10 | O43572 |
| 1217 |  |  | CD177 | Q8N6Q3 |
| 1218 |  |  | SAA4 | P35542 |
| 1219 |  |  | SAV1 | Q9H4B6 |
| 1220 |  |  | IFI27 | P40305 |
| 1221 |  |  | GANC | Q8TET4 |
| 1222 |  |  | FAM3B | P58499 |
| 1223 |  |  | BLZF1 | Q9H2G9 |
| 1224 |  |  | CELA3B | P08861 |
| 1225 |  |  | CLEC12A | Q5QGZ9 |
| 1226 |  |  | FHOD3 | Q2V2M9 |
| 1227 |  |  | LACTB | P83111 |
| 1228 |  |  | SLC25A40 | Q8TBP6 |
| 1229 |  |  | MUC16 | Q8WXI7 |
| 1230 |  |  | MRGPRX1 | Q96LB2 |
| 1231 |  |  | NKX3-2 | P78367 |
| 1232 |  |  | NINJ2 | Q9NZG7 |
| 1233 |  |  | ARHGAP18 | Q8N392 |
| 1234 |  |  | CMTM8 | Q8IZV2 |
| 1235 |  |  | TIFA | Q96CG3 |
| 1236 |  |  | FAM3A | P98173 |
| 1237 |  |  | GALP | Q9UBC7 |
| 1238 |  |  | ELP1 | O95163 |
| 1239 |  |  | ESYT3 | A0FGR9 |
| 1240 |  |  | XKR6 | Q5GH73 |
| 1241 |  |  | CCN3 | P48745 |
| 1242 |  |  | KIAA1328 | Q86T90 |
| 1243 |  |  | ZNF860 | A6NHJ4 |
| 1244 |  |  | TPGS2 | Q68CL5 |
| 1245 |  |  | SERTAD2 | Q14140 |
| 1246 |  |  | SLC35G5 | Q96KT7 |
| 1247 |  |  | ANGPTL8 | Q6UXH0 |
| 1248 |  |  | MALRD1 | Q5VYJ5 |
| 1249 |  |  | DMAC2L | Q99766 |
| 1250 |  |  | HSP90AB2P | Q58FF8 |
| 1251 |  |  | PPARGC1B | Q86YN6 |
| 1252 |  |  | NEIL1 | Q96FI4 |
| 1253 |  |  | SMARCD1 | Q96GM5 |
| 1254 |  |  | SDC1 | P18827 |
| 1255 |  |  | EEF1A2 | Q05639 |
| 1256 |  |  | APRT | P07741 |
| 1257 |  |  | CNBP | P62633 |
| 1258 |  |  | ASRGL1 | Q7L266 |
| 1259 |  |  | ELF3 | P78545 |
| 1260 |  |  | DISC1 | Q9NRI5 |
| 1261 |  |  | INPP5F | Q9Y2H2 |
| 1262 |  |  | TNFRSF12A | Q9NP84 |
| 1263 |  |  | ACSL5 | Q9ULC5 |
| 1264 |  |  | CHDH | Q8NE62 |
| 1265 |  |  | JPH3 | Q8WXH2 |
| 1266 |  |  | WDHD1 | O75717 |
| 1267 |  |  | TNFRSF25 | Q93038 |
| 1268 |  |  | CDH13 | P55290 |
| 1269 |  |  | GREB1 | Q4ZG55 |
| 1270 |  |  | MVP | Q14764 |
| 1271 |  |  | CAD | P27708 |
| 1272 |  |  | KIDINS220 | Q9ULH0 |
| 1273 |  |  | MAGEE1 | Q9HCI5 |
| 1274 |  |  | SERPINA13P | Q6UXR4 |
| 1275 |  |  | ACADSB | P45954 |
| 1276 |  |  | GGTLC3 | B5MD39 |
| 1277 |  |  | GGT2 | P36268 |
| 1278 |  |  | NANOS3 | P60323 |
| 1279 |  |  | CDCP1 | Q9H5V8 |
| 1280 |  |  | FSD1 | Q9BTV5 |
| 1281 |  |  | CDC73 | Q6P1J9 |
| 1282 |  |  | FSD1L | Q9BXM9 |
| 1283 |  |  | HELZ2 | Q9BYK8 |
| 1284 |  |  | KLRC4-KLRK1 | H3BQV0 |
| 1285 |  |  | YWHAZ | P63104 |
| 1286 |  |  | NOV | Q9UIW2 |
| 1287 |  |  | NT5E | P21589 |
| 1288 |  |  | IRF6 | O14896 |
| 1289 |  |  | RPSA | P08865 |
| 1290 |  |  | ID2 | Q02363 |
| 1291 |  |  | HFE | Q30201 |
| 1292 |  |  | GLB1 | P16278 |
| 1293 |  |  | PLXNA1 | Q9VIW2 |
| 1294 |  |  | SDC2 | P34741 |
| 1295 |  |  | PTPRA | P18433 |
| 1296 |  |  | DECR1 | Q16698 |
| 1297 |  |  | RPL10 | P27635 |
