# Supplementary material for: Based on Network Pharmacology and RNA Sequencing Techniques to Explore the Molecular Mechanism of Huatan Jiangzhuo Decoction for Treating Hyperlipidemia
Source: Evid Based Complement Alternat Med. 2021 Apr 9;2021:9863714. doi: 10.1155/2021/9863714 (PMC8055390; doi:10.1155/2021/9863714)
Supplement: Supplementary Materials — Supplemental Table 1: 120 compounds of herbs in HTJZD meeting the criteria of OB ≥ 30% and DL ≥ 0.18 were picked up from TCMSP. Supplemental Table 2: 1001 compound-related targets (C-T) screened out from TCMSP, ETCM, and Swiss Target Prediction and 1297 disease-related targets (D-T) collected from DisGeNET and GeneCards. Supplemental Table 3: 202 regulated differentially expressed genes from RNA-seq analysis results. Supplemental Table 4: 301 targets of rat genomes mapped from the orthology of selected-targets (S-T) in human sapiens were obtained from HGNC. The supplemental materials are accessible to the interested readers on the website of Evidence-Based Complementary and Alternative Medicine. [file 9863714.f1.zip › 9863714.f1/9863714_Supplemental table 3 Xiaowen Zhou.docx]

**Supplemental table 3**. 202 regulated differentially expressed genes from RNA sequencing analysis results.

| Gene symbol | Fold Change  M VS N^[[1]](#footnote-1)^† | FDR  M VS N | Fold Change  H VS M^[[2]](#footnote-2)^‡ | FDR  H VS M |
| --- | --- | --- | --- | --- |
| Alas2 | 3.009 | 0.000 | 0.405 | 0.008 |
| Slc26a8 | 0.493 | 0.005 | 2.809 | 0.000 |
| Prf1 | 2.273 | 0.000 | 0.488 | 0.040 |
| Amd1 | 0.612 | 0.003 | 1.838 | 0.007 |
| Tmem119 | 2.829 | 0.000 | 0.468 | 0.040 |
| RT1-S3 | 1.654 | 0.002 | 0.550 | 0.040 |
| Fbxo21 | 0.396 | 0.000 | 2.273 | 0.005 |
| Trpm2 | 3.454 | 0.000 | 0.506 | 0.047 |
| Pcbp3 | 0.571 | 0.013 | 2.738 | 0.001 |
| Col6a2 | 1.799 | 0.000 | 0.634 | 0.019 |
| Hectd4 | 0.623 | 0.000 | 1.756 | 0.012 |
| Cyp3a62 | 3.904 | 0.000 | 0.497 | 0.014 |
| Eln | 1.639 | 0.018 | 0.460 | 0.002 |
| Sdf2l1 | 1.818 | 0.000 | 0.585 | 0.016 |
| Rcan1 | 1.531 | 0.021 | 0.296 | 0.000 |
| Igfbp7 | 1.994 | 0.000 | 0.648 | 0.041 |
| Maged2 | 1.663 | 0.001 | 0.412 | 0.000 |
| Tom1l1 | 0.644 | 0.005 | 1.637 | 0.026 |
| Myh10 | 2.360 | 0.000 | 0.516 | 0.020 |
| Olfml2b | 3.332 | 0.000 | 0.455 | 0.024 |
| Atp2b4 | 2.428 | 0.001 | 0.400 | 0.018 |
| Prelp | 1.804 | 0.000 | 0.553 | 0.002 |
| Slc4a4 | 0.655 | 0.016 | 1.695 | 0.019 |
| Rasd1 | 0.489 | 0.002 | 1.950 | 0.021 |
| Col3a1 | 2.033 | 0.000 | 0.508 | 0.001 |
| Srebf1 | 2.173 | 0.000 | 0.248 | 0.000 |
| Ephx1 | 2.119 | 0.000 | 0.555 | 0.003 |
| Col1a1 | 3.062 | 0.000 | 0.377 | 0.001 |
| Trib2 | 2.205 | 0.001 | 0.458 | 0.008 |
| Gpm6b | 2.545 | 0.001 | 0.477 | 0.030 |
| Acvr1c | 0.570 | 0.007 | 2.099 | 0.012 |
| B4galnt1 | 0.514 | 0.000 | 1.897 | 0.004 |
| Rprm | 3.985 | 0.000 | 0.122 | 0.000 |
| Tp53i3 | 1.650 | 0.000 | 0.599 | 0.013 |
| Nipal2 | 2.193 | 0.000 | 0.621 | 0.035 |
| Pamr1 | 1.719 | 0.003 | 0.589 | 0.050 |
| Mmp16 | 1.850 | 0.013 | 0.479 | 0.024 |
| Ispd | 1.546 | 0.010 | 0.447 | 0.001 |
| Pcsk9 | 0.442 | 0.003 | 3.606 | 0.000 |
| Mrc2 | 2.182 | 0.005 | 0.357 | 0.006 |
| Cdkn2b | 2.452 | 0.001 | 0.245 | 0.001 |
| Dhcr24 | 0.487 | 0.023 | 5.696 | 0.000 |
| Fem1b | 0.624 | 0.001 | 1.556 | 0.026 |
| Sbspon | 12.903 | 0.000 | 0.336 | 0.000 |
| Fbn1 | 1.951 | 0.000 | 0.616 | 0.019 |
| Trib3 | 2.026 | 0.000 | 0.475 | 0.029 |
| Fbln2 | 1.907 | 0.012 | 0.518 | 0.044 |
| Tmem64 | 0.515 | 0.000 | 1.697 | 0.013 |
| Zfp830 | 0.630 | 0.001 | 1.542 | 0.044 |
| C1qtnf5 | 2.111 | 0.000 | 0.550 | 0.030 |
| Tmem30b | 0.497 | 0.019 | 2.222 | 0.003 |
| Pnpla7 | 0.662 | 0.005 | 1.706 | 0.011 |
| Emilin1 | 1.688 | 0.000 | 0.564 | 0.017 |
| Tnfrsf11b | 2.298 | 0.000 | 0.409 | 0.001 |
| RGD1309079 | 1.548 | 0.050 | 0.419 | 0.019 |
| Mthfr | 0.643 | 0.007 | 1.979 | 0.020 |
| Slc9a9 | 1.652 | 0.001 | 0.527 | 0.015 |
| Loxl1 | 2.938 | 0.000 | 0.331 | 0.001 |
| Col5a1 | 1.685 | 0.000 | 0.546 | 0.003 |
| Acox1 | 0.592 | 0.001 | 2.031 | 0.007 |
| Tspan18 | 0.545 | 0.004 | 4.042 | 0.000 |
| Ass1 | 0.634 | 0.000 | 1.866 | 0.001 |
| Csrp1 | 1.525 | 0.003 | 0.533 | 0.023 |
| Rbm48 | 0.664 | 0.022 | 1.984 | 0.015 |
| Marcksl1 | 2.160 | 0.000 | 0.568 | 0.032 |
| LOC500007 | 0.526 | 0.015 | 2.413 | 0.011 |
| Hivep3 | 2.620 | 0.000 | 0.406 | 0.017 |
| Ripk2 | 5.618 | 0.000 | 0.385 | 0.022 |
| Ptprg | 0.602 | 0.000 | 1.763 | 0.001 |
| Cyp7a1 | 2.417 | 0.001 | 0.312 | 0.003 |
| Khdrbs3 | 1.732 | 0.037 | 0.353 | 0.012 |
| Pdxp | 0.246 | 0.000 | 3.323 | 0.020 |
| Cyp4a1 | 0.524 | 0.000 | 1.831 | 0.012 |
| Cyp4b1 | 0.537 | 0.000 | 1.762 | 0.044 |
| Lgals1 | 4.340 | 0.000 | 0.287 | 0.002 |
| Panx1 | 2.365 | 0.000 | 0.531 | 0.006 |
| Acot2 | 0.609 | 0.002 | 2.374 | 0.000 |
| Hdc | 6.904 | 0.000 | 0.225 | 0.000 |
| Thbs2 | 1.861 | 0.002 | 0.404 | 0.003 |
| Aig1 | 0.458 | 0.000 | 2.481 | 0.000 |
| Slc7a2 | 0.586 | 0.006 | 2.091 | 0.011 |
| MARCH 6 | 0.592 | 0.000 | 1.723 | 0.015 |
| Ackr4 | 0.589 | 0.013 | 2.261 | 0.020 |
| Aldh1b1 | 2.146 | 0.001 | 0.319 | 0.002 |
| Asah2 | 2.014 | 0.001 | 0.402 | 0.002 |
| Pcdh7 | 2.260 | 0.003 | 0.151 | 0.000 |
| Sparc | 1.623 | 0.000 | 0.656 | 0.026 |
| Aspg | 0.589 | 0.000 | 2.281 | 0.000 |
| Uap1l1 | 1.937 | 0.000 | 0.524 | 0.002 |
| Hyls1 | 0.546 | 0.023 | 2.305 | 0.049 |
| Ldha | 0.542 | 0.000 | 1.746 | 0.005 |
| Tnfsf10 | 2.204 | 0.008 | 0.357 | 0.026 |
| Tpcn2 | 0.651 | 0.000 | 1.714 | 0.003 |
| Sh2d4a | 1.887 | 0.000 | 0.455 | 0.000 |
| Cps1 | 0.642 | 0.002 | 1.765 | 0.008 |
| Mfsd2a | 0.565 | 0.009 | 2.074 | 0.014 |
| Smoc2 | 1.950 | 0.039 | 0.507 | 0.019 |
| Anxa5 | 2.839 | 0.000 | 0.497 | 0.008 |
| Wfdc2 | 3.312 | 0.002 | 0.558 | 0.037 |
| Peg3 | 0.550 | 0.000 | 1.936 | 0.007 |
| Pex11a | 0.500 | 0.000 | 2.186 | 0.002 |
| Il17rb | 0.457 | 0.001 | 2.874 | 0.000 |
| Tm4sf1 | 2.905 | 0.000 | 0.445 | 0.018 |
| Ptdss2 | 0.656 | 0.000 | 1.634 | 0.020 |
| Fzd1 | 0.449 | 0.000 | 2.138 | 0.001 |
| Ppp1r14c | 21.404 | 0.000 | 0.295 | 0.000 |
| Pdik1l | 0.640 | 0.005 | 1.767 | 0.045 |
| Itga8 | 1.607 | 0.029 | 0.566 | 0.047 |
| Mmp2 | 1.622 | 0.002 | 0.519 | 0.008 |
| Necab3 | 2.471 | 0.001 | 0.359 | 0.028 |
| Chka | 1.565 | 0.001 | 0.375 | 0.006 |
| Serpinh1 | 1.985 | 0.000 | 0.538 | 0.034 |
| Plekhs1 | 3.820 | 0.000 | 0.202 | 0.018 |
| Hfe | 1.527 | 0.004 | 0.636 | 0.018 |
| Afap1l2 | 1.770 | 0.041 | 0.415 | 0.033 |
| Prss23 | 1.692 | 0.000 | 0.465 | 0.000 |
| Rsrp1 | 0.625 | 0.008 | 2.568 | 0.000 |
| Irf7 | 2.438 | 0.001 | 0.449 | 0.029 |
| Exoc3l2 | 0.371 | 0.000 | 2.933 | 0.000 |
| Phyh | 0.491 | 0.000 | 1.528 | 0.043 |
| Bok | 2.933 | 0.000 | 0.355 | 0.004 |
| Gas6 | 2.342 | 0.000 | 0.460 | 0.000 |
| Cblc | 2.547 | 0.000 | 0.251 | 0.000 |
| Pde8a | 0.626 | 0.000 | 1.758 | 0.004 |
| Ldhd | 1.519 | 0.001 | 0.583 | 0.002 |
| Gstm3 | 8.344 | 0.000 | 0.307 | 0.000 |
| Emc9 | 1.510 | 0.007 | 0.534 | 0.008 |
| Olfml3 | 1.718 | 0.006 | 0.531 | 0.025 |
| Scnn1a | 0.514 | 0.002 | 2.003 | 0.041 |
| MGC108823 | 5.628 | 0.000 | 0.235 | 0.027 |
| Ramp1 | 1.687 | 0.004 | 0.468 | 0.002 |
| Tgfb1i1 | 2.571 | 0.000 | 0.472 | 0.033 |
| Myl9 | 1.737 | 0.000 | 0.573 | 0.022 |
| Pklr | 1.881 | 0.000 | 0.271 | 0.000 |
| Hcn3 | 2.002 | 0.000 | 0.238 | 0.000 |
| Ryr1 | 3.828 | 0.000 | 0.334 | 0.006 |
| Mcpt1 | 4.164 | 0.000 | 0.226 | 0.000 |
| Abcc8 | 1.735 | 0.029 | 0.227 | 0.000 |
| RGD1311739 | 1.600 | 0.013 | 0.551 | 0.042 |
| Cdc25b | 3.111 | 0.000 | 0.393 | 0.004 |
| Bmp2 | 0.527 | 0.000 | 1.793 | 0.011 |
| RGD1561849 | 3.451 | 0.000 | 0.179 | 0.000 |
| Mybl1 | 4.290 | 0.000 | 0.368 | 0.001 |
| Zbtb40 | 0.630 | 0.037 | 2.065 | 0.019 |
| RGD1309821 | 0.664 | 0.035 | 2.162 | 0.008 |
| Parp14 | 1.772 | 0.001 | 0.444 | 0.017 |
| LOC500300 | 2.438 | 0.001 | 0.429 | 0.027 |
| Ahctf1 | 0.626 | 0.001 | 1.518 | 0.049 |
| Sned1 | 0.549 | 0.003 | 6.061 | 0.000 |
| Pcolce | 2.543 | 0.000 | 0.484 | 0.014 |
| Lrat | 1.536 | 0.009 | 0.305 | 0.000 |
| Col14a1 | 2.198 | 0.000 | 0.565 | 0.013 |
| Bbs10 | 0.544 | 0.001 | 3.912 | 0.001 |
| Tmem186 | 0.659 | 0.006 | 1.801 | 0.024 |
| Birc6 | 0.627 | 0.001 | 1.817 | 0.007 |
| Zhx3 | 0.660 | 0.000 | 1.590 | 0.010 |
| Clec2l | 2.495 | 0.002 | 0.248 | 0.004 |
| LOC100365958 | 0.663 | 0.024 | 2.028 | 0.005 |
| Hba-a1 | 2.802 | 0.000 | 0.385 | 0.001 |
| Insr | 0.548 | 0.000 | 1.682 | 0.012 |
| Hacd3 | 0.625 | 0.000 | 1.866 | 0.004 |
| Plekhg2 | 1.512 | 0.008 | 0.476 | 0.004 |
| Cfap97 | 0.614 | 0.011 | 1.898 | 0.016 |
| Zrsr2 | 0.617 | 0.013 | 2.415 | 0.001 |
| Cd276 | 2.079 | 0.000 | 0.454 | 0.016 |
| Aox2 | 3.009 | 0.021 | 0.195 | 0.003 |
| Sectm1a | 1.567 | 0.019 | 0.436 | 0.034 |
| Pycr1 | 3.502 | 0.000 | 0.371 | 0.014 |
| Col8a1 | 2.587 | 0.000 | 0.318 | 0.000 |
| Clec4a1 | 2.663 | 0.000 | 0.449 | 0.006 |
| Mlkl | 2.516 | 0.000 | 0.394 | 0.002 |
| Rhof | 0.510 | 0.004 | 2.803 | 0.001 |
| Ncald | 1.989 | 0.001 | 0.489 | 0.011 |
| Epm2aip1 | 0.566 | 0.000 | 1.934 | 0.002 |
| Hba-a3 | 4.456 | 0.001 | 0.476 | 0.018 |
| Ppm1l | 0.595 | 0.001 | 2.385 | 0.000 |
| Cyp3a9 | 0.564 | 0.001 | 2.935 | 0.007 |
| Hba-a2 | 2.661 | 0.000 | 0.381 | 0.000 |
| Mest | 3.903 | 0.000 | 0.371 | 0.017 |
| Uhrf1 | 6.685 | 0.000 | 0.286 | 0.045 |
| Uhrf1bp1l | 0.628 | 0.000 | 1.650 | 0.016 |
| Fbln5 | 1.666 | 0.006 | 0.509 | 0.009 |
| Sepw1 | 1.984 | 0.000 | 0.592 | 0.012 |
| RGD1561277 | 0.576 | 0.037 | 2.739 | 0.012 |
| Manba | 1.925 | 0.000 | 0.493 | 0.010 |
| Epg5 | 0.600 | 0.000 | 1.718 | 0.008 |
| AC114111.1 | 0.499 | 0.005 | 1.927 | 0.029 |
| Acot3 | 0.253 | 0.000 | 2.026 | 0.023 |
| Ranbp6 | 0.573 | 0.000 | 2.406 | 0.000 |
| Lss | 0.436 | 0.000 | 2.449 | 0.000 |
| Gtf3c4 | 0.651 | 0.007 | 1.619 | 0.029 |
| LOC100912026 | 7.356 | 0.010 | 0.160 | 0.015 |
| Glud1 | 0.632 | 0.001 | 1.707 | 0.014 |
| Cav2 | 1.953 | 0.000 | 0.483 | 0.000 |
| Hbb | 3.403 | 0.000 | 0.331 | 0.000 |
| Popdc2 | 3.459 | 0.000 | 0.200 | 0.000 |
| Fam35a | 0.624 | 0.000 | 1.737 | 0.013 |
| Ept1 | 0.612 | 0.000 | 1.585 | 0.026 |
| Bst2 | 1.613 | 0.009 | 0.529 | 0.016 |
| Tuba1a | 2.567 | 0.000 | 0.547 | 0.032 |
| LOC103694857 | 3.188 | 0.000 | 0.306 | 0.000 |
| Gck | 2.876 | 0.000 | 0.257 | 0.000 |

1. † Model group versus Normal group [↑](#footnote-ref-1)
2. ‡ Huatan Jiangzhuo Decoction group versus Model group [↑](#footnote-ref-2)
