# Supplementary material for: Based on Network Pharmacology and RNA Sequencing Techniques to Explore the Molecular Mechanism of Huatan Jiangzhuo Decoction for Treating Hyperlipidemia
Source: Evid Based Complement Alternat Med. 2021 Apr 9;2021:9863714. doi: 10.1155/2021/9863714 (PMC8055390; doi:10.1155/2021/9863714)
Supplement: Supplementary Materials — Supplemental Table 1: 120 compounds of herbs in HTJZD meeting the criteria of OB ≥ 30% and DL ≥ 0.18 were picked up from TCMSP. Supplemental Table 2: 1001 compound-related targets (C-T) screened out from TCMSP, ETCM, and Swiss Target Prediction and 1297 disease-related targets (D-T) collected from DisGeNET and GeneCards. Supplemental Table 3: 202 regulated differentially expressed genes from RNA-seq analysis results. Supplemental Table 4: 301 targets of rat genomes mapped from the orthology of selected-targets (S-T) in human sapiens were obtained from HGNC. The supplemental materials are accessible to the interested readers on the website of Evidence-Based Complementary and Alternative Medicine. [file 9863714.f1.zip › 9863714.f1/9863714_Supplemental table 4 Xiaowen Zhou.docx]

**Supplemental table 4.** 301 targets of *R. norvegicus* genomes was mapped from the orthology of selected-targets (S-T) in *H. sapiens* obtained from the HGNC.

| Primary species | Ortholog species | Primary symbol | Ortholog symbol | Primary species DBID | Ortholog species DBID |
| --- | --- | --- | --- | --- | --- |
| Human | Rat | ABCB1 | Abcb1a | HGNC:40 | RGD:619951 |
| Human | Rat | ABCB1 | Abcb1b | HGNC:40 | RGD:3318 |
| Human | Rat | ABCB1 | Abcb4 | HGNC:40 |  |
| Human | Rat | ACACA | Acaca | HGNC:84 | RGD:621248 |
| Human | Rat | ACACA | Acacb | HGNC:84 | RGD:620500 |
| Human | Rat | ACE | Ace | HGNC:2707 | RGD:2493 |
| Human | Rat | ACSL3 | Acsl3 | HGNC:3570 | RGD:70552 |
| Human | Rat | ADIPOQ | Adipoq | HGNC:13633 | RGD:628748 |
| Human | Rat | ADRB2 | Adrb2 | HGNC:286 | RGD:2060 |
| Human | Rat | AGTR1 | Agtr1a | HGNC:336 | RGD:2070 |
| Human | Rat | AGTR1 | Agtr1b | HGNC:336 | RGD:2071 |
| Human | Rat | AHR | Ahr | HGNC:348 | RGD:2074 |
| Human | Rat | AKT1 | Akt1 | HGNC:391 | RGD:2081 |
| Human | Rat | AKT2 | Akt2 | HGNC:392 | RGD:2082 |
| Human | Rat | ALDH2 | Aldh2 | HGNC:404 | RGD:69219 |
| Human | Rat | ALDH2 | Aldh1b1 | HGNC:404 | RGD:1306737 |
| Human | Rat | ALOX12 | Alox12 | HGNC:429 | RGD:1311159 |
| Human | Rat | ALOX5 | Alox5 | HGNC:435 | RGD:2096 |
| Human | Rat | ALOX5 | Alox12b | HGNC:435 | RGD:1305330 |
| Human | Rat | ALOX5 | Alox15b | HGNC:435 | RGD:628809 |
| Human | Rat | ALOX5 | Aloxe3 | HGNC:435 | RGD:1306252 |
| Human | Rat | APOB | Apob | HGNC:603 | RGD:2129 |
| Human | Rat | APP | App | HGNC:620 | RGD:2139 |
| Human | Rat | AR | Ar | HGNC:644 | RGD:2147 |
| Human | Rat | AURKA | Aurka | HGNC:11393 | RGD:628895 |
| Human | Rat | BAX | Bax | HGNC:959 | RGD:2192 |
| Human | Rat | BCL2 | Bcl2 | HGNC:990 | RGD:2199 |
| Human | Rat | BRAF | Braf | HGNC:1097 | RGD:619908 |
| Human | Rat | C5AR1 | C5ar1 | HGNC:1338 | RGD:70553 |
| Human | Rat | C5AR1 | C5ar2 | HGNC:1338 | RGD:1303027 |
| Human | Rat | CALM1 | Calm1 | HGNC:1442 | RGD:2257 |
| Human | Rat | CALM1 | Calm2 | HGNC:1442 | RGD:2258 |
| Human | Rat | CALM1 | Calm3 | HGNC:1442 | RGD:2259 |
| Human | Rat | CALM1 | AABR07013776.1 | HGNC:1442 | RGD:15005429 |
| Human | Rat | CASP1 | Casp1 | HGNC:1499 | RGD:2274 |
| Human | Rat | CASP3 | Casp3 | HGNC:1504 | RGD:2275 |
| Human | Rat | CASP8 | Casp8 | HGNC:1509 | RGD:620945 |
| Human | Rat | CASP9 | Casp9 | HGNC:1511 | RGD:61867 |
| Human | Rat | CAT | Cat | HGNC:1516 | RGD:2279 |
| Human | Rat | CCL2 | Ccl12 | HGNC:10618 | RGD:1309255 |
| Human | Rat | CCL2 | Ccl2 | HGNC:10618 | RGD:3645 |
| Human | Rat | CCL2 | Ccl11 | HGNC:10618 | RGD:3644 |
| Human | Rat | CCL2 | Ccl7 | HGNC:10618 | RGD:1359152 |
| Human | Rat | CCND1 | Ccnd1 | HGNC:1582 | RGD:68384 |
| Human | Rat | CCND1 | Ccnd2 | HGNC:1582 | RGD:621083 |
| Human | Rat | CCND1 | Ccnd3 | HGNC:1582 | RGD:2293 |
| Human | Rat | CCR2 | Ccr5 | HGNC:1603 | RGD:620596 |
| Human | Rat | CCR3 | Ccr3 | HGNC:1604 | RGD:620593 |
| Human | Rat | CCR5 | Ccr2 | HGNC:1606 | RGD:620876 |
| Human | Rat | CDK1 | Cdk1 | HGNC:1722 | RGD:2319 |
| Human | Rat | CES1 | Ces1d | HGNC:1863 | RGD:70896 |
| Human | Rat | CES1 | Ces1a | HGNC:1863 | RGD:1583671 |
| Human | Rat | CES1 | Ces1c | HGNC:1863 | RGD:2571 |
| Human | Rat | CES1 | Ces1e | HGNC:1863 | RGD:621508 |
| Human | Rat | CES1 | Ces1f | HGNC:1863 | RGD:1642419 |
| Human | Rat | CES1 | LOC501233 | HGNC:1863 |  |
| Human | Rat | CFD | Cfd | HGNC:2771 | RGD:2498 |
| Human | Rat | COX5A | Cox5a | HGNC:2267 | RGD:620607 |
| Human | Rat | CPT1A | Cpt1a | HGNC:2328 | RGD:2396 |
| Human | Rat | CPT1A | Cpt1b | HGNC:2328 | RGD:2397 |
| Human | Rat | CREB1 | Creb1 | HGNC:2345 | RGD:620218 |
| Human | Rat | CREB1 | Atf1 | HGNC:2345 | RGD:1307360 |
| Human | Rat | CREBBP | Crebbp | HGNC:2348 | RGD:2401 |
| Human | Rat | CYP19A1 | Cyp19a1 | HGNC:2594 | RGD:2457 |
| Human | Rat | CYP2C19 | Cyp2c6v1 | HGNC:2621 | RGD:619934 |
| Human | Rat | CYP2C19 | AABR07004560.1 | HGNC:2621 | RGD:15004954 |
| Human | Rat | CYP2C19 | Cyp2c13 | HGNC:2621 | RGD:620363 |
| Human | Rat | CYP2C19 | Cyp2c24 | HGNC:2621 | RGD:1563697 |
| Human | Rat | CYP2C19 | Cyp2c11 | HGNC:2621 | RGD:2469 |
| Human | Rat | CYP2C19 | Cyp2c12 | HGNC:2621 | RGD:2470 |
| Human | Rat | CYP2C19 | Cyp2c22 | HGNC:2621 | RGD:620368 |
| Human | Rat | CYP2C19 | Cyp2c7 | HGNC:2621 | RGD:620379 |
| Human | Rat | CYP2C19 | LOC100361547 | HGNC:2621 | RGD:2319920 |
| Human | Rat | CYP3A4 | Cyp3a62 | HGNC:2637 | RGD:1595919 |
| Human | Rat | CYP3A4 | Cyp3a18 | HGNC:2637 | RGD:628709 |
| Human | Rat | CYP3A4 | Cyp3a9 | HGNC:2637 | RGD:708392 |
| Human | Rat | CYP3A4 | LOC100910877 | HGNC:2637 | RGD:6495986 |
| Human | Rat | CYP3A4 | Cyp3a2 | HGNC:2637 | RGD:708379 |
| Human | Rat | CYP3A4 | Cyp3a23-3a1 | HGNC:2637 | RGD:628626 |
| Human | Rat | CYP3A4 | Tbxas1 | HGNC:2637 | RGD:3826 |
| Human | Rat | CYP7A1 | Cyp7a1 | HGNC:2651 | RGD:2482 |
| Human | Rat | DNMT1 | Dnmt1 | HGNC:2976 | RGD:620979 |
| Human | Rat | EGFR | Egfr | HGNC:3236 | RGD:2543 |
| Human | Rat | EGFR | Erbb4 | HGNC:3236 | RGD:620486 |
| Human | Rat | EPHB2 | Ephb2 | HGNC:3393 | RGD:1564232 |
| Human | Rat | EPHB2 | Ephb1 | HGNC:3393 | RGD:2556 |
| Human | Rat | EPHB2 | Ephb3 | HGNC:3393 | RGD:1305602 |
| Human | Rat | ERBB2 | Erbb2 | HGNC:3430 | RGD:2561 |
| Human | Rat | ERBB2 | LOC102552659 | HGNC:3430 | RGD:7499005 |
| Human | Rat | ESR1 | Esr1 | HGNC:3467 | RGD:2581 |
| Human | Rat | ESR2 | Esr2 | HGNC:3468 | RGD:2582 |
| Human | Rat | F10 | F10 | HGNC:3528 | RGD:61850 |
| Human | Rat | F10 | F9 | HGNC:3528 | RGD:2589 |
| Human | Rat | F2 | F2 | HGNC:3535 | RGD:61996 |
| Human | Rat | F3 | F3 | HGNC:3541 | RGD:2587 |
| Human | Rat | FABP1 | Fabp1 | HGNC:3555 | RGD:2590 |
| Human | Rat | FABP4 | Fabp4 | HGNC:3559 | RGD:69309 |
| Human | Rat | FABP4 | Fabp9 | HGNC:3559 | RGD:620285 |
| Human | Rat | FABP4 | Pmp2 | HGNC:3559 | RGD:1585218 |
| Human | Rat | FADS1 | Fads1 | HGNC:3574 | RGD:621678 |
| Human | Rat | FADS1 | Fads3 | HGNC:3574 | RGD:628876 |
| Human | Rat | FADS1 | Fads2 | HGNC:3574 | RGD:68339 |
| Human | Rat | FADS1 | Fads2b | HGNC:3574 | RGD:1311224 |
| Human | Rat | FASN | Fasn | HGNC:3594 | RGD:620665 |
| Human | Rat | FDFT1 | Fdft1 | HGNC:3629 | RGD:61834 |
| Human | Rat | FECH | Fech | HGNC:3647 | RGD:1307556 |
| Human | Rat | FGF2 | Fgf2 | HGNC:3676 | RGD:2609 |
| Human | Rat | FGFR1 | Fgfr1 | HGNC:3688 | RGD:620713 |
| Human | Rat | FGFR1 | Fgfr2 | HGNC:3688 | RGD:2611 |
| Human | Rat | FKBP1A | Fkbp1a | HGNC:3711 | RGD:2617 |
| Human | Rat | FKBP1A | Fkbp1b | HGNC:3711 | RGD:61835 |
| Human | Rat | FLT1 | Flt1 | HGNC:3763 | RGD:2621 |
| Human | Rat | FN1 | Fn1 | HGNC:3778 | RGD:2624 |
| Human | Rat | FOS | Fos | HGNC:3796 | RGD:2626 |
| Human | Rat | FURIN | Furin | HGNC:8568 | RGD:3274 |
| Human | Rat | G6PD | G6pd | HGNC:4057 | RGD:2645 |
| Human | Rat | GCK | Gck | HGNC:4195 | RGD:2670 |
| Human | Rat | GSK3B | Gsk3b | HGNC:4617 | RGD:70982 |
| Human | Rat | GUSB | Gusb | HGNC:4696 | RGD:2772 |
| Human | Rat | GZMB | Gzmb | HGNC:4709 | RGD:620018 |
| Human | Rat | GZMB | Gzmbl3 | HGNC:4709 | RGD:2320502 |
| Human | Rat | GZMB | Gzmbl2 | HGNC:4709 | RGD:2320097 |
| Human | Rat | GZMB | Gzmc | HGNC:4709 | RGD:620019 |
| Human | Rat | GZMB | Gzmbl1 | HGNC:4709 | RGD:1561819 |
| Human | Rat | GZMB | Gzmf | HGNC:4709 | RGD:628603 |
| Human | Rat | GZMB | LOC691695 | HGNC:4709 | RGD:1597199 |
| Human | Rat | GZMB | Gzmn | HGNC:4709 | RGD:1597226 |
| Human | Rat | GZMB | LOC100910060 | HGNC:4709 | RGD:6500474 |
| Human | Rat | GZMB | LOC691670 | HGNC:4709 | RGD:1597224 |
| Human | Rat | GZMB | AABR07018028.1 | HGNC:4709 |  |
| Human | Rat | GZMB | Mcpt8l3 | HGNC:4709 |  |
| Human | Rat | HIF1A | Hif1a | HGNC:4910 | RGD:61928 |
| Human | Rat | HMGCR | Hmgcr | HGNC:5006 | RGD:2803 |
| Human | Rat | HMOX1 | Hmox1 | HGNC:5013 | RGD:2806 |
| Human | Rat | HSP90AA1 | Hsp90aa1 | HGNC:5253 | RGD:631409 |
| Human | Rat | HSP90AA1 | LOC103692716 | HGNC:5253 | RGD:9292348 |
| Human | Rat | HSP90AA1 | Hsp90ab1 | HGNC:5253 | RGD:1303075 |
| Human | Rat | HSP90B1 | Hsp90b1 | HGNC:12028 | RGD:1310482 |
| Human | Rat | ICAM1 | Icam1 | HGNC:5344 | RGD:2857 |
| Human | Rat | ICAM1 | Icam5 | HGNC:5344 | RGD:1305039 |
| Human | Rat | IFNG | Ifng | HGNC:5438 | RGD:2866 |
| Human | Rat | IGF2 | Igf2 | HGNC:5466 | RGD:2870 |
| Human | Rat | IGFBP3 | Igfbp3 | HGNC:5472 | RGD:2874 |
| Human | Rat | IGFBP3 | Igfbp5 | HGNC:5472 | RGD:2876 |
| Human | Rat | IL1B | Il1b | HGNC:5992 | RGD:2891 |
| Human | Rat | IL2 | Il2 | HGNC:6001 | RGD:620047 |
| Human | Rat | IL4 | Il4 | HGNC:6014 | RGD:2898 |
| Human | Rat | IL6 | Il6 | HGNC:6018 | RGD:2901 |
| Human | Rat | INS | Ins2 | HGNC:6081 | RGD:2916 |
| Human | Rat | INS | Ins1 | HGNC:6081 | RGD:2915 |
| Human | Rat | INSR | Insr | HGNC:6091 | RGD:2917 |
| Human | Rat | ITGAL | Itgal | HGNC:6148 | RGD:631424 |
| Human | Rat | ITGB2 | Itgb2 | HGNC:6155 | RGD:1305581 |
| Human | Rat | JAK3 | Jak3 | HGNC:6193 | RGD:2940 |
| Human | Rat | KIT | Kit | HGNC:6342 | RGD:620568 |
| Human | Rat | LDLR | Ldlr | HGNC:6547 | RGD:2998 |
| Human | Rat | LPL | Lpl | HGNC:6677 | RGD:3017 |
| Human | Rat | MAP2K1 | Map2k1 | HGNC:6840 | RGD:70495 |
| Human | Rat | MAP2K1 | Map2k2 | HGNC:6840 | RGD:61888 |
| Human | Rat | MAPK1 | Mapk1 | HGNC:6871 | RGD:70500 |
| Human | Rat | MAPK1 | Mapk3 | HGNC:6871 | RGD:3046 |
| Human | Rat | MAPK14 | Mapk14 | HGNC:6876 | RGD:70496 |
| Human | Rat | MAPK14 | Mapk11 | HGNC:6876 | RGD:1309340 |
| Human | Rat | MAPK8 | Mapk8 | HGNC:6881 | RGD:621506 |
| Human | Rat | MAPK8 | Mapk10 | HGNC:6881 | RGD:3663 |
| Human | Rat | MDM2 | Mdm2 | HGNC:6973 | RGD:1305332 |
| Human | Rat | MMP1 | Mmp1 | HGNC:7155 | RGD:1307917 |
| Human | Rat | MMP1 | Mmp1b | HGNC:7155 | RGD:1306843 |
| Human | Rat | MMP1 | AABR07069011.1 | HGNC:7155 | RGD:15009815 |
| Human | Rat | MMP1 | Mmp8 | HGNC:7155 | RGD:631408 |
| Human | Rat | MMP14 | Mmp14 | HGNC:7160 | RGD:620198 |
| Human | Rat | MMP2 | Mmp2 | HGNC:7166 | RGD:621316 |
| Human | Rat | MMP3 | Mmp3 | HGNC:7173 | RGD:621317 |
| Human | Rat | MMP3 | Mmp10 | HGNC:7173 | RGD:620192 |
| Human | Rat | MMP7 | Mmp7 | HGNC:7174 | RGD:3100 |
| Human | Rat | MMP9 | Mmp9 | HGNC:7176 | RGD:621320 |
| Human | Rat | MT-CO1 | Mt-co1 | HGNC:7419 | RGD:621871 |
| Human | Rat | MT-CO2 | Mt-co2 | HGNC:7421 | RGD:621872 |
| Human | Rat | MTOR | Mtor | HGNC:3942 | RGD:68371 |
| Human | Rat | NCOA1 | Ncoa1 | HGNC:7668 | RGD:1309046 |
| Human | Rat | NCOA2 | Ncoa2 | HGNC:7669 | RGD:620108 |
| Human | Rat | NFATC1 | Nfatc1 | HGNC:7775 | RGD:2319357 |
| Human | Rat | NFKB1 | Nfkb1 | HGNC:7794 | RGD:70498 |
| Human | Rat | NFKBIA | Nfkbia | HGNC:7797 | RGD:3171 |
| Human | Rat | NOS2 | Nos2 | HGNC:7873 | RGD:3185 |
| Human | Rat | NOS2 | LOC497963 | HGNC:7873 | RGD:1598227 |
| Human | Rat | NOS2 | Nos1 | HGNC:7873 | RGD:3184 |
| Human | Rat | NOS2 | Nos3 | HGNC:7873 | RGD:3186 |
| Human | Rat | NR1H2 | Nr1h2 | HGNC:7965 | RGD:61906 |
| Human | Rat | NR1H3 | Nr1h3 | HGNC:7966 | RGD:61909 |
| Human | Rat | NR1H4 | Nr1h4 | HGNC:7967 | RGD:628831 |
| Human | Rat | NR3C1 | Nr3c1 | HGNC:7978 | RGD:2741 |
| Human | Rat | NR3C1 | AABR07031756.1 | HGNC:7978 | RGD:15005607 |
| Human | Rat | PCSK9 | Pcsk9 | HGNC:20001 | RGD:728909 |
| Human | Rat | PIK3CA | Pik3ca | HGNC:8975 | RGD:620916 |
| Human | Rat | PIK3CA | LOC685590 | HGNC:8975 | RGD:1592558 |
| Human | Rat | PIK3CB | Pik3cb | HGNC:8976 | RGD:620917 |
| Human | Rat | PIK3CB | LOC100910021 | HGNC:8976 | RGD:6500836 |
| Human | Rat | PIK3CG | Pik3cg | HGNC:8978 | RGD:1306468 |
| Human | Rat | PLA2G1B | Pla2g1b | HGNC:9030 | RGD:61949 |
| Human | Rat | PLAT | Plat | HGNC:9051 | RGD:3342 |
| Human | Rat | PLAT | LOC100910418 | HGNC:9051 | RGD:6498623 |
| Human | Rat | PLAU | Plau | HGNC:9052 | RGD:3343 |
| Human | Rat | PLG | Plg | HGNC:9071 | RGD:619893 |
| Human | Rat | PPARA | Ppara | HGNC:9232 | RGD:3369 |
| Human | Rat | PPARD | Ppard | HGNC:9235 | RGD:3370 |
| Human | Rat | PPARG | Pparg | HGNC:9236 | RGD:3371 |
| Human | Rat | PPP3CA | Ppp3ca | HGNC:9314 | RGD:3382 |
| Human | Rat | PPP3CA | Ppp3cb | HGNC:9314 | RGD:3383 |
| Human | Rat | PPP3CA | Ppp3cc | HGNC:9314 | RGD:621616 |
| Human | Rat | PRKCB | Prkcb | HGNC:9395 | RGD:3396 |
| Human | Rat | PRKCB | Prkca | HGNC:9395 | RGD:3395 |
| Human | Rat | PRKCB | Prkcg | HGNC:9395 | RGD:3397 |
| Human | Rat | PRKCD | Prkcd | HGNC:9399 | RGD:67383 |
| Human | Rat | PSEN1 | Psen1 | HGNC:9508 | RGD:3425 |
| Human | Rat | PSMA3 | Psma3 | HGNC:9532 | RGD:61844 |
| Human | Rat | PSMA4 | Psma4 | HGNC:9533 | RGD:61846 |
| Human | Rat | PSMB5 | Psmb5 | HGNC:9542 | RGD:61879 |
| Human | Rat | PSMC4 | Psmc4 | HGNC:9551 | RGD:621102 |
| Human | Rat | PSMD7 | Psmd7 | HGNC:9565 | RGD:1306902 |
| Human | Rat | PYGL | Pygl | HGNC:9725 | RGD:620687 |
| Human | Rat | RARA | Rara | HGNC:9864 | RGD:3534 |
| Human | Rat | RARA | Rarb | HGNC:9864 | RGD:3535 |
| Human | Rat | RARA | Rarg | HGNC:9864 | RGD:1583230 |
| Human | Rat | RBP4 | Rbp4 | HGNC:9922 | RGD:3546 |
| Human | Rat | RELA | Rela | HGNC:9955 | RGD:727889 |
| Human | Rat | RXRA | Rxra | HGNC:10477 | RGD:3610 |
| Human | Rat | RXRA | Rxrg | HGNC:10477 | RGD:620046 |
| Human | Rat | RXRB | Rxrb | HGNC:10478 | RGD:3611 |
| Human | Rat | SERPINE1 | Serpine1 | HGNC:8583 | RGD:3249 |
| Human | Rat | SIRT1 | Sirt1 | HGNC:14929 | RGD:1308542 |
| Human | Rat | TGFB1 | Tgfb1 | HGNC:11766 | RGD:69051 |
| Human | Rat | TIMP1 | Timp1 | HGNC:11820 | RGD:621675 |
| Human | Rat | TLR4 | Tlr4 | HGNC:11850 | RGD:3870 |
| Human | Rat | TNF | Tnf | HGNC:11892 | RGD:3876 |
| Human | Rat | TNF | LOC103694380 | HGNC:11892 | RGD:9404643 |
| Human | Rat | TNFRSF10A | AABR07018323.1 | HGNC:11904 | RGD:15004308 |
| Human | Rat | TNFRSF10A | Tnfrsf10b | HGNC:11904 | RGD:1308041 |
| Human | Rat | TNFRSF1A | Tnfrsf1a | HGNC:11916 | RGD:621237 |
| Human | Rat | TP53 | Tp53 | HGNC:11998 | RGD:3889 |
| Human | Rat | TP53 | AABR07008242.1 | HGNC:11998 | RGD:15005090 |
| Human | Rat | PRKAA2 | Prkaa2 | HGNC:9377 | RGD:620893 |
| Human | Rat | REN | Ren | HGNC:9958 | RGD:3554 |
| Human | Rat | CFTR | Cftr | HGNC:1884 | RGD:2332 |
| Human | Rat | SOD1 | Sod1 | HGNC:11179 | RGD:3731 |
| Human | Rat | PIK3R1 | Pik3r1 | HGNC:8979 | RGD:3329 |
| Human | Rat | VEGFA | Vegfa | HGNC:12680 | RGD:619991 |
| Human | Rat | PTGS2 | Ptgs2 | HGNC:9605 | RGD:620349 |
| Human | Rat | PTGS2 | Ptgs1 | HGNC:9605 | RGD:3439 |
| Human | Rat | ALOX5AP | Alox5ap | HGNC:436 | RGD:2097 |
| Human | Rat | VLDLR | Vldlr | HGNC:12698 | RGD:3963 |
| Human | Rat | MTTP | Mttp | HGNC:7467 | RGD:1308388 |
| Human | Rat | TTR | Ttr | HGNC:12405 | RGD:3916 |
| Human | Rat | PSEN2 | Psen2 | HGNC:9509 | RGD:621060 |
| Human | Rat | NR0B1 | Nr0b1 | HGNC:7960 | RGD:62028 |
| Human | Rat | MYLK | Mylk | HGNC:7590 | RGD:1310915 |
| Human | Rat | NLRP3 | Nlrp3 | HGNC:16400 | RGD:1308314 |
| Human | Rat | NLRP3 | Nlrp1a | HGNC:16400 | RGD:1310963 |
| Human | Rat | MAPT | Mapt | HGNC:6893 | RGD:69329 |
| Human | Rat | XIAP | Xiap | HGNC:592 | RGD:620692 |
| Human | Rat | GSR | Gsr | HGNC:4623 | RGD:621747 |
| Human | Rat | CDK4 | Cdk4 | HGNC:1773 | RGD:621120 |
| Human | Rat | MT-CO3 | Mt-co3 | HGNC:7422 | RGD:621873 |
| Human | Rat | HDAC9 | Hdac9 | HGNC:14065 | RGD:1310748 |
| Human | Rat | VDR | Vdr | HGNC:12679 | RGD:3959 |
| Human | Rat | CXCR1 | Cxcr1 | HGNC:6026 | RGD:2905 |
| Human | Rat | CXCR1 | Cxcr2 | HGNC:6026 | RGD:2906 |
| Human | Rat | EPHX2 | Ephx2 | HGNC:3402 | RGD:620732 |
| Human | Rat | NR1I2 | Nr1i2 | HGNC:7968 | RGD:69057 |
| Human | Rat | UGT1A1 | Ugt1a1 | HGNC:12530 | RGD:3935 |
| Human | Rat | UGT1A1 | Ugt1a6 | HGNC:12530 |  |
| Human | Rat | PTK2B | Ptk2b | HGNC:9612 | RGD:628758 |
| Human | Rat | F7 | F7 | HGNC:3544 | RGD:628678 |
| Human | Rat | SLC27A1 | Slc27a1 | HGNC:10995 | RGD:620927 |
| Human | Rat | SLC27A1 | Slc27a4 | HGNC:10995 | RGD:1307383 |
| Human | Rat | LIPE | Lipe | HGNC:6621 | RGD:3010 |
| Human | Rat | SREBF1 | Srebf1 | HGNC:11289 | RGD:69423 |
| Human | Rat | SREBF2 | Srebf2 | HGNC:11290 | RGD:1307751 |
| Human | Rat | PPP3R1 | Ppp3r1 | HGNC:9317 | RGD:69230 |
| Human | Rat | PPP3R1 | AABR07016428.1 | HGNC:9317 | RGD:15010924 |
| Human | Rat | PPP3R1 | Ppp3r2 | HGNC:9317 | RGD:69232 |
| Human | Rat | PGD | Pgd | HGNC:8891 | RGD:1583832 |
| Human | Rat | WNT3A | Wnt3a | HGNC:15983 | RGD:1308057 |
| Human | Rat | GYS1 | Gys1 | HGNC:4706 | RGD:1589798 |
| Human | Rat | MPO | Mpo | HGNC:7218 | RGD:1592081 |
| Human | Rat | MPO | Epx | HGNC:7218 | RGD:1311882 |
| Human | Rat | MPO | Lpo | HGNC:7218 | RGD:1310051 |
| Human | Rat | PRF1 | Prf1 | HGNC:9360 | RGD:708463 |
| Human | Rat | LIMK1 | Limk1 | HGNC:6613 | RGD:62055 |
| Human | Rat | NR3C2 | Nr3c2 | HGNC:7979 | RGD:3094 |
| Human | Rat | SELE | Sele | HGNC:10718 | RGD:3654 |
| Human | Rat | IL6ST | Il6st | HGNC:6021 | RGD:2903 |
| Human | Rat | SLC2A4 | Slc2a4 | HGNC:11009 | RGD:2711 |
| Human | Rat | SLC2A4 | Slc2a1 | HGNC:11009 | RGD:3704 |
| Human | Rat | VCAM1 | Vcam1 | HGNC:12663 | RGD:3952 |
| Human | Rat | THRA | Thra | HGNC:11796 | RGD:3857 |
| Human | Rat | THRB | Thrb | HGNC:11799 | RGD:3858 |
| Human | Rat | PLA2G7 | Pla2g7 | HGNC:9040 | RGD:1310734 |
| Human | Rat | PSMD8 | Psmd8 | HGNC:9566 | RGD:1309842 |
| Human | Rat | PYGM | Pygm | HGNC:9726 | RGD:3461 |
| Human | Rat | PYGM | Pygb | HGNC:9726 | RGD:3460 |
